# Supplementary material for: Adherence to clinical practice guidelines for South Australian pregnant women with cardiac conditions between 2003 and 2013
Source: PLoS One. 2020 Mar 17;15(3):e0230459. doi: 10.1371/journal.pone.0230459 (PMC7077829; doi:10.1371/journal.pone.0230459)
Supplement: S1 Table — (PDF) [file pone.0230459.s001.pdf]

Supplementary 1 Code Log for Auditmaker and SAS scoring for adherence to the Statewide Perinatal Guidelines

Auditmaker Variable	SPSS Variable name / SAS code	Coding instructions /SAS Scoring for Obstetric /Cardiac Guideline adherence.	Adherence score
1. Study ID	ID	The number assigned to each case note	NA
2. **Hospital site	Site	1=Tertiary Hospital level 5 care<br>2=Quaternary Hospital<br>3=Maternity stand alone unit	NA
3. **Pre-natal care**<br>Pre-conception counselling documented	Preconception	Where documented evidence of preconception counselling then<br>**Total Score =+ 1 for Yes**<br>1=yes<br>2=No<br>3= Not applicable<br>4= Unknown	1
4. **Initial Ante-Natal Aassessment:**<br>Comprehensive physical assessment where 10 Components included:<br>1. Weight<br>2. Height<br>3. BMI<br>4. Oral health<br>5. Respiratory examination (auscultation)<br>6. Breast Examination<br>7. Abdominal Examination<br>8. Vaginal Examination<br>9. Urinalysis<br>10. Psychosocial Assessment<br>11. Not applicable<br>12. Unknown	See subsequent rows where coded and scored separately as ANC 1, ANC2 and ANC 3.	1. Weight, Height, BMI.<br>2. Oral health /Respiratory (auscultation) Breast Examination<br>3. Abdominal /Vaginal Examination/Urinalysis<br>4. Psychosocial Assessment<br>5. Not applicable	*See below*
- Initial Antenatal assessment includes 1-3 components	ANC1	Weight, Height and BMI where ANC1 =3 then<br>**Total Score =+1**	1

Supplementary 1 Code Log for Auditmaker and SAS scoring for adherence to the Statewide Perinatal Guidelines

**Auditmaker Variable**	**SPSS Variable name / SAS code**	**Coding instructions /SAS Scoring for Obstetric/Cardiac Guideline adherence.**	**Adherence score**
- Initial antenatal assessment includes components 4-6	ANC2	Oral health, respiratory and breast examination where ANC2=3 then<br>**Total Score = +1**	1
- Initial Antenatal assessment includes 7-9	ANC3	Abdominal, vaginal, and urinalysis assessment where ANC3=3 then<br>**Total Score = +1**	1
**Antenatal Mental Health assessment**<br>- Psychosocial Assessment 1 recorded	ANRQ score recorded	Antenatal Risk Questionnaire score documented then<br>**Total Score = +1**	1
- Psychosocial Assessment 2 recorded.	EPPSD score recorded	The Edinburgh Post Natal Depression Scale self-reported ten items documented then<br>**Total Score = +1.**	1
- ANRQ score documented	ANRQ	Where ANRQ value documented =1<br>**Total Score =+ 1 for Yes**<br>1=yes<br>2=No	1
5. *Antenatal Medication assessment:*	*ANM / medications string data only for separate coding for clinical outcomes*	*Comments: Documented prescribed and non prescribed medications or nil medications to be coded separately for clinical outcomes.*	NA
6. **Antenatal Cardiovascular Assessment** included:<br>BP<br>Pulse<br>12 Lead ECG<br>Cardiac Biomarkers<br>Echocardiogram<br>Yes<br>Not applicable<br>Unknown	CVSA	Where all components completed, and assessment done sum =6<br>**Total Score =+1**<br>1. Yes<br>2. BP<br>3. Pulse<br>4. 12 Lead ECG<br>5. Cardiac Biomarkers (clinical indication)<br>6. Echocardiogram<br>6. Not applicable<br>7. Unknown	1
7. *Cardiac Assessment comments*	*String data only for separate coding for clinical outcomes*	*Comments to be coded for clinical outcome*	NA
8. **Health Professional who performed Cardiac Assessment**	HP.	Where health professional included sum = 3, Midwife/ Obstetrician ± Physician, Cardiac Specialist Nurse, then<br>**Total Score =+1**	1

Supplementary 1 Code Log for Auditmaker and SAS scoring for adherence to the Statewide Perinatal Guidelines

Auditmaker Variable	SPSS Variable name / SAS code	Coding instructions /SAS Scoring for Obstetric/Cardiac Guideline adherence.	Adherence score
1= Physician / medical officer<br>2= Midwife<br>3= Cardiac Specialist Nurse<br>4= Obstetrician<br>5= Not applicable<br>6= Unknown		1= Physician /Medical officer<br>2= Midwife<br>3= Cardiac Specialist Nurse<br>4= Obstetrician<br>5= Not applicable<br>6= Unknown	
Health Professional who performed Cardiac Assessment comments	*String data only for separate coding for clinical outcomes*	*Comments to be coded for clinical outcome*	NA
9. **Antenatal management** for Hypertension<br>1= Not Identified<br>2= Identified and management optimised /reviewed<br>3= Identified and management not optimised /reviewed<br>4= Not Applicable<br>5= unknown	HT	When hypertension Identified & management optimised /reviewed<br>**Total Score =+1**<br><br>1= Not Identified<br>2= Identified and management optimised /reviewed<br>3= Identified and management not optimised /reviewed<br>4= Not Applicable<br>5= unknown	1
- *SBP = numerical value on admission*	*SBP*	*Numerical value to be coded separately for clinical outcomes*	NA
- *DBP = numerical value on admission*	*DBP*	*Numerical value to be coded separately for clinical outcomes*	NA
10. **Antenatal management** for Arrhythmias	Arrhythmia management	if Arrhythmia Identified & management optimised /reviewed<br>**Total score =+1**<br><br>1= Not Identified<br>2= Identified and management optimised /reviewed<br>3= Identified and management not optimised /reviewed<br>4= Not Applicable<br>5= No selection	1

Supplementary 1 Code Log for Auditmaker and SAS scoring for adherence to the Statewide Perinatal Guidelines

Auditmaker Variable	SPSS Variable name / SAS code	Coding instructions /SAS Scoring for Obstetric/Cardiac Guideline adherence.	Adherence score
11. **Antenatal management:** Lifestyle Factors Risk assessment total of 4<br>1= Smoking<br>2= Exercise Tolerance<br>3= Psychosocial Evaluation<br>4=Medical officer discussion re lifestyle with woman, family & cardiologist<br>5= Not Applicable	LSRA	Where the LSRA Sum= 4 then<br>**Total score= +1**<br>1= Smoking<br>2= Exercise Tolerance<br>3= Psychosocial Evaluation<br>4= Cardiologist & Medical officer discussed Lifestyle factors with woman, family & cardiologist<br>5= Not Applicable	1
12. **Preconception Cardiac Education**<br>1=Yes<br>2 =No<br>3= Not Applicable<br>4= Unknown	PreEdu	Where documented evidence of pre-education then<br>**Total Score =+ 1**<br>1=Yes<br>2 =No<br>3= Not Applicable<br>4= Unknown<br>Documented discussion before conception, which is applicable to pre-existent cardiac group A only. For all others leave as Not applicable	1
13. **Patient Informed re total of 9** Content :<br>**1.** Miscarriage<br>**2.** Live birth<br>**3.** Death<br>**4.** Antenatal Care<br>**5.** Intensive Care<br>**6.** Anaesthesia<br>**7.** Proposed care plan for pregnancy<br>**8.** Medications in the Peri-partum management<br>**9.** Genetic Counselling<br>0= Not applicable<br>01=Unknown	PreEdu2	Documented evidence of pre-education of potential outcomes minimum proposed plan for pregnancy and medications in peripartum management if (7,8) then **Total Score =+1**<br>1. Miscarriage<br>2. Live birth<br>3. Death<br>4. Antenatal Care<br>5. Intensive Care<br>6. Anaesthesia<br>7. Proposed care plan for pregnancy<br>8. Medications in the Peri-partum management<br>9. Genetic Counselling<br>10= Not applicable<br>01=Unknown	1

Auditmaker Variable	SPSS Variable name / SAS code	Coding instructions /SAS Scoring for Obstetric/Cardiac Guideline adherence.	Adherence score
14. **Ante-Natal Management offered**: total of 8<br>1. Routine Visits within 10-12 weeks<br>2. Early ultrasound to establish EDC<br>3. Documented Multi-Disciplinary Team management (Obstetrician, Cardiologist, HRP, anaesthetist, Intensivist)<br>4. If applicable: - fortnightly antenatal visits in High Risk Medical clinic asper the severity of the disease.<br>5. Not applicable – High risk antenatal visits<br>6. Avoid anaemia and regular Hb checks<br>7. Iron supplements + prenatal vitamins and dietary counselling<br>8. Anaesthetic review before 28 weeks<br>9.Unknown	ANM	Where antenatal management included addition of **(6,7)** then **Total Score =+1** Routine Visits within 10-12 weeks<br>1. Early ultrasound to establish EDC<br>2. Documented Multi-Disciplinary Team management (Obstetrician, Cardiologist, HRP, anaesthetist, Intensivist)<br>3. If applicable: - fortnightly antenatal visits in High Risk Medical clinic as per the severity of the disease.<br>4. Not applicable – High risk antenatal visits<br>5. Avoid anaemia and regular Hb checks<br>6. Iron supplements + prenatal vitamins and dietary counselling<br>7. Anaesthetic review before 28 weeks<br>9. Unknown	1
15. Maternal Age = numerical value	Maternalage	Numerical value	
16. Gestational Age on admission	Gestation	0-40+ weeks	
17. Gravida Numerical value for previous pregnancies	Gravida	Numerical value for previous pregnancies	
18. Parity : live babies from pregnancies	Parity	Numerical value for live babies from pregnancies	
19. **Antenatal management NYHFA documented**<br>1=yes<br>2=No<br>3= Unknown<br>4= Not applicable	NYHF	Where evidence NYHFA documented in case notes then **Total Score =+1 if documented: ,I II, III, IV**<br>1=Yes<br>2=No<br>3= Unknown<br>4= Not applicable	1
20. Comorbidities present<br>*1. Previous cardiac history*	*Comorbidities*	*String data with comments to be coded for clinical outcomes*<br>*1. Previous cardiac history*	

Auditmaker Variable	SPSS Variable name / SAS code	Coding instructions /SAS Scoring for Obstetric/Cardiac Guideline adherence.	Adherence score
2. Previous cardiac surgery<br>*3. Previous cardiac interventions*<br>*4. Previous cardiac lesions*<br>*5. Past/Current Cyanosis with saO2 <90%*<br>*6. Medication History*<br>*7. Use of Alcohol and Cigarettes*<br>*0=Not applicable*<br>*8=Unknown*		*2. Previous cardiac surgery*<br>*3. Previous cardiac interventions*<br>*4. Previous cardiac lesions*<br>*5. Past/Current Cyanosis with saO2 <90%*<br>*6. Medication History*<br>*7. Use of Alcohol and Cigarettes*<br>*8= Not applicable*<br>*9= Unknown*	
21. Previous cardiac history	*String data only for separate coding for clinical outcomes*	*Comments to be coded for clinical outcome*	
22. Previous cardiac surgery	*As above*	*Comments to be coded for clinical outcome*	
23. Previous cardiac interventions	*As above*	*Comments to be coded for clinical outcome*	
24. Previous cardiac lesions	*As above*	*Comments to be coded for clinical outcome*	
25. Past/Current Cyanosis with saO2 <90%	*As above*	*Comments to be coded for clinical outcome*	
26. Medication history	*As above*	*Comments to be coded for clinical outcome*	
27. Use of Alcohol and cigarettes	*As above*	*Comments to be coded for clinical outcome*	
28. 12 lead ECG findings	*As above*	*Comments to be coded for clinical outcome*	
29. Echocardiogram findings	*As above*	*Comments to be coded for clinical outcome*	
30. **Antenatal Management: Foetal Risk Assessment** 1.<br>3 components include;<br>1. Regular (Fortnightly)Ultrasound Assessment of fetal wellbeing<br>2. Growth, umbilical artery Doppler, AFI<br>3. Cardio topography (CTG) in the Third Trimester<br>4. Unknown<br>5. Not applicable.	FoetalRisk1	When documented evidence of 3 components sum= 3 then<br>**Total Score =+ 1**<br>1. Regular (Fortnightly)Ultrasound Assessment of fetal wellbeing<br>2. Growth, umbilical artery Doppler, AFI<br>3. Cardio topography (CTG) in the Third Trimester<br>4. Unknown<br>5. Not applicable	1
31. **Foetal Risk Assessment 2**.	FoetalRisk2	When documented evidence of two fetal risk assessment then<br>**Total Score = +1 for sum of 2**	1

Supplementary 1 Code Log for Auditmaker and SAS scoring for adherence to the Statewide Perinatal Guidelines

**Auditmaker Variable**	**SPSS Variable name / SAS code**	**Coding instructions /SAS Scoring for Obstetric/Cardiac Guideline adherence.**	**Adherence score**
1= Fetal Echocardiogram at 20 weeks of Gestation (if women on warfarin : identified cardiac foetal anomaly<br>2= Amniocentesis with antibiotic prophylaxis<br>3= Unknown		1= Fetal Echocardiogram at 20 weeks of Gestation (if women on warfarin : identified cardiac fetal anomaly)<br>2= Amniocentesis with antibiotic prophylaxis<br>3= Unknown	
32. **Antenatal management** / assessment Planned care 1. Cardiac Echocardiogram<br>1= yes<br>2= No<br>3= Unknown<br>4= Not applicable	Plannedcare1	When documented evidence that echocardiogram was done then **Total Score =+ 1 for yes**<br>1= yes<br>2= No<br>3= Unknown<br>4=Not applicable	1
33. Planned Care 2 Ongoing NYHA to assist with hospital choice<br>1= Yes<br>2= No<br>3= Unknown<br>4= Not applicable	Plannedcare2	When NYHA determined hospital choice for planned care then **Total Score = +1 for yes**<br>1= Yes<br>2= No<br>3= Unknown<br>4= Not applicable	1
34. Planned Care: Level of Hospital Care comments	*String data only for separate coding for clinical outcomes*	*Comments to be coded for clinical outcome*	NA
35. Planned Care 3. Mode of delivery<br>1= NVD<br>2= Emergency LSCS<br>3= LSCS for other<br>4= Nil documented	Plannedcare 3	To be coded for clinical outcomes<br>1= NVD<br>2= Emergency LSCS<br>3= LSCS for other<br>4 = Nil documented	
36. Planned delivery mode comments	*Normal vaginal delivery/ assisted vaginal delivery / elective LSCS and non elective LSCS*	*Comments to be coded for clinical outcome*	

Supplementary 1 Code Log for Auditmaker and SAS scoring for adherence to the Statewide Perinatal Guidelines

Auditmaker Variable	SPSS Variable name / SAS code	Coding instructions /SAS Scoring for Obstetric/Cardiac Guideline adherence.	Adherence score
37. **High Risk Management During Labor 1** Identified as high Risk<br>1= Yes<br>2= No<br>3= Not applicable<br>4= Unknown	Highrisk1	When identified and documented as high risk then<br>**Total Score =+1 for yes**<br>1= Yes<br>2= No<br>3= Not applicable<br>4= Unknown	1
38. **High Risk Management During Labour 2**<br>Labour and Birth occurred in a hospital with Adult Intensive Care or Cardiac Unit<br>1= Yes<br>2= No<br>3= Unknown<br>4= Not applicable	Highrisk2	High Risk Management in Labour 2 then<br>**Total Score =+ 1 for yes**<br>Labour + Birth in ICU or CCU occurred in tertiary/ quaternary hospitals<br>1= Yes<br>2= No<br>3= Unknown<br>4= Not applicable	1
39. **High Risk Management During labour 3**<br>Documented Plan of Care in case notes<br>1= yes<br>2= No<br>3= Unknown<br>4= Not applicable	Highrisk3	When documented evidence of Plan of Care in case notes then<br>**Total Score =+1 for Yes.**<br>1= Yes<br>2= No<br>3= Unknown<br>4= Not applicable	1
40. **High Risk Management of labour** 4: Timing & labour of birth included the components of 1-3<br>Content<br>1. Multidisciplinary team Discussion<br>2. Cardiac state well stabilised	Highrisk4	Where the timing and labour of birth included 1-3 then<br>**Total Score =+1.**<br><br>1. Multidisciplinary team Discussion.<br>2. Cardiac state well stabilised<br>3. At short notice in the event of cardiac function and reserve altered<br>4. Unknown<br>5. Not applicable	1

Supplementary 1 Code Log for Auditmaker and SAS scoring for adherence to the Statewide Perinatal Guidelines

Auditmaker Variable	SPSS Variable name / SAS code	Coding instructions /SAS Scoring for Obstetric/Cardiac Guideline adherence.	Adherence score
3. At short notice in the event of altered cardiac function & reserve<br>4. Unknown<br>5. Not applicable			
41. **High Risk Management of labour** 5. The multidisciplinary team informed/ documentation of impending delivery: included 1-3<br>1. Anaesthetist<br>2. Cardiologist<br>3. Paediatrician<br>4. Unknown<br>5. Not applicable	Highrisk5	Multidisciplinary team communication for impending delivery then **Total Score =+1 for the sum of 3**<br>1. Anaesthetist<br>2. Cardiologist<br>3. Paediatrician<br>4. Unknown<br>5. Not applicable	1
42. **Management of labour** Paediatrician included in discussion e.g. impending delivery (IUGR & SFD)	Highrisk5.(3) Paediatrician included	Where explicit inclusion of paeditrician delivery then **Total Score =+1**	1
43. **Management of labour** Antibiotic Prophylaxis administered<br>1= Yes<br>2 = No<br>3= Not applicable<br>4= Unknown	Highrisk6	When prophylactic antibiotics administered then **Total Score =+1 for Yes**<br>1=Yes<br>2 = No<br>3= Unknown<br>4= Not applicable	1
44. **Management of labour** Planned Cardiac drugs in labour	Highrisk7	When cardiac drugs planned for use in labour with MDT discussion (documented) then **Total Score =+1 for Yes**<br>1=Yes	1

**Auditmaker Variable**	**SPSS Variable name / SAS code**	**Coding instructions /SAS Scoring for Obstetric/Cardiac Guideline adherence.**	**Adherence score**
Collaboration with Multidisciplinary Team<br>1=Yes<br>2 = No<br>3= Unknown<br>4= Not applicable		2 = No<br>3= Unknown<br>4= Not applicable	
45. *High Risk labour comments*	*String data only for separate coding for clinical outcomes*	*Comments to be coded for clinical outcome*	NA
46. **Management of labour** The second stage of delivery is shortened to avoid excessive maternal expulsive effort<br>1= Yes, with Episiotomy<br>2 = Yes, With Forceps Delivery<br>3 = Yes, with local anaesthetics + adrenaline use<br>4= No<br>5 = Unknown<br>6 = Not applicable.	Highrisk8	When the 2nd stage shortened to avoid excessive maternal expulsive effort with following<br>1= Yes, with Episiotomy<br>2 = Yes, With Forceps Delivery<br>3 = Yes, local anaesthetics + adrenaline use<br>4= No<br>5 = Unknown<br>6 = Not applicable	
47. **Management of labour** with mminimization of Cardiac compromise in labour score out of 4<br>1. Consultation re avoiding the use of stirrups<br>2. Assessment + prompt reporting of Tachycardia >130bpm<br>3. Assessment + prompt reporting of Bradycardia < 40bpm	Highrisk 9 Compromise	Where sum of measures to minimize cardiac compromise in labour=4 then **Total Score =+ 1**<br>1. Consultation re avoiding the use of stirrups<br>2. Assessment + prompt reporting of Tachycardia >130bpm<br>3. Assessment + prompt reporting of Bradycardia < 40bpm<br>4. Assessment + prompt reporting of respiratory rate >24 or < 5 bpm<br>5. Assessment+ prompt reporting rapidly changing pulse or respiration rate (even if within threshold)<br>6. Unknown<br>7. Not applicable	1

Supplementary 1 Code Log for Auditmaker and SAS scoring for adherence to the Statewide Perinatal Guidelines

**Auditmaker Variable**	**SPSS Variable name / SAS code**	**Coding instructions /SAS Scoring for Obstetric/Cardiac Guideline adherence.**	**Adherence score**
4. Assessment + prompt reporting of respiratory rate >24 or < 5 bpm<br>5. Assessment+ prompt reporting rapidly changing pulse or respiration rate (even if within the threshold)<br>6. Unknown<br>7. Not applicable			
48. *Birth comments*	*String data only for separate coding for clinical outcomes*	*Comments to be coded for clinical outcome*	NA
49. **Management in labour**: Adult resuscitation equipment available?	Management in Labour resuscitation equip	Where adult resuscitation equipment (note for WAS clinic etc.) available then **Total Score =+1 for Yes**<br>1=Yes<br>2= No<br>3 = Null	1
50. **Management in labour**: Ability to perform peri mortem Caesarean section within 5 minutes of cardiac arrest	Labour2/ Management in Labour 5 mins cardiac	Equipment & staff for emergency perimortem LSCS available then **Total Score =+1 for Yes** 1=Yes<br>2= No<br>3 = Null	1
51. **Management in labour** Multidisciplinary team included.	Team in labour/<br>Team 1 Obstetrician<br>Team 5 Anaesthetist<br>Team 4 Physician (High Risk/ medical officer).<br><br>Team sum where includes 5, 67	If yes for Obstetrician**, SAS score=Total Score+1**<br>if yes for Anaesthetist**, SAS score=Total Score+1**<br>If yes for Physician(medical officer) **SAS score=Total Score+1**<br>When MDT in labour included team sum includes *anaethatist , intensivist, & allied health professional (5,6,7)* then **SAS score=Total Score +1**<br><br>1. Obstetrician<br>2. Midwife<br>3. Cardiologist<br>4. Medical officer	1<br>1<br>1<br>1

Auditmaker Variable	SPSS Variable name / SAS code	Coding instructions /SAS Scoring for Obstetric/Cardiac Guideline adherence.	Adherence score
		5. Anaesthetist<br>6. Intensivist<br>7. Other Allied health professionals<br>8. Other<br>9. Unknown	
52. **Management in Labour** included cardiac consultation	Cardiac Consultation	Where cardiac consultation occurred for management in labour then **Total Score =+1 for Yes.**	1
53. Management in labour comments	*String data only for separate coding for clinical outcomes*	*Comments to be coded for clinical outcome*	NA
54. **Management in labour**: Thromboembolism prophylaxis	Thromboembolism sum of 2	**If the sum of 2 Total score =+ 1**<br>1= Use of anti- embolic stockings<br>2= Subcutaneous LMWH or IV Unfractionated heparin<br>3= Not applicable<br>4= Unknown	1
55. **Pain Management & Anaesthesia**<br>• Anaesthetic consultation regarding appropriate pain management undertaken<br>• Epidural considered<br>• Combined spinal –epidural to decrease preload and afterload<br>• Not applicable<br>• Other	Pain sum where included anaesthetic consultation and epidural combined spinal	Pain management sum where included anaesthetic consultation, epidural and /or combined spinal **Total score =+1**<br>1. Anaesthetic consultation regarding appropriate pain management undertaken<br>2. Epidural considered<br>3. Combined spinal –epidural to decrease preload and afterload<br>4. Not applicable<br>5. Other	1
56. Pain Management and Anaesthesia comments	*String data only for separate coding for clinical outcomes*	*Comments to be coded for clinical outcome*	NA
57. **Management in labour** Cardiac collaboration /consultation with cardiologist /physician advice sought	Labour5/If Cardiac Consultation	Where documented cardiac consultation for labour then **Total Score =+1**<br>1=Yes<br>2= No<br>3 = Not applicable	1

Supplementary 1 Code Log for Auditmaker and SAS scoring for adherence to the Statewide Perinatal Guidelines

Auditmaker Variable	SPSS Variable name / SAS code	Coding instructions /SAS Scoring for Obstetric/Cardiac Guideline adherence.	Adherence score
		4=Unknown<br>5=null	
58. **Management in labour** Paediatric and neonatal staff available.	Labour6 /paediatric neonatal staff present	When paediatric neonatal staff present at delivery then<br>**Total Score =+1**<br>1=Yes<br>2= No<br>3 = Not applicable<br>4=Unknown<br>5=No selection	1
59. Outcome of the birth	Outcomebirth	Alive & Well,	
60. Gestational Age	Gestationalage	Numeric	
61. Live baby weight	Newbornweight	Numeric	
62. Baby's length at birth	Newbornlength	Numeric	
63. Baby's Head circumference at birth	NewbornHC	Numeric	
64. Apgar score at 1 min	Apgarscore	Numeric	
65. Apgar at 5 min	Apgascore1	Numeric	
66. Cardiac follow-up included in obstetric follow-up care	*String data only for separate coding for clinical outcomes*	*Comments to be coded for clinical outcome*	
**Total Adherence score**			**40**

∗Please note that string data have not been included in the current code book as will be analysed for clinical outcomes. This tables provides an overview of the data collected and adherence scores calculation. The Auditmaker variables from audit tool reflect the South Australian Perinatal Practice Guidelines (SAPPGs). ∗∗Hospital sites tertiary, quaternary and Maternity Standalone unit as per SA Health: **Hospital 1** provided intermediate care (level five) that encompassed tertiary maternal services, maternal cardiac and intensive care and specialised neonatal care services excluding babies less than 32 weeks. **Hospital 2** provided (level six) care that included maternal cardiac, cardiothoracic surgical and intensive care, and neonatal intensive care services. **Hospital 3** provided a neonatal intensive care unit but no maternal cardiac or intensive care services.
