# Supplementary material for: Adherence to clinical practice guidelines for South Australian pregnant women with cardiac conditions between 2003 and 2013
Source: PLoS One. 2020 Mar 17;15(3):e0230459. doi: 10.1371/journal.pone.0230459 (PMC7077829; doi:10.1371/journal.pone.0230459)
Supplement: S2 Table — (PDF) [file pone.0230459.s002.pdf]

Model 1 Final Multivariable Logistic regression of Preconception versus Group, site and confounders

The GENMOD Procedure

Model Information	
Data Set	WORK.CARDIAC2
Distribution	Binomial
Link Function	Logit
Dependent Variable	Preconception

Number of Observations Read	271
Number of Observations Used	57
Number of Events	38
Number of Trials	57
Missing Values	214

Class Level Information

Class	*Levels*	*Values*
Revised_Groups	2	Acquired Pre-existent
Site	3	1 2 3
Cardiac_consultation	2	1.Yes 2.No

Response Profile

Ordered Value	*Preconception*	*Total Frequency*
1	Yes	38
2	No	19

PROC GENMOD is modeling the probability that Preconception='Yes'.

Parameter Information				
Parameter	*Effect*	*Revised_Groups*	*Site*	*Cardiac_consultation*
Prm1	Intercept			
Prm2	Revised_Groups	Acquired		
Prm3	Revised_Groups	Pre-existent		
Prm4	Site		1	
Prm5	Site		2	
Prm6	Site		3	
Prm7	Cardiac_consultation			1.Yes
Prm8	Cardiac_consultation			2.No
Prm9	Gravida			

Criteria For Assessing Goodness Of Fit			
Criterion	*DF*	*Value*	*Value/DF*
Log Likelihood		-29.3365	
Full Log Likelihood		-29.3365	
AIC (smaller is better)		70.6731	
AICC (smaller is better)		72.3531	
BIC (smaller is better)		82.9314	

Algorithm converged.

Analysis Of Maximum Likelihood Parameter Estimates								
Parameter		*DF*	*Estimate*	*Standard Error*	*Wald 95% Confidence Limits*		*Wald Chi-Square*	*Pr > ChiSq*
Intercept		1	-2.7521	1.3674	-5.4322	-0.0720	4.05	0.0442
Revised_Groups	Acquired	1	-0.8460	0.7327	-2.2821	0.5900	1.33	0.2482
Revised_Groups	Pre-existant	0	0.0000	0.0000	0.0000	0.0000	.	.
Site	1	1	-2.4282	1.5453	-5.4570	0.6005	2.47	0.1161

Analysis Of Maximum Likelihood Parameter Estimates									
Parameter		DF	Estimate	Standard Error	Wald 95% Confidence Limits		Wald Chi-Square	Pr > ChiSq
Site	2	1	-0.2579	0.6876	-1.6056	1.0897	0.14	0.7076
Site	3	0	0.0000	0.0000	0.0000	0.0000	.	.
Cardiac_consultation	1.Yes	1	2.9187	1.1276	0.7086	5.1288	6.70	0.0096
Cardiac_consultation	2.No	0	0.0000	0.0000	0.0000	0.0000	.	.
Gravida		1	0.4757	0.2285	0.0279	0.9235	4.34	0.0373
Scale		0	1.0000	0.0000	1.0000	1.0000		

Note: The scale parameter was held fixed.

Wald Statistics For Type 3 Analysis

Source	DF	Chi-Square	Pr > ChiSq
Revised_Groups	1	1.33	0.2482
Site	2	2.48	0.2895
Cardiac_consultation	1	6.70	0.0096
Gravida	1	4.34	0.0373

Contrast Estimate Results

Label	Mean Estimate	Mean Confidence Limits		L'Beta Estimate	Standard Error	Alpha	L'Beta Confidence Limits		Chi-Square	Pr > ChiSq
Gravida	0.6167	0.5070	0.7157	0.4757	0.2285	0.05	0.0279	0.9235	4.34	0.0373
Exp(Gravida)				1.6091	0.3676	0.05	1.0283	2.5180		

Revised_Groups Least Squares Means

Revised_Groups	Estimate	Standard Error	z Value	Pr > |z|	Alpha	Lower	Upper	Exponentiated	Exponentiated Lower	Exponentiated Upper
Acquired	-1.4985	0.9622	-1.56	0.1194	0.05	-3.3845	0.3874	0.2235	0.03389	1.4731

Revised_Groups Least Squares Means

Revised_Groups	*Estimate*	*Standard Error*	*z Value*	*Pr > |z|*	*Alpha*	*Lower*	*Upper*	*Exponentiated*	*Exponentiated Lower*	*Exponentiated Upper*
Pre-existant	-0.6525	0.6731	-0.97	0.3323	0.05	-1.9717	0.6667	0.5207	0.1392	1.9479

Differences of Revised_Groups Least Squares Means

Revised_Groups	*_Revised_Groups*	*Estimate*	*Standard Error*	*z Value*	*Pr > |z|*	*Alpha*	*Lower*	*Upper*	*Exponentiated*	*Exponentiated Lower*	*Exponentiated Upper*
Acquired	Pre-existant	-0.8460	0.7327	-1.15	0.2482	0.05	-2.2821	0.5900	0.4291	0.1021	1.8041

Site Least Squares Means

Site	*Estimate*	*Standard Error*	*z Value*	*Pr > |z|*	*Alpha*	*Lower*	*Upper*	*Exponentiated*	*Exponentiated Lower*	*Exponentiated Upper*
1	-2.6084	1.5875	-1.64	0.1004	0.05	-5.7199	0.5031	0.07365	0.003280	1.6539
2	-0.4381	0.6749	-0.65	0.5163	0.05	-1.7608	0.8846	0.6453	0.1719	2.4221
3	-0.1801	0.6206	-0.29	0.7716	0.05	-1.3964	1.0361	0.8352	0.2475	2.8183

Differences of Site Least Squares Means

Site	*_Site*	*Estimate*	*Standard Error*	*z Value*	*Pr > |z|*	*Alpha*	*Lower*	*Upper*	*Exponentiated*	*Exponentiated Lower*	*Exponentiated Upper*
1	2	-2.1703	1.4914	-1.46	0.1456	0.05	-5.0934	0.7528	0.1141	0.006137	2.1229
1	3	-2.4282	1.5453	-1.57	0.1161	0.05	-5.4570	0.6005	0.08819	0.004266	1.8231
2	3	-0.2579	0.6876	-0.38	0.7076	0.05	-1.6056	1.0897	0.7727	0.2008	2.9734

Cardiac_consultation Least Squares Means

Cardiac_consultationx	*Estimate*	*Standard Error*	*z Value*	*Pr > |z|*	*Alpha*	*Lower*	*Upper*	*Exponentiated*	*Exponentiated Lower*	*Exponentiated Upper*
1.Yes	0.3838	0.5554	0.69	0.4895	0.05	-0.7047	1.4724	1.4679	0.4942	4.3597
2.No	-2.5349	1.1991	-2.11	0.0345	0.05	-4.8851	-0.1847	0.07927	0.007559	0.8313

Differences of Cardiac_consultation Least Squares Means												
Cardiac_consultationx	_Cardiac_consultationx	Estimate	Standard Error	z Value	Pr > |z|	Alpha	Lower	Upper	Exponentiated	Exponentiated Lower	Exponentiated Upper
1.Yes	2.No	2.9187	1.1276	2.59	0.0096	0.05	0.7086	5.1288	18.5177	2.0312	168.82

Model 17 Final Multivariable Ordinal logistic regression of ANC1 versus Group, site and confounders

The GENMOD Procedure

Model Information	
Data Set	WORK.CARDIAC2
Distribution	Multinomial
Link Function	Cumulative Logit
Dependent Variable	ANC1

Number of Observations Read	271
Number of Observations Used	259
Missing Values	12

Class Level Information		
Class	Levels	Values
Revised_Groups	2	Acquired Pre-existant
Site	3	1 2 3
ANRQ_scoreRECORDED	2	1.Yes 2.No

Response Profile		
Ordered Value	ANC1	Total Frequency
1	3	172
2	2	32

Response Profile		
Ordered Value	ANC1	Total Frequency
3	1	25
4	0	30

PROC GENMOD is modeling the probabilities of levels of ANC1 having LOWER Ordered Values in the response profile table.

Parameter Information				
Parameter	Effect	Revised_Groups	Site	ANRQ_scoreRECORDED
Prm1	Revised_Groups	Acquired		
Prm2	Revised_Groups	Pre-existant		
Prm3	Site		1	
Prm4	Site		2	
Prm5	Site		3	
Prm6	ANRQ_scoreRECORDED			1.Yes
Prm7	ANRQ_scoreRECORDED			2.No
Prm8	Gestational_age_admi			

Criteria For Assessing Goodness Of Fit			
Criterion	DF	Value	Value/DF
Log Likelihood		-213.5324	
Full Log Likelihood		-213.5324	
AIC (smaller is better)		443.0649	
AICC (smaller is better)		443.6409	
BIC (smaller is better)		471.5195	

Algorithm converged.

Analysis Of Maximum Likelihood Parameter Estimates								
Parameter		*DF*	*Estimate*	*Standard Error*	*Wald 95% Confidence Limits*		*Wald Chi-Square*	*Pr > ChiSq*
Intercept1		1	-3.6725	1.3371	-6.2931	-1.0519	7.54	0.0060
Intercept2		1	-2.8125	1.3295	-5.4183	-0.2067	4.48	0.0344
Intercept3		1	-1.9564	1.3231	-4.5496	0.6368	2.19	0.1392
Revised_Groups	Acquired	1	0.0011	0.2963	-0.5797	0.5818	0.00	0.9972
Revised_Groups	Pre-existant	0	0.0000	0.0000	0.0000	0.0000	.	.
Site	1	1	-0.2097	0.5327	-1.2537	0.8344	0.15	0.6939
Site	2	1	1.5216	0.3694	0.7976	2.2457	16.97	<.0001
Site	3	0	0.0000	0.0000	0.0000	0.0000	.	.
ANRQ_scoreRECORDED	1.Yes	1	2.4254	0.4489	1.5455	3.3052	29.19	<.0001
ANRQ_scoreRECORDED	2.No	0	0.0000	0.0000	0.0000	0.0000	.	.
Gestational_age_admi		1	0.0850	0.0351	0.0163	0.1537	5.88	0.0153
Scale		0	1.0000	0.0000	1.0000	1.0000		

Note: The scale parameter was held fixed.

Wald Statistics For Type 3 Analysis			
Source	*DF*	*Chi-Square*	*Pr > ChiSq*
Revised_Groups	1	0.00	0.9972
Site	2	18.52	<.0001
ANRQ_scoreRECORDED	1	29.19	<.0001
Gestational_age_admi	1	5.88	0.0153

Contrast Estimate Results										
Label	*Mean Estimate*	*Mean Confidence Limits*		*L'Beta Estimate*	*Standard Error*	*Alpha*	*L'Beta Confidence Limits*		*Chi-Square*	*Pr > ChiSq*
Gestational_age_admission	0.5212	0.5041	0.5384	0.0850	0.0351	0.05	0.0163	0.1537	5.88	0.0153

Contrast Estimate Results

Label	Mean Estimate	Mean Confidence Limits		L'Beta Estimate	Standard Error	Alpha	L'Beta Confidence Limits		Chi-Square	Pr > ChiSq
Exp(Gestational_age_admission)				1.0887	0.0382	0.05	1.0164	1.1662		

Revised_Groups Least Squares Means

ANC1	Revised_Groups	Estimate	Standard Error	z Value	Pr > |z|	Alpha	Lower	Upper	Exponentiated	Exponentiated Lower	Exponentiated Upper
3	Acquired	1.1249	0.2651	4.24	<.0001	0.05	0.6053	1.6446	3.0801	1.8318	5.1788
3	Pre-existant	1.1239	0.2662	4.22	<.0001	0.05	0.6022	1.6456	3.0768	1.8261	5.1843
2	Acquired	1.9849	0.2870	6.92	<.0001	0.05	1.4225	2.5474	7.2787	4.1473	12.7743
2	Pre-existant	1.9839	0.2865	6.92	<.0001	0.05	1.4224	2.5454	7.2710	4.1470	12.7483
1	Acquired	2.8410	0.3159	8.99	<.0001	0.05	2.2218	3.4603	17.1333	9.2237	31.8254
1	Pre-existant	2.8400	0.3184	8.92	<.0001	0.05	2.2159	3.4640	17.1152	9.1696	31.9459

Differences of Revised_Groups Least Squares Means

Revised_Groups	_Revised_Groups	Estimate	Standard Error	z Value	Pr > |z|	Alpha	Lower	Upper	Exponentiated	Exponentiated Lower	Exponentiated Upper
Acquired	Pre-existant	0.001053	0.2963	0.00	0.9972	0.05	-0.5797	0.5818	1.0011	0.5600	1.7893

Site Least Squares Means

ANC1	Site	Estimate	Standard Error	z Value	Pr > |z|	Alpha	Lower	Upper	Exponentiated	Exponentiated Lower	Exponentiated Upper
3	1	0.4774	0.4621	1.03	0.3015	0.05	-0.4283	1.3831	1.6119	0.6516	3.9874
3	2	2.2087	0.3403	6.49	<.0001	0.05	1.5417	2.8757	9.1042	4.6727	17.7385
3	3	0.6871	0.2651	2.59	0.0095	0.05	0.1676	1.2066	1.9879	1.1825	3.3420
2	1	1.3374	0.4724	2.83	0.0046	0.05	0.4116	2.2632	3.8093	1.5093	9.6142
2	2	3.0687	0.3682	8.33	<.0001	0.05	2.3471	3.7903	21.5147	10.4555	44.2717
2	3	1.5471	0.2754	5.62	<.0001	0.05	1.0073	2.0869	4.6978	2.7381	8.0599
1	1	2.1935	0.4887	4.49	<.0001	0.05	1.2356	3.1514	8.9666	3.4406	23.3684

Site Least Squares Means											
ANC1	*Site*	*Estimate*	*Standard Error*	*z Value*	*Pr > |z|*	*Alpha*	*Lower*	*Upper*	*Exponentiated*	*Exponentiated Lower*	*Exponentiated Upper*
1	2	3.9248	0.3979	9.86	<.0001	0.05	3.1449	4.7048	50.6436	23.2167	110.47
1	3	2.4032	0.3041	7.90	<.0001	0.05	1.8071	2.9992	11.0581	6.0928	20.0699

Differences of Site Least Squares Means											
Site	*_Site*	*Estimate*	*Standard Error*	*z Value*	*Pr > |z|*	*Alpha*	*Lower*	*Upper*	*Exponentiated*	*Exponentiated Lower*	*Exponentiated Upper*
1	2	-1.7313	0.5839	-2.96	0.0030	0.05	-2.8758	-0.5868	0.1771	0.05637	0.5561
1	3	-0.2097	0.5327	-0.39	0.6939	0.05	-1.2537	0.8344	0.8109	0.2854	2.3034
2	3	1.5216	0.3694	4.12	<.0001	0.05	0.7976	2.2457	4.5798	2.2202	9.4470

ANRQ_scoreRECORDED Least Squares Means											
ANC1	*ANRQ_scoreRECORDED*	*Estimate*	*Standard Error*	*z Value*	*Pr > |z|*	*Alpha*	*Lower*	*Upper*	*Exponentiated*	*Exponentiated Lower*	*Exponentiated Upper*
3	1.Yes	2.3371	0.3779	6.18	<.0001	0.05	1.5964	3.0779	10.3513	4.9350	21.7121
3	2.No	-0.08827	0.2349	-0.38	0.7071	0.05	-0.5486	0.3721	0.9155	0.5778	1.4507
2	1.Yes	3.1971	0.4023	7.95	<.0001	0.05	2.4087	3.9855	24.4617	11.1192	53.8145
2	2.No	0.7717	0.2439	3.16	0.0016	0.05	0.2938	1.2497	2.1635	1.3415	3.4893
1	1.Yes	4.0532	0.4291	9.45	<.0001	0.05	3.2122	4.8942	57.5805	24.8328	133.51
1	2.No	1.6278	0.2719	5.99	<.0001	0.05	1.0949	2.1607	5.0927	2.9888	8.6775

Differences of ANRQ_scoreRECORDED Least Squares Means											
ANRQ_scoreRECORDED	*_ANRQ_scoreRECORDED*	*Estimate*	*Standard Error*	*z Value*	*Pr > |z|*	*Alpha*	*Lower*	*Upper*	*Exponentiated*	*Exponentiated Lower*	*Exponentiated Upper*
1.Yes	2.No	2.4254	0.4489	5.40	<.0001	0.05	1.5455	3.3052	11.3065	4.6905	27.2542

Model 18 Final Multivariable Ordinal logistic regression of ANC2 versus Group, site and confounders

The GENMOD Procedure

Model Information	
Data Set	WORK.CARDIAC2
Distribution	Multinomial
Link Function	Cumulative Logit
Dependent Variable	ANC2

Number of Observations Read	271
Number of Observations Used	64
Missing Values	207

Class Level Information		
Class	*Levels*	*Values*
Revised_Groups	2	Acquired Pre-existant
Site	3	1 2 3
Highrisk1	2	1.Yes 2.No

Response Profile		
Ordered Value	*ANC2*	*Total Frequency*
1	3	12
2	2	30
3	1	15
4	0	7

PROC GENMOD is modeling the probabilities of levels of ANC2 having LOWER Ordered Values in the response profile table.

Parameter Information				
Parameter	*Effect*	*Revised_Groups*	*Site*	*Highrisk1*
Prm1	Revised_Groups	Acquired		

Parameter Information				
Parameter	*Effect*	*Revised_Groups*	*Site*	*Highrisk1*
Prm2	Revised_Groups	Pre-existant		
Prm3	Site		1	
Prm4	Site		2	
Prm5	Site		3	
Prm6	Highrisk1			1.Yes
Prm7	Highrisk1			2.No
Prm8	Babys_length_birth			
Prm9	ANRQ_score			

Criteria For Assessing Goodness Of Fit			
Criterion	*DF*	*Value*	*Value/DF*
Log Likelihood		-65.6960	
Full Log Likelihood		-65.6960	
AIC (smaller is better)		149.3919	
AICC (smaller is better)		152.7253	
BIC (smaller is better)		168.8219	

Algorithm converged.

Analysis Of Maximum Likelihood Parameter Estimates								
Parameter		*DF*	*Estimate*	*Standard Error*	*Wald 95% Confidence Limits*		*Wald Chi-Square*	*Pr > ChiSq*
Intercept1		1	4.6504	2.6541	-0.5515	9.8524	3.07	0.0797
Intercept2		1	7.5676	2.7838	2.1115	13.0238	7.39	0.0066
Intercept3		1	9.5519	2.8689	3.9290	15.1749	11.09	0.0009
Revised_Groups	Acquired	1	0.6688	0.5324	-0.3747	1.7123	1.58	0.2091
Revised_Groups	Pre-existant	0	0.0000	0.0000	0.0000	0.0000	.	.
Site	1	1	-0.7845	0.6738	-2.1051	0.5361	1.36	0.2443

Analysis Of Maximum Likelihood Parameter Estimates								
Parameter		DF	Estimate	Standard Error	Wald 95% Confidence Limits		Wald Chi-Square	Pr > ChiSq
Site	2	1	-1.3899	0.6625	-2.6884	-0.0914	4.40	0.0359
Site	3	0	0.0000	0.0000	0.0000	0.0000	.	.
Highrisk1	1.Yes	1	1.2366	0.5590	0.1411	2.3322	4.89	0.0269
Highrisk1	2.No	0	0.0000	0.0000	0.0000	0.0000	.	.
Babys_length_birth		1	-0.1201	0.0544	-0.2268	-0.0134	4.87	0.0273
ANRQ_score		1	-0.0656	0.0205	-0.1058	-0.0255	10.26	0.0014
Scale		0	1.0000	0.0000	1.0000	1.0000		

Note: The scale parameter was held fixed.

Wald Statistics For Type 3 Analysis			
Source	DF	Chi-Square	Pr > ChiSq
Revised_Groups	1	1.58	0.2091
Site	2	4.43	0.1092
Highrisk1	1	4.89	0.0269
Babys_length_birth	1	4.87	0.0273
ANRQ_score	1	10.26	0.0014

Contrast Estimate Results										
		Mean					L'Beta			
Label	Mean Estimate	Confidence Limits		L'Beta Estimate	Standard Error	Alpha	Confidence Limits		Chi-Square	Pr > ChiSq
Babys_length_birth	0.4700	0.4435	0.4966	-0.1201	0.0544	0.05	-0.2268	-0.0134	4.87	0.0273
Exp(Babys_length_birth)				0.8868	0.0483	0.05	0.7971	0.9867		
ANRQ_score	0.4836	0.4736	0.4936	-0.0656	0.0205	0.05	-0.1058	-0.0255	10.26	0.0014
Exp(ANRQ_score)				0.9365	0.0192	0.05	0.8996	0.9748		

Revised_Groups Least Squares Means

ANC2	*Revised_Groups*	*Estimate*	*Standard Error*	*z Value*	*Pr > |z|*	*Alpha*	*Lower*	*Upper*	*Exponentiated*	*Exponentiated Lower*	*Exponentiated Upper*
3	Acquired	-1.8237	0.4440	-4.11	<.0001	0.05	-2.6940	-0.9534	0.1614	0.06761	0.3854
3	Pre-existant	-2.4925	0.5312	-4.69	<.0001	0.05	-3.5336	-1.4515	0.08270	0.02920	0.2342
2	Acquired	1.0934	0.4083	2.68	0.0074	0.05	0.2933	1.8936	2.9845	1.3408	6.6435
2	Pre-existant	0.4247	0.4546	0.93	0.3502	0.05	-0.4663	1.3156	1.5291	0.6273	3.7271
1	Acquired	3.0778	0.5692	5.41	<.0001	0.05	1.9622	4.1934	21.7098	7.1146	66.2464
1	Pre-existant	2.4090	0.5519	4.36	<.0001	0.05	1.3272	3.4907	11.1227	3.7706	32.8101

Differences of Revised_Groups Least Squares Means

Revised_Groups	*_Revised_Groups*	*Estimate*	*Standard Error*	*z Value*	*Pr > |z|*	*Alpha*	*Lower*	*Upper*	*Exponentiated*	*Exponentiated Lower*	*Exponentiated Upper*
Acquired	Pre-existant	0.6688	0.5324	1.26	0.2091	0.05	-0.3747	1.7123	1.9519	0.6875	5.5415

Site Least Squares Means

ANC2	*Site*	*Estimate*	*Standard Error*	*z Value*	*Pr > |z|*	*Alpha*	*Lower*	*Upper*	*Exponentiated*	*Exponentiated Lower*	*Exponentiated Upper*
3	1	-2.2178	0.5451	-4.07	<.0001	0.05	-3.2861	-1.1495	0.1088	0.03740	0.3168
3	2	-2.8232	0.5552	-5.09	<.0001	0.05	-3.9114	-1.7351	0.05941	0.02001	0.1764
3	3	-1.4333	0.5590	-2.56	0.0103	0.05	-2.5289	-0.3377	0.2385	0.07975	0.7134
2	1	0.6994	0.4716	1.48	0.1380	0.05	-0.2249	1.6236	2.0125	0.7986	5.0713
2	2	0.09394	0.4226	0.22	0.8241	0.05	-0.7344	0.9223	1.0985	0.4798	2.5150
2	3	1.4839	0.5982	2.48	0.0131	0.05	0.3114	2.6563	4.4100	1.3654	14.2432
1	1	2.6837	0.6062	4.43	<.0001	0.05	1.4955	3.8719	14.6390	4.4614	48.0342
1	2	2.0783	0.5190	4.00	<.0001	0.05	1.0610	3.0956	7.9905	2.8891	22.0996
1	3	3.4682	0.7105	4.88	<.0001	0.05	2.0757	4.8607	32.0785	7.9698	129.12

Differences of Site Least Squares Means											
Site	*_Site*	*Estimate*	*Standard Error*	*z Value*	*Pr > |z|*	*Alpha*	*Lower*	*Upper*	*Exponentiated*	*Exponentiated Lower*	*Exponentiated Upper*
1	2	0.6054	0.5842	1.04	0.3000	0.05	-0.5396	1.7504	1.8320	0.5830	5.7572
1	3	-0.7845	0.6738	-1.16	0.2443	0.05	-2.1051	0.5361	0.4563	0.1218	1.7094
2	3	-1.3899	0.6625	-2.10	0.0359	0.05	-2.6884	-0.09145	0.2491	0.06799	0.9126

Highrisk1 Least Squares Means											
ANC2	*Highrisk1*	*Estimate*	*Standard Error*	*z Value*	*Pr > |z|*	*Alpha*	*Lower*	*Upper*	*Exponentiated*	*Exponentiated Lower*	*Exponentiated Upper*
3	1.Yes	-1.5398	0.3947	-3.90	<.0001	0.05	-2.3134	-0.7662	0.2144	0.09893	0.4648
3	2.No	-2.7764	0.5814	-4.78	<.0001	0.05	-3.9160	-1.6369	0.06226	0.01992	0.1946
2	1.Yes	1.3774	0.4037	3.41	0.0006	0.05	0.5861	2.1686	3.9645	1.7970	8.7460
2	2.No	0.1407	0.4742	0.30	0.7666	0.05	-0.7886	1.0701	1.1511	0.4545	2.9157
1	1.Yes	3.3617	0.5917	5.68	<.0001	0.05	2.2020	4.5213	28.8380	9.0434	91.9593
1	2.No	2.1251	0.5413	3.93	<.0001	0.05	1.0641	3.1860	8.3734	2.8982	24.1922

Differences of Highrisk1 Least Squares Means											
Highrisk1	*_Highrisk1*	*Estimate*	*Standard Error*	*z Value*	*Pr > |z|*	*Alpha*	*Lower*	*Upper*	*Exponentiated*	*Exponentiated Lower*	*Exponentiated Upper*
1.Yes	2.No	1.2366	0.5590	2.21	0.0269	0.05	0.1411	2.3322	3.4440	1.1515	10.3003

Model 19 Final Multivariable Ordinal logistic regression of ANC3 versus Group, site and confounders

The GENMOD Procedure

Model Information	
Data Set	WORK.CARDIAC2
Distribution	Multinomial
Link Function	Cumulative Logit
Dependent Variable	ANC3

Number of Observations Read	271
Number of Observations Used	232
Missing Values	39

Class Level Information

Class	*Levels*	*Values*
Revised_Groups	2	Acquired Pre-existant
Site	3	1 2 3
Highrisk1	2	1.Yes 2.No
Highrisk3	2	1.Yes 2.No

Response Profile

Ordered Value	*ANC3*	*Total Frequency*
1	3	23
2	2	86
3	1	87
4	0	36

PROC GENMOD is modeling the probabilities of levels of ANC3 having LOWER Ordered Values in the response profile table.

Parameter Information

Parameter	*Effect*	*Revised_Groups*	*Site*	*Highrisk1*	*Highrisk3*
Prm1	Revised_Groups	Acquired			
Prm2	Revised_Groups	Pre-existant			
Prm3	Site		1		
Prm4	Site		2		
Prm5	Site		3		
Prm6	Highrisk1			1.Yes	

Parameter Information					
Parameter	*Effect*	*Revised_Groups*	*Site*	*Highrisk1*	*Highrisk3*
Prm7	Highrisk1			2.No	
Prm8	Highrisk3				1.Yes
Prm9	Highrisk3				2.No

Criteria For Assessing Goodness Of Fit			
Criterion	*DF*	*Value*	*Value/DF*
Log Likelihood		-283.5509	
Full Log Likelihood		-283.5509	
AIC (smaller is better)		583.1019	
AICC (smaller is better)		583.7476	
BIC (smaller is better)		610.6758	

Algorithm converged.

Analysis Of Maximum Likelihood Parameter Estimates								
Parameter		*DF*	*Estimate*	*Standard Error*	*Wald 95% Confidence Limits*		*Wald Chi-Square*	*Pr > ChiSq*
Intercept1		1	-3.4221	0.4828	-4.3683	-2.4760	50.25	<.0001
Intercept2		1	-1.2686	0.4361	-2.1234	-0.4138	8.46	0.0036
Intercept3		1	0.6327	0.4317	-0.2134	1.4789	2.15	0.1428
Revised_Groups	Acquired	1	0.1680	0.2652	-0.3518	0.6878	0.40	0.5265
Revised_Groups	Pre-existant	0	0.0000	0.0000	0.0000	0.0000	.	.
Site	1	1	0.0790	0.4687	-0.8396	0.9976	0.03	0.8661
Site	2	1	-0.2259	0.2646	-0.7445	0.2927	0.73	0.3932
Site	3	0	0.0000	0.0000	0.0000	0.0000	.	.
Highrisk1	1.Yes	1	0.5423	0.2625	0.0277	1.0569	4.27	0.0389
Highrisk1	2.No	0	0.0000	0.0000	0.0000	0.0000	.	.

Analysis Of Maximum Likelihood Parameter Estimates								
					Wald 95% Confidence Limits			
Parameter		DF	Estimate	Standard Error			Wald Chi-Square	Pr > ChiSq
Highrisk3	1.Yes	1	0.9564	0.4113	0.1503	1.7625	5.41	0.0200
Highrisk3	2.No	0	0.0000	0.0000	0.0000	0.0000	.	.
Scale		0	1.0000	0.0000	1.0000	1.0000		

Note: The scale parameter was held fixed.

Wald Statistics For Type 3 Analysis			
Source	DF	Chi-Square	Pr > ChiSq
Revised_Groups	1	0.40	0.5265
Site	2	0.90	0.6365
Highrisk1	1	4.27	0.0389
Highrisk3	1	5.41	0.0200

Revised_Groups Least Squares Means

ANC3	Revised_Groups	Estimate	Standard Error	z Value	Pr > |z|	Alpha	Lower	Upper	Exponentiated	Exponentiated Lower	Exponentiated Upper
3	Acquired	-2.5538	0.3243	-7.87	<.0001	0.05	-3.1894	-1.9182	0.07779	0.04120	0.1469
3	Pre-existant	-2.7218	0.3313	-8.22	<.0001	0.05	-3.3711	-2.0724	0.06576	0.03435	0.1259
2	Acquired	-0.4002	0.2694	-1.49	0.1375	0.05	-0.9283	0.1279	0.6702	0.3952	1.1364
2	Pre-existant	-0.5682	0.2727	-2.08	0.0372	0.05	-1.1027	-0.03362	0.5666	0.3320	0.9669
1	Acquired	1.5011	0.2877	5.22	<.0001	0.05	0.9373	2.0649	4.4866	2.5531	7.8847
1	Pre-existant	1.3331	0.2887	4.62	<.0001	0.05	0.7674	1.8989	3.7929	2.1541	6.6784

Differences of Revised_Groups Least Squares Means

Revised_Groups	_Revised_Groups	Estimate	Standard Error	z Value	Pr > |z|	Alpha	Lower	Upper	Exponentiated	Exponentiated Lower	Exponentiated Upper
Acquired	Pre-existant	0.1680	0.2652	0.63	0.5265	0.05	-0.3518	0.6878	1.1829	0.7034	1.9893

Site Least Squares Means

ANC3	Site	Estimate	Standard Error	z Value	Pr > |z|	Alpha	Lower	Upper	Exponentiated	Exponentiated Lower	Exponentiated Upper
3	1	-2.5098	0.4933	-5.09	<.0001	0.05	-3.4767	-1.5429	0.08128	0.03091	0.2138
3	2	-2.8147	0.3213	-8.76	<.0001	0.05	-3.4444	-2.1850	0.05992	0.03192	0.1125
3	3	-2.5888	0.3059	-8.46	<.0001	0.05	-3.1883	-1.9893	0.07511	0.04124	0.1368
2	1	-0.3562	0.4611	-0.77	0.4398	0.05	-1.2599	0.5474	0.7003	0.2837	1.7288
2	2	-0.6611	0.2554	-2.59	0.0096	0.05	-1.1618	-0.1605	0.5163	0.3129	0.8517
2	3	-0.4352	0.2458	-1.77	0.0766	0.05	-0.9169	0.04648	0.6471	0.3997	1.0476
1	1	1.5451	0.4731	3.27	0.0011	0.05	0.6179	2.4723	4.6884	1.8550	11.8499
1	2	1.2402	0.2678	4.63	<.0001	0.05	0.7154	1.7650	3.4562	2.0450	5.8415
1	3	1.4661	0.2671	5.49	<.0001	0.05	0.9425	1.9896	4.3322	2.5665	7.3128

Differences of Site Least Squares Means

Site	_Site	Estimate	Standard Error	z Value	Pr > |z|	Alpha	Lower	Upper	Exponentiated	Exponentiated Lower	Exponentiated Upper
1	2	0.3049	0.4710	0.65	0.5174	0.05	-0.6182	1.2280	1.3565	0.5389	3.4145
1	3	0.07901	0.4687	0.17	0.8661	0.05	-0.8396	0.9976	1.0822	0.4319	2.7117
2	3	-0.2259	0.2646	-0.85	0.3932	0.05	-0.7445	0.2927	0.7978	0.4750	1.3400

Highrisk1 Least Squares Means

ANC3	Highrisk1	Estimate	Standard Error	z Value	Pr > |z|	Alpha	Lower	Upper	Exponentiated	Exponentiated Lower	Exponentiated Upper
3	1.Yes	-2.3666	0.3171	-7.46	<.0001	0.05	-2.9882	-1.7450	0.09380	0.05038	0.1746
3	2.No	-2.9089	0.3371	-8.63	<.0001	0.05	-3.5697	-2.2482	0.05453	0.02816	0.1056
2	1.Yes	-0.2130	0.2661	-0.80	0.4233	0.05	-0.7345	0.3084	0.8081	0.4797	1.3613
2	2.No	-0.7553	0.2747	-2.75	0.0060	0.05	-1.2938	-0.2168	0.4699	0.2742	0.8051
1	1.Yes	1.6883	0.2921	5.78	<.0001	0.05	1.1157	2.2608	5.4101	3.0518	9.5909
1	2.No	1.1460	0.2829	4.05	<.0001	0.05	0.5915	1.7004	3.1455	1.8067	5.4764

Differences of Highrisk1 Least Squares Means											
Highrisk1	_Highrisk1	Estimate	Standard Error	z Value	Pr > |z|	Alpha	Lower	Upper	Exponentiated	Exponentiated Lower	Exponentiated Upper
1.Yes	2.No	0.5423	0.2625	2.07	0.0389	0.05	0.02774	1.0569	1.7200	1.0281	2.8773

Highrisk3 Least Squares Means											
ANC3	Highrisk3	Estimate	Standard Error	z Value	Pr > |z|	Alpha	Lower	Upper	Exponentiated	Exponentiated Lower	Exponentiated Upper
3	1.Yes	-2.1596	0.2558	-8.44	<.0001	0.05	-2.6610	-1.6581	0.1154	0.06988	0.1905
3	2.No	-3.1160	0.4460	-6.99	<.0001	0.05	-3.9901	-2.2419	0.04434	0.01850	0.1063
2	1.Yes	-0.00598	0.1886	-0.03	0.9747	0.05	-0.3757	0.3637	0.9940	0.6868	1.4387
2	2.No	-0.9624	0.4010	-2.40	0.0164	0.05	-1.7483	-0.1764	0.3820	0.1741	0.8382
1	1.Yes	1.8953	0.2330	8.13	<.0001	0.05	1.4387	2.3520	6.6547	4.2150	10.5065
1	2.No	0.9389	0.4015	2.34	0.0194	0.05	0.1520	1.7258	2.5572	1.1642	5.6171

Differences of Highrisk3 Least Squares Means											
Highrisk3	_Highrisk3	Estimate	Standard Error	z Value	Pr > |z|	Alpha	Lower	Upper	Exponentiated	Exponentiated Lower	Exponentiated Upper
1.Yes	2.No	0.9564	0.4113	2.33	0.0200	0.05	0.1503	1.7625	2.6023	1.1622	5.8269

Model 2 Final Multivariable Logistic regression of ANCQ versus Group, site and confounders

The GENMOD Procedure

Model Information	
Data Set	WORK.CARDIAC2
Distribution	Binomial
Link Function	Logit
Dependent Variable	ANCQ

Number of Observations Read	271
Number of Observations Used	253
Number of Events	167
Number of Trials	253
Missing Values	18

Class Level Information		
Class	*Levels*	*Values*
Revised_Groups	2	Acquired Pre-existant
Site	3	1 2 3
EPPSD_scoreRECORDED	2	1.Yes 2.No

Response Profile		
Ordered Value	*ANCQ*	*Total Frequency*
1	1	167
2	0	86

PROC GENMOD is modeling the probability that ANCQ='1'.

Parameter Information				
Parameter	*Effect*	*Revised_Groups*	*Site*	*EPPSD_scoreRECORDED*
Prm1	Intercept			
Prm2	Revised_Groups	Acquired		
Prm3	Revised_Groups	Pre-existant		
Prm4	Site		1	
Prm5	Site		2	
Prm6	Site		3	
Prm7	EPPSD_scoreRECORDED			1.Yes
Prm8	EPPSD_scoreRECORDED			2.No
Prm9	Live_baby_weight			

Criteria For Assessing Goodness Of Fit			
Criterion	*DF*	*Value*	*Value/DF*
Log Likelihood		-91.7271	
Full Log Likelihood		-91.7271	
AIC (smaller is better)		195.4542	
AICC (smaller is better)		195.7957	
BIC (smaller is better)		216.6546	

Algorithm converged.

Analysis Of Maximum Likelihood Parameter Estimates									
					Wald 95% Confidence Limits				
Parameter		*DF*	*Estimate*	*Standard Error*			*Wald Chi-Square*	*Pr > ChiSq*
Intercept		1	1.4071	0.8562	-0.2709	3.0852	2.70	0.1003
Revised_Groups	Acquired	1	-0.9397	0.3823	-1.6890	-0.1904	6.04	0.0140
Revised_Groups	Pre-existant	0	0.0000	0.0000	0.0000	0.0000	.	.
Site	1	1	0.9360	0.8065	-0.6448	2.5168	1.35	0.2458
Site	2	1	1.3011	0.3881	0.5404	2.0618	11.24	0.0008
Site	3	0	0.0000	0.0000	0.0000	0.0000	.	.
EPPSD_scoreRECORDED	1.Yes	1	4.3183	0.7602	2.8283	5.8084	32.26	<.0001
EPPSD_scoreRECORDED	2.No	0	0.0000	0.0000	0.0000	0.0000	.	.
Live_baby_weight		1	-0.0006	0.0003	-0.0011	-0.0001	5.64	0.0176
Scale		0	1.0000	0.0000	1.0000	1.0000		

Note: The scale parameter was held fixed.

Wald Statistics For Type 3 Analysis

Source	DF	Chi-Square	Pr > ChiSq
Revised_Groups	1	6.04	0.0140
Site	2	11.57	0.0031
EPPSD_scoreRECORDED	1	32.26	<.0001
Live_baby_weight	1	5.64	0.0176

Contrast Estimate Results

Label	Mean Estimate	Mean Confidence Limits		L'Beta Estimate	Standard Error	Alpha	L'Beta Confidence Limits		Chi-Square	Pr > ChiSq
Live_baby_weight	0.4998	0.4997	0.5000	-0.0006	0.0003	0.05	-0.0011	-0.0001	5.64	0.0176
Exp(Live_baby_weight)				0.9994	0.0003	0.05	0.9989	0.9999		

Revised_Groups Least Squares Means

Revised_Groups	Estimate	Standard Error	z Value	Pr > |z|	Alpha	Lower	Upper	Exponentiated	Exponentiated Lower	Exponentiated Upper
Acquired	1.4831	0.4304	3.45	0.0006	0.05	0.6396	2.3266	4.4066	1.8958	10.2429
Pre-existant	2.4228	0.4740	5.11	<.0001	0.05	1.4937	3.3519	11.2776	4.4538	28.5568

Differences of Revised_Groups Least Squares Means

Revised_Groups	_Revised_Groups	Estimate	Standard Error	z Value	Pr > |z|	Alpha	Lower	Upper	Exponentiated	Exponentiated Lower	Exponentiated Upper
Acquired	Pre-existant	-0.9397	0.3823	-2.46	0.0140	0.05	-1.6890	-0.1904	0.3907	0.1847	0.8266

Site Least Squares Means

Site	Estimate	Standard Error	z Value	Pr > |z|	Alpha	Lower	Upper	Exponentiated	Exponentiated Lower	Exponentiated Upper
1	2.1433	0.7914	2.71	0.0068	0.05	0.5921	3.6944	8.5273	1.8078	40.2219
2	2.5084	0.4491	5.58	<.0001	0.05	1.6281	3.3887	12.2849	5.0940	29.6271
3	1.2072	0.4178	2.89	0.0039	0.05	0.3884	2.0260	3.3443	1.4747	7.5840

Differences of Site Least Squares Means											
Site	*_Site*	*Estimate*	*Standard Error*	*z Value*	*Pr > |z|*	*Alpha*	*Lower*	*Upper*	*Exponentiated*	*Exponentiated Lower*	*Exponentiated Upper*
1	2	-0.3651	0.8312	-0.44	0.6605	0.05	-1.9942	1.2640	0.6941	0.1361	3.5397
1	3	0.9360	0.8065	1.16	0.2458	0.05	-0.6448	2.5168	2.5498	0.5248	12.3892
2	3	1.3011	0.3881	3.35	0.0008	0.05	0.5404	2.0618	3.6734	1.7167	7.8604

EPPSD_scoreRECORDED Least Squares Means										
EPPSD_scoreRECORDED	*Estimate*	*Standard Error*	*z Value*	*Pr > |z|*	*Alpha*	*Lower*	*Upper*	*Exponentiated*	*Exponentiated Lower*	*Exponentiated Upper*
1.Yes	4.1121	0.7351	5.59	<.0001	0.05	2.6714	5.5528	61.0765	14.4605	257.97
2.No	-0.2062	0.2924	-0.71	0.4807	0.05	-0.7793	0.3669	0.8137	0.4587	1.4432

Differences of EPPSD_scoreRECORDED Least Squares Means											
EPPSD_scoreRECORDED	*_EPPSD_scoreRECORDED*	*Estimate*	*Standard Error*	*z Value*	*Pr > |z|*	*Alpha*	*Lower*	*Upper*	*Exponentiated*	*Exponentiated Lower*	*Exponentiated Upper*
1.Yes	2.No	4.3183	0.7602	5.68	<.0001	0.05	2.8283	5.8084	75.0630	16.9163	333.08

Model 20 Final Multivariable Ordinal logistic regression of FetalRisk1_sum versus Group, site and confounders

The GENMOD Procedure

Model Information	
Data Set	WORK.CARDIAC2
Distribution	Multinomial
Link Function	Cumulative Logit
Dependent Variable	FetalRisk1_sum

Number of Observations Read	271

Number of Observations Used	133
Missing Values	138

Class Level Information		
Class	*Levels*	*Values*
Revised_Groups	2	Acquired Pre-existant
Site	3	1 2 3
Highrisk1	2	1.Yes 2.No
Highrisk7	2	1.Yes 2.No

Response Profile		
Ordered Value	*FetalRisk1_sum*	*Total Frequency*
1	3	98
2	2	26
3	1	9

PROC GENMOD is modeling the probabilities of levels of FetalRisk1_sum having LOWER Ordered Values in the response profile table.

Parameter Information					
Parameter	*Effect*	*Revised_Groups*	*Site*	*Highrisk1*	*Highrisk7*
Prm1	Revised_Groups	Acquired			
Prm2	Revised_Groups	Pre-existant			
Prm3	Site		1		
Prm4	Site		2		
Prm5	Site		3		
Prm6	Highrisk1			1.Yes	
Prm7	Highrisk1			2.No	
Prm8	Highrisk7				1.Yes
Prm9	Highrisk7				2.No

Criteria For Assessing Goodness Of Fit			
Criterion	*DF*	*Value*	*Value/DF*
Log Likelihood		-76.7630	
Full Log Likelihood		-76.7630	
AIC (smaller is better)		167.5260	
AICC (smaller is better)		168.4220	
BIC (smaller is better)		187.7584	

Algorithm converged.

Analysis Of Maximum Likelihood Parameter Estimates									
Parameter		*DF*	*Estimate*	*Standard Error*	*Wald 95% Confidence Limits*		*Wald Chi-Square*	*Pr > ChiSq*
Intercept1		1	-1.4178	0.4913	-2.3808	-0.4549	8.33	0.0039
Intercept2		1	0.6557	0.4916	-0.3077	1.6191	1.78	0.1822
Revised_Groups	Acquired	1	-0.0279	0.4523	-0.9144	0.8587	0.00	0.9509
Revised_Groups	Pre-existant	0	0.0000	0.0000	0.0000	0.0000	.	.
Site	1	1	0.8848	0.8506	-0.7824	2.5520	1.08	0.2983
Site	2	1	1.4718	0.5413	0.4108	2.5329	7.39	0.0066
Site	3	0	0.0000	0.0000	0.0000	0.0000	.	.
Highrisk1	1.Yes	1	1.4584	0.5089	0.4610	2.4558	8.21	0.0042
Highrisk1	2.No	0	0.0000	0.0000	0.0000	0.0000	.	.
Highrisk7	1.Yes	1	1.3284	0.5231	0.3031	2.3537	6.45	0.0111
Highrisk7	2.No	0	0.0000	0.0000	0.0000	0.0000	.	.
Scale		0	1.0000	0.0000	1.0000	1.0000		

Note: The scale parameter was held fixed.

Wald Statistics For Type 3 Analysis			
Source	DF	Chi-Square	Pr > ChiSq
Revised_Groups	1	0.00	0.9509
Site	2	7.78	0.0204
Highrisk1	1	8.21	0.0042
Highrisk7	1	6.45	0.0111

Revised_Groups Least Squares Means

FetalRisk1_sum	Revised_Groups	Estimate	Standard Error	z Value	Pr > |z|	Alpha	Lower	Upper	Exponentiated	Exponentiated Lower	Exponentiated Upper
3	Acquired	0.7332	0.4267	1.72	0.0857	0.05	-0.1030	1.5695	2.0818	0.9021	4.8041
3	Pre-existant	0.7611	0.3856	1.97	0.0484	0.05	0.005414	1.5168	2.1406	1.0054	4.5575
2	Acquired	2.8068	0.5307	5.29	<.0001	0.05	1.7666	3.8469	16.5564	5.8510	46.8493
2	Pre-existant	2.8346	0.4980	5.69	<.0001	0.05	1.8587	3.8106	17.0241	6.4151	45.1772

Differences of Revised_Groups Least Squares Means

Revised_Groups	_Revised_Groups	Estimate	Standard Error	z Value	Pr > |z|	Alpha	Lower	Upper	Exponentiated	Exponentiated Lower	Exponentiated Upper
Acquired	Pre-existant	-0.02786	0.4523	-0.06	0.9509	0.05	-0.9144	0.8587	0.9725	0.4008	2.3600

Site Least Squares Means

FetalRisk1_sum	Site	Estimate	Standard Error	z Value	Pr > |z|	Alpha	Lower	Upper	Exponentiated	Exponentiated Lower	Exponentiated Upper
3	1	0.8464	0.8200	1.03	0.3020	0.05	-0.7607	2.4536	2.3313	0.4673	11.6296
3	2	1.4335	0.4635	3.09	0.0020	0.05	0.5250	2.3419	4.1932	1.6905	10.4012
3	3	-0.03838	0.2918	-0.13	0.8953	0.05	-0.6102	0.5334	0.9623	0.5432	1.7048
2	1	2.9199	0.8796	3.32	0.0009	0.05	1.1960	4.6438	18.5403	3.3070	103.94
2	2	3.5070	0.5890	5.95	<.0001	0.05	2.3526	4.6614	33.3481	10.5127	105.79
2	3	2.0352	0.3875	5.25	<.0001	0.05	1.2757	2.7946	7.6534	3.5811	16.3567

Differences of Site Least Squares Means

Site	_Site	Estimate	Standard Error	z Value	Pr > |z|	Alpha	Lower	Upper	Exponentiated	Exponentiated Lower	Exponentiated Upper
1	2	-0.5871	0.9341	-0.63	0.5297	0.05	-2.4179	1.2438	0.5560	0.08911	3.4687
1	3	0.8848	0.8506	1.04	0.2983	0.05	-0.7824	2.5520	2.4225	0.4573	12.8331
2	3	1.4718	0.5413	2.72	0.0066	0.05	0.4108	2.5329	4.3573	1.5081	12.5894

Highrisk1 Least Squares Means

FetalRisk1_sum	Highrisk1	Estimate	Standard Error	z Value	Pr > |z|	Alpha	Lower	Upper	Exponentiated	Exponentiated Lower	Exponentiated Upper
3	1.Yes	1.4764	0.4265	3.46	0.0005	0.05	0.6404	2.3123	4.3770	1.8973	10.0977
3	2.No	0.01797	0.4195	0.04	0.9658	0.05	-0.8042	0.8401	1.0181	0.4475	2.3167
2	1.Yes	3.5499	0.5632	6.30	<.0001	0.05	2.4460	4.6538	34.8095	11.5417	104.99
2	2.No	2.0915	0.4894	4.27	<.0001	0.05	1.1322	3.0508	8.0971	3.1026	21.1319

Differences of Highrisk1 Least Squares Means

Highrisk1	_Highrisk1	Estimate	Standard Error	z Value	Pr > |z|	Alpha	Lower	Upper	Exponentiated	Exponentiated Lower	Exponentiated Upper
1.Yes	2.No	1.4584	0.5089	2.87	0.0042	0.05	0.4610	2.4558	4.2990	1.5857	11.6554

Highrisk7 Least Squares Means

FetalRisk1_sum	Highrisk7	Estimate	Standard Error	z Value	Pr > |z|	Alpha	Lower	Upper	Exponentiated	Exponentiated Lower	Exponentiated Upper
3	1.Yes	1.4114	0.3608	3.91	<.0001	0.05	0.7042	2.1185	4.1015	2.0222	8.3188
3	2.No	0.08297	0.4848	0.17	0.8641	0.05	-0.8673	1.0332	1.0865	0.4201	2.8100
2	1.Yes	3.4849	0.5083	6.86	<.0001	0.05	2.4886	4.4812	32.6190	12.0442	88.3413
2	2.No	2.1565	0.5529	3.90	<.0001	0.05	1.0728	3.2402	8.6409	2.9235	25.5398

Differences of Highrisk7 Least Squares Means											
Highrisk7	_Highrisk7	Estimate	Standard Error	z Value	Pr > |z|	Alpha	Lower	Upper	Exponentiated	Exponentiated Lower	Exponentiated Upper
1.Yes	2.No	1.3284	0.5231	2.54	0.0111	0.05	0.3031	2.3537	3.7750	1.3540	10.5245

Model 21 Final Multivariable Logistic regression of FetalRisk2_sum versus Group, site and confounders

The GENMOD Procedure

Model Information	
Data Set	WORK.CARDIAC2
Distribution	Binomial
Link Function	Logit
Dependent Variable	FetalRisk2_sum

Number of Observations Read	271
Number of Observations Used	85
Number of Events	21
Number of Trials	85
Missing Values	186

Class Level Information		
Class	Levels	Values
Revised_Groups	2	Acquired Pre-existant
Site	3	1 2 3

Response Profile		
Ordered Value	FetalRisk2_sum	Total Frequency
1	2	21

Response Profile		
Ordered Value	FetalRisk2_sum	Total Frequency
2	1	64

PROC GENMOD is modeling the probability that FetalRisk2_sum='2'.

Parameter Information			
Parameter	Effect	Revised_Groups	Site
Prm1	Intercept		
Prm2	Revised_Groups	Acquired	
Prm3	Revised_Groups	Pre-existant	
Prm4	Site		1
Prm5	Site		2
Prm6	Site		3

Criteria For Assessing Goodness Of Fit			
Criterion	DF	Value	Value/DF
Log Likelihood		-47.2074	
Full Log Likelihood		-47.2074	
AIC (smaller is better)		102.4147	
AICC (smaller is better)		102.9147	
BIC (smaller is better)		112.1853	

Algorithm converged.

Analysis Of Maximum Likelihood Parameter Estimates							
Parameter	DF	Estimate	Standard Error	Wald 95% Confidence Limits		Wald Chi-Square	Pr > ChiSq
Intercept	1	-1.0323	0.4130	-1.8417	-0.2228	6.25	0.0124

Analysis Of Maximum Likelihood Parameter Estimates								
Parameter		*DF*	*Estimate*	*Standard Error*	*Wald 95% Confidence Limits*		*Wald Chi-Square*	*Pr > ChiSq*
Revised_Groups	Acquired	1	-0.3416	0.5440	-1.4078	0.7247	0.39	0.5301
Revised_Groups	Pre-existant	0	0.0000	0.0000	0.0000	0.0000	.	.
Site	1	1	-0.0551	0.7581	-1.5409	1.4308	0.01	0.9421
Site	2	1	0.1561	0.5562	-0.9340	1.2462	0.08	0.7790
Site	3	0	0.0000	0.0000	0.0000	0.0000	.	.
Scale		0	1.0000	0.0000	1.0000	1.0000		

Note: The scale parameter was held fixed.

Wald Statistics For Type 3 Analysis			
Source	*DF*	*Chi-Square*	*Pr > ChiSq*
Revised_Groups	1	0.39	0.5301
Site	2	0.11	0.9488

Revised_Groups Least Squares Means										
Revised_Groups	*Estimate*	*Standard Error*	*z Value*	*Pr > |z|*	*Alpha*	*Lower*	*Upper*	*Exponentiated*	*Exponentiated Lower*	*Exponentiated Upper*
Acquired	-1.3402	0.4328	-3.10	0.0020	0.05	-2.1885	-0.4918	0.2618	0.1121	0.6115
Pre-existant	-0.9986	0.3591	-2.78	0.0054	0.05	-1.7024	-0.2948	0.3684	0.1822	0.7447

Differences of Revised_Groups Least Squares Means											
Revised_Groups	*_Revised_Groups*	*Estimate*	*Standard Error*	*z Value*	*Pr > |z|*	*Alpha*	*Lower*	*Upper*	*Exponentiated*	*Exponentiated Lower*	*Exponentiated Upper*
Acquired	Pre-existant	-0.3416	0.5440	-0.63	0.5301	0.05	-1.4078	0.7247	0.7106	0.2447	2.0641

Site Least Squares Means										
Site	Estimate	Standard Error	z Value	Pr > |z|	Alpha	Lower	Upper	Exponentiated	Exponentiated Lower	Exponentiated Upper
1	-1.2581	0.6551	-1.92	0.0548	0.05	-2.5420	0.02577	0.2842	0.07871	1.0261
2	-1.0470	0.4390	-2.38	0.0171	0.05	-1.9074	-0.1866	0.3510	0.1485	0.8298
3	-1.2031	0.3709	-3.24	0.0012	0.05	-1.9300	-0.4761	0.3003	0.1451	0.6212

Differences of Site Least Squares Means											
Site	_Site	Estimate	Standard Error	z Value	Pr > |z|	Alpha	Lower	Upper	Exponentiated	Exponentiated Lower	Exponentiated Upper
1	2	-0.2112	0.7979	-0.26	0.7913	0.05	-1.7750	1.3527	0.8096	0.1695	3.8679
1	3	-0.05506	0.7581	-0.07	0.9421	0.05	-1.5409	1.4308	0.9464	0.2142	4.1821
2	3	0.1561	0.5562	0.28	0.7790	0.05	-0.9340	1.2462	1.1689	0.3930	3.4769

Model 3 Final Multivariable Logistic regression of Plannedcare1 versus Group, site and confounders

The GENMOD Procedure

Model Information	
Data Set	WORK.CARDIAC2
Distribution	Binomial
Link Function	Logit
Dependent Variable	Plannedcare1

Number of Observations Read	271
Number of Observations Used	197
Number of Events	159
Number of Trials	197
Missing Values	74

Class Level Information		
Class	*Levels*	*Values*
Revised_Groups	2	Acquired Pre-existant
Site	3	1 2 3
Highrisk1	2	1.Yes 2.No
Highrisk3	2	1.Yes 2.No

Response Profile		
Ordered Value	*Plannedcare1*	*Total Frequency*
1	Yes	159
2	No	38

PROC GENMOD is modeling the probability that Plannedcare1='Yes'.

Parameter Information					
Parameter	*Effect*	*Revised_Groups*	*Site*	*Highrisk1*	*Highrisk3*
Prm1	Intercept				
Prm2	Revised_Groups	Acquired			
Prm3	Revised_Groups	Pre-existant			
Prm4	Site		1		
Prm5	Site		2		
Prm6	Site		3		
Prm7	Highrisk1			1.Yes	
Prm8	Highrisk1			2.No	
Prm9	Highrisk3				1.Yes
Prm10	Highrisk3				2.No

Criteria For Assessing Goodness Of Fit			
Criterion	*DF*	*Value*	*Value/DF*
Log Likelihood		-78.6963	

Criteria For Assessing Goodness Of Fit			
Criterion	*DF*	*Value*	*Value/DF*
Full Log Likelihood		-78.6963	
AIC (smaller is better)		169.3927	
AICC (smaller is better)		169.8348	
BIC (smaller is better)		189.0919	

Algorithm converged.

Analysis Of Maximum Likelihood Parameter Estimates								
Parameter		*DF*	*Estimate*	*Standard Error*	*Wald 95% Confidence Limits*		*Wald Chi-Square*	*Pr > ChiSq*
Intercept		1	-1.0108	0.5867	-2.1607	0.1391	2.97	0.0849
Revised_Groups	Acquired	1	-0.6683	0.4353	-1.5214	0.1849	2.36	0.1247
Revised_Groups	Pre-existant	0	0.0000	0.0000	0.0000	0.0000	.	.
Site	1	1	24.6415	84674.54	-165934	165983.7	0.00	0.9998
Site	2	1	0.4068	0.4206	-0.4176	1.2312	0.94	0.3335
Site	3	0	0.0000	0.0000	0.0000	0.0000	.	.
Highrisk1	1.Yes	1	0.9403	0.4312	0.0951	1.7854	4.75	0.0292
Highrisk1	2.No	0	0.0000	0.0000	0.0000	0.0000	.	.
Highrisk3	1.Yes	1	2.2374	0.5631	1.1337	3.3411	15.79	<.0001
Highrisk3	2.No	0	0.0000	0.0000	0.0000	0.0000	.	.
Scale		0	1.0000	0.0000	1.0000	1.0000		

Note: The scale parameter was held fixed.

Wald Statistics For Type 3 Analysis			
Source	*DF*	*Chi-Square*	*Pr > ChiSq*
Revised_Groups	1	2.36	0.1247
Site	2	0.94	0.6265

Wald Statistics For Type 3 Analysis			
Source	DF	Chi-Square	Pr > ChiSq
Highrisk1	1	4.75	0.0292
Highrisk3	1	15.79	<.0001

Revised_Groups Least Squares Means

Revised_Groups	Estimate	Standard Error	z Value	Pr > |z|	Alpha	Lower	Upper	Exponentiated	Exponentiated Lower	Exponentiated Upper
Acquired	8.2592	28225	0.00	0.9998	0.05	-55311	55328	3862.96	0	Infty
Pre-existant	8.9274	28225	0.00	0.9997	0.05	-55311	55329	7535.97	0	Infty

Differences of Revised_Groups Least Squares Means

Revised_Groups	_Revised_Groups	Estimate	Standard Error	z Value	Pr > |z|	Alpha	Lower	Upper	Exponentiated	Exponentiated Lower	Exponentiated Upper
Acquired	Pre-existant	-0.6683	0.4353	-1.54	0.1247	0.05	-1.5214	0.1849	0.5126	0.2184	1.2031

Site Least Squares Means

Site	Estimate	Standard Error	z Value	Pr > |z|	Alpha	Lower	Upper	Exponentiated	Exponentiated Lower	Exponentiated Upper
1	24.8854	84675	0.00	0.9998	0.05	-165934	165984	6.421E10	0	Infty
2	0.6507	0.3572	1.82	0.0685	0.05	-0.04944	1.3508	1.9169	0.9518	3.8606
3	0.2439	0.3471	0.70	0.4823	0.05	-0.4364	0.9242	1.2762	0.6463	2.5199

Differences of Site Least Squares Means

Site	_Site	Estimate	Standard Error	z Value	Pr > |z|	Alpha	Lower	Upper	Exponentiated	Exponentiated Lower	Exponentiated Upper
1	2	24.2347	84675	0.00	0.9998	0.05	-165935	165983	3.35E10	0	Infty
1	3	24.6415	84675	0.00	0.9998	0.05	-165934	165984	5.031E10	0	Infty
2	3	0.4068	0.4206	0.97	0.3335	0.05	-0.4176	1.2312	1.5020	0.6586	3.4254

Highrisk1 Least Squares Means

Highrisk1	*Estimate*	*Standard Error*	*z Value*	*Pr > |z|*	*Alpha*	*Lower*	*Upper*	*Exponentiated*	*Exponentiated Lower*	*Exponentiated Upper*
1.Yes	9.0635	28225	0.00	0.9997	0.05	-55311	55329	8633.96	0	Infty
2.No	8.1232	28225	0.00	0.9998	0.05	-55312	55328	3371.70	0	Infty

Differences of Highrisk1 Least Squares Means

Highrisk1	*_Highrisk1*	*Estimate*	*Standard Error*	*z Value*	*Pr > |z|*	*Alpha*	*Lower*	*Upper*	*Exponentiated*	*Exponentiated Lower*	*Exponentiated Upper*
1.Yes	2.No	0.9403	0.4312	2.18	0.0292	0.05	0.09513	1.7854	2.5607	1.0998	5.9622

Highrisk3 Least Squares Means

Highrisk3	*Estimate*	*Standard Error*	*z Value*	*Pr > |z|*	*Alpha*	*Lower*	*Upper*	*Exponentiated*	*Exponentiated Lower*	*Exponentiated Upper*
1.Yes	9.7120	28225	0.00	0.9997	0.05	-55310	55329	16515	0	Infty
2.No	7.4746	28225	0.00	0.9998	0.05	-55312	55327	1762.74	0	Infty

Differences of Highrisk3 Least Squares Means

Highrisk3	*_Highrisk3*	*Estimate*	*Standard Error*	*z Value*	*Pr > |z|*	*Alpha*	*Lower*	*Upper*	*Exponentiated*	*Exponentiated Lower*	*Exponentiated Upper*
1.Yes	2.No	2.2374	0.5631	3.97	<.0001	0.05	1.1337	3.3411	9.3688	3.1070	28.2506

Model 4 Final Multivariable Logistic regression of Plannedcare2 versus Group, site and confounders

The GENMOD Procedure

Model Information

Data Set	WORK.CARDIAC2
Distribution	Binomial
Link Function	Logit
Dependent Variable	Plannedcare2

Number of Observations Read	271
Number of Observations Used	81
Number of Events	14
Number of Trials	81
Missing Values	190

Class Level Information

Class	*Levels*	*Values*
Revised_Groups	2	Acquired Pre-existant
Site	3	1 2 3
Highrisk1	2	1.Yes 2.No

Response Profile

Ordered Value	*Plannedcare2*	*Total Frequency*
1	Yes	14
2	No	67

PROC GENMOD is modeling the probability that Plannedcare2='Yes'.

Parameter Information

Parameter	*Effect*	*Revised_Groups*	*Site*	*Highrisk1*
Prm1	Intercept			
Prm2	Revised_Groups	Acquired		
Prm3	Revised_Groups	Pre-existant		
Prm4	Site		1	
Prm5	Site		2	
Prm6	Site		3	
Prm7	Highrisk1			1.Yes

Parameter Information				
Parameter	Effect	Revised_Groups	Site	Highrisk1
Prm8	Highrisk1			2.No
Prm9	Parity			

Criteria For Assessing Goodness Of Fit			
Criterion	DF	Value	Value/DF
Log Likelihood		-31.0770	
Full Log Likelihood		-31.0770	
AIC (smaller is better)		74.1541	
AICC (smaller is better)		75.2892	
BIC (smaller is better)		88.5208	

Algorithm converged.

Analysis Of Maximum Likelihood Parameter Estimates

Parameter		DF	Estimate	Standard Error	Wald 95% Confidence Limits		Wald Chi-Square	Pr > ChiSq
Intercept		1	-3.5006	0.9487	-5.3601	-1.6411	13.61	0.0002
Revised_Groups	Acquired	1	0.4062	0.6989	-0.9636	1.7759	0.34	0.5611
Revised_Groups	Pre-existant	0	0.0000	0.0000	0.0000	0.0000	.	.
Site	1	1	-0.0460	1.4826	-2.9519	2.8599	0.00	0.9753
Site	2	1	-0.2160	0.6851	-1.5587	1.1267	0.10	0.7525
Site	3	0	0.0000	0.0000	0.0000	0.0000	.	.
Highrisk1	1.Yes	1	1.6620	0.8449	0.0061	3.3179	3.87	0.0492
Highrisk1	2.No	0	0.0000	0.0000	0.0000	0.0000	.	.
Parity		1	0.4665	0.2296	0.0165	0.9164	4.13	0.0421
Scale		0	1.0000	0.0000	1.0000	1.0000		

Note: The scale parameter was held fixed.

Wald Statistics For Type 3 Analysis			
Source	*DF*	*Chi-Square*	*Pr > ChiSq*
Revised_Groups	1	0.34	0.5611
Site	2	0.10	0.9506
Highrisk1	1	3.87	0.0492
Parity	1	4.13	0.0421

Contrast Estimate Results

Label	*Mean Estimate*	*Mean Confidence Limits*		*L'Beta Estimate*	*Standard Error*	*Alpha*	*L'Beta Confidence Limits*		*Chi-Square*	*Pr > ChiSq*
Parity	0.6145	0.5041	0.7143	0.4665	0.2296	0.05	0.0165	0.9164	4.13	0.0421
Exp(Parity)				1.5944	0.3660	0.05	1.0167	2.5002		

Revised_Groups Least Squares Means

Revised_Groups	*Estimate*	*Standard Error*	*z Value*	*Pr > |z|*	*Alpha*	*Lower*	*Upper*	*Exponentiated*	*Exponentiated Lower*	*Exponentiated Upper*
Acquired	-1.7000	0.8393	-2.03	0.0428	0.05	-3.3450	-0.05504	0.1827	0.03526	0.9464
Pre-existant	-2.1062	0.6224	-3.38	0.0007	0.05	-3.3261	-0.8863	0.1217	0.03593	0.4122

Differences of Revised_Groups Least Squares Means

Revised_Groups	*_Revised_Groups*	*Estimate*	*Standard Error*	*z Value*	*Pr > |z|*	*Alpha*	*Lower*	*Upper*	*Exponentiated*	*Exponentiated Lower*	*Exponentiated Upper*
Acquired	Pre-existant	0.4062	0.6989	0.58	0.5611	0.05	-0.9636	1.7759	1.5011	0.3815	5.9058

Site Least Squares Means

Site	*Estimate*	*Standard Error*	*z Value*	*Pr > |z|*	*Alpha*	*Lower*	*Upper*	*Exponentiated*	*Exponentiated Lower*	*Exponentiated Upper*
1	-1.8618	1.5021	-1.24	0.2152	0.05	-4.8058	1.0823	0.1554	0.008182	2.9513

Site Least Squares Means										
Site	*Estimate*	*Standard Error*	*z Value*	*Pr > |z|*	*Alpha*	*Lower*	*Upper*	*Exponentiated*	*Exponentiated Lower*	*Exponentiated Upper*
2	-2.0318	0.5389	-3.77	0.0002	0.05	-3.0879	-0.9756	0.1311	0.04560	0.3770
3	-1.8158	0.5864	-3.10	0.0020	0.05	-2.9650	-0.6665	0.1627	0.05156	0.5135

Differences of Site Least Squares Means											
Site	*_Site*	*Estimate*	*Standard Error*	*z Value*	*Pr > |z|*	*Alpha*	*Lower*	*Upper*	*Exponentiated*	*Exponentiated Lower*	*Exponentiated Upper*
1	2	0.1700	1.4798	0.11	0.9085	0.05	-2.7304	3.0704	1.1853	0.06520	21.5501
1	3	-0.04599	1.4826	-0.03	0.9753	0.05	-2.9519	2.8599	0.9550	0.05224	17.4594
2	3	-0.2160	0.6851	-0.32	0.7525	0.05	-1.5587	1.1267	0.8057	0.2104	3.0854

Highrisk1 Least Squares Means										
Highrisk1	*Estimate*	*Standard Error*	*z Value*	*Pr > |z|*	*Alpha*	*Lower*	*Upper*	*Exponentiated*	*Exponentiated Lower*	*Exponentiated Upper*
1.Yes	-1.0721	0.5614	-1.91	0.0562	0.05	-2.1725	0.02830	0.3423	0.1139	1.0287
2.No	-2.7341	0.9430	-2.90	0.0037	0.05	-4.5823	-0.8859	0.06495	0.01023	0.4124

Differences of Highrisk1 Least Squares Means											
Highrisk1	*_Highrisk1*	*Estimate*	*Standard Error*	*z Value*	*Pr > |z|*	*Alpha*	*Lower*	*Upper*	*Exponentiated*	*Exponentiated Lower*	*Exponentiated Upper*
1.Yes	2.No	1.6620	0.8449	1.97	0.0492	0.05	0.006088	3.3179	5.2697	1.0061	27.6012

Model 4 Final Multivariable Logistic regression of Plannedcare2 versus Group, site and confounders

The GENMOD Procedure

Model Information	
Data Set	WORK.CARDIAC2
Distribution	Binomial

Model Information	
Link Function	Logit
Dependent Variable	Plannedcare3x

Number of Observations Read	271
Number of Observations Used	198
Number of Events	98
Number of Trials	198
Missing Values	73

Class Level Information

Class	*Levels*	*Values*
Revised_Groups	2	Acquired Pre-existant
Site	3	1 2 3
Highrisk5_sum	3	1 2 3

Response Profile

Ordered Value	*Plannedcare3x*	*Total Frequency*
1	Vaginal	98
2	Caesarean	100

PROC GENMOD is modeling the probability that Plannedcare3x='Vaginal'.

Parameter Information

Parameter	*Effect*	*Revised_Groups*	*Site*	*Highrisk5_sum*
Prm1	Intercept			
Prm2	Revised_Groups	Acquired		
Prm3	Revised_Groups	Pre-existant		
Prm4	Site		1	

Parameter Information				
Parameter	Effect	Revised_Groups	Site	Highrisk5_sum
Prm5	Site		2	
Prm6	Site		3	
Prm7	Highrisk5_sum			1
Prm8	Highrisk5_sum			2
Prm9	Highrisk5_sum			3
Prm10	team_sum			

Criteria For Assessing Goodness Of Fit			
Criterion	DF	Value	Value/DF
Log Likelihood		-112.2955	
Full Log Likelihood		-112.2955	
AIC (smaller is better)		238.5910	
AICC (smaller is better)		239.1805	
BIC (smaller is better)		261.6089	

Algorithm converged.

Analysis Of Maximum Likelihood Parameter Estimates								
Parameter		DF	Estimate	Standard Error	Wald 95% Confidence Limits		Wald Chi-Square	Pr > ChiSq
Intercept		1	1.8388	0.7264	0.4150	3.2626	6.41	0.0114
Revised_Groups	Acquired	1	-0.1068	0.3393	-0.7719	0.5583	0.10	0.7530
Revised_Groups	Pre-existant	0	0.0000	0.0000	0.0000	0.0000	.	.
Site	1	1	-0.1021	0.6074	-1.2926	1.0885	0.03	0.8666
Site	2	1	-0.5026	0.3583	-1.2049	0.1997	1.97	0.1607
Site	3	0	0.0000	0.0000	0.0000	0.0000	.	.
Highrisk5_sum	1	1	2.0169	0.8080	0.4333	3.6005	6.23	0.0126

Analysis Of Maximum Likelihood Parameter Estimates								
Parameter		DF	Estimate	Standard Error	Wald 95% Confidence Limits		Wald Chi-Square	Pr > ChiSq
Highrisk5_sum	2	1	0.9905	0.3867	0.2326	1.7484	6.56	0.0104
Highrisk5_sum	3	0	0.0000	0.0000	0.0000	0.0000	.	.
team_sum		1	-0.5816	0.1695	-0.9139	-0.2493	11.77	0.0006
Scale		0	1.0000	0.0000	1.0000	1.0000		

Note: The scale parameter was held fixed.

Wald Statistics For Type 3 Analysis			
Source	DF	Chi-Square	Pr > ChiSq
Revised_Groups	1	0.10	0.7530
Site	2	2.04	0.3601
Highrisk5_sum	2	11.04	0.0040
team_sum	1	11.77	0.0006

Contrast Estimate Results										
Label	Mean Estimate	Mean Confidence Limits		L'Beta Estimate	Standard Error	Alpha	L'Beta Confidence Limits		Chi-Square	Pr > ChiSq
team_sum	0.3586	0.2862	0.4380	-0.5816	0.1695	0.05	-0.9139	-0.2493	11.77	0.0006
Exp(team_sum)				0.5590	0.0948	0.05	0.4009	0.7793		

Revised_Groups Least Squares Means										
Revised_Groups	Estimate	Standard Error	z Value	Pr > |z|	Alpha	Lower	Upper	Exponentiated	Exponentiated Lower	Exponentiated Upper
Acquired	0.5765	0.3683	1.57	0.1175	0.05	-0.1453	1.2984	1.7798	0.8647	3.6634
Pre-existant	0.6833	0.3471	1.97	0.0490	0.05	0.003121	1.3635	1.9805	1.0031	3.9100

Differences of Revised_Groups Least Squares Means

Revised_Groups	_Revised_Groups	Estimate	Standard Error	z Value	Pr > |z|	Alpha	Lower	Upper	Exponentiated	Exponentiated Lower	Exponentiated Upper
Acquired	Pre-existant	-0.1068	0.3393	-0.31	0.7530	0.05	-0.7719	0.5583	0.8987	0.4621	1.7477

Site Least Squares Means

Site	Estimate	Standard Error	z Value	Pr > |z|	Alpha	Lower	Upper	Exponentiated	Exponentiated Lower	Exponentiated Upper
1	0.7294	0.5909	1.23	0.2171	0.05	-0.4288	1.8876	2.0739	0.6513	6.6035
2	0.3289	0.3469	0.95	0.3431	0.05	-0.3511	1.0088	1.3894	0.7039	2.7424
3	0.8315	0.3397	2.45	0.0144	0.05	0.1656	1.4973	2.2967	1.1801	4.4698

Differences of Site Least Squares Means

Site	_Site	Estimate	Standard Error	z Value	Pr > |z|	Alpha	Lower	Upper	Exponentiated	Exponentiated Lower	Exponentiated Upper
1	2	0.4005	0.6035	0.66	0.5069	0.05	-0.7823	1.5833	1.4926	0.4574	4.8712
1	3	-0.1021	0.6074	-0.17	0.8666	0.05	-1.2926	1.0885	0.9030	0.2746	2.9697
2	3	-0.5026	0.3583	-1.40	0.1607	0.05	-1.2049	0.1997	0.6050	0.2997	1.2210

Highrisk5_sum Least Squares Means

Highrisk5_sum	Estimate	Standard Error	z Value	Pr > |z|	Alpha	Lower	Upper	Exponentiated	Exponentiated Lower	Exponentiated Upper
1	1.6443	0.7825	2.10	0.0356	0.05	0.1107	3.1780	5.1776	1.1171	23.9976
2	0.6180	0.3558	1.74	0.0824	0.05	-0.07940	1.3153	1.8552	0.9237	3.7261
3	-0.3725	0.2466	-1.51	0.1308	0.05	-0.8558	0.1107	0.6890	0.4249	1.1171

Differences of Highrisk5_sum Least Squares Means

Highrisk5_sum	_Highrisk5_sum	Estimate	Standard Error	z Value	Pr > |z|	Alpha	Lower	Upper	Exponentiated	Exponentiated Lower	Exponentiated Upper
1	2	1.0264	0.8386	1.22	0.2210	0.05	-0.6173	2.6700	2.7909	0.5394	14.4405
1	3	2.0169	0.8080	2.50	0.0126	0.05	0.4333	3.6005	7.5149	1.5423	36.6173

Differences of Highrisk5_sum Least Squares Means											
Highrisk5_sum	*_Highrisk5_sum*	*Estimate*	*Standard Error*	*z Value*	*Pr > |z|*	*Alpha*	*Lower*	*Upper*	*Exponentiated*	*Exponentiated Lower*	*Exponentiated Upper*
2	3	0.9905	0.3867	2.56	0.0104	0.05	0.2326	1.7484	2.6926	1.2619	5.7455

Model 6 Final Multivariable Logistic regression of compromise_3Respiratory versus Group, site and confounders

The GENMOD Procedure

Model Information	
Data Set	WORK.CARDIAC2
Distribution	Binomial
Link Function	Logit
Dependent Variable	compromise_3Respiratory

Number of Observations Read	271
Number of Observations Used	174
Number of Events	169
Number of Trials	174
Missing Values	97

Class Level Information		
Class	*Levels*	*Values*
Revised_Groups	2	Acquired Pre-existant
Site	3	1 2 3

Response Profile		
Ordered Value	*compromise_3Respiratory*	*Total Frequency*
1	1	169

Response Profile		
Ordered Value	compromise_3Respiratory	Total Frequency
2	0	5

PROC GENMOD is modeling the probability that compromise_3Respiratory='1'.

Parameter Information			
Parameter	Effect	Revised_Groups	Site
Prm1	Intercept		
Prm2	Revised_Groups	Acquired	
Prm3	Revised_Groups	Pre-existant	
Prm4	Site		1
Prm5	Site		2
Prm6	Site		3
Prm7	Apgar_5min		

Criteria For Assessing Goodness Of Fit			
Criterion	DF	Value	Value/DF
Log Likelihood		-16.9642	
Full Log Likelihood		-16.9642	
AIC (smaller is better)		43.9284	
AICC (smaller is better)		44.2855	
BIC (smaller is better)		59.7237	

Algorithm converged.

Analysis Of Maximum Likelihood Parameter Estimates							
Parameter	DF	Estimate	Standard Error	Wald 95% Confidence Limits		Wald Chi-Square	Pr > ChiSq
Intercept	1	27.8560	9.8780	8.4955	47.2164	7.95	0.0048

Analysis Of Maximum Likelihood Parameter Estimates								
Parameter		*DF*	*Estimate*	*Standard Error*	*Wald 95% Confidence Limits*		*Wald Chi-Square*	*Pr > ChiSq*
Revised_Groups	Acquired	1	-1.9629	1.1649	-4.2462	0.3203	2.84	0.0920
Revised_Groups	Pre-existant	0	0.0000	0.0000	0.0000	0.0000	.	.
Site	1	1	22.9556	241272.5	-472863	472908.4	0.00	0.9999
Site	2	1	-0.5702	1.0486	-2.6255	1.4851	0.30	0.5866
Site	3	0	0.0000	0.0000	0.0000	0.0000	.	.
Apgar_5min		1	-2.4747	1.0059	-4.4463	-0.5031	6.05	0.0139
Scale		0	1.0000	0.0000	1.0000	1.0000		

Note: The scale parameter was held fixed.

Wald Statistics For Type 3 Analysis			
Source	*DF*	*Chi-Square*	*Pr > ChiSq*
Revised_Groups	1	2.84	0.0920
Site	2	0.30	0.8626
Apgar_5min	1	6.05	0.0139

Contrast Estimate Results										
Label	*Mean Estimate*	*Mean Confidence Limits*		*L'Beta Estimate*	*Standard Error*	*Alpha*	*L'Beta Confidence Limits*		*Chi-Square*	*Pr > ChiSq*
Apgar_5min	0.0777	0.0116	0.3768	-2.4747	1.0059	0.05	-4.4463	-0.5031	6.05	0.0139
Exp(Apgar_5min)				0.0842	0.0847	0.05	0.0117	0.6046		

Revised_Groups Least Squares Means										
Revised_Groups	*Estimate*	*Standard Error*	*z Value*	*Pr > |z|*	*Alpha*	*Lower*	*Upper*	*Exponentiated*	*Exponentiated Lower*	*Exponentiated Upper*
Acquired	11.6656	80424	0.00	0.9999	0.05	-157617	157640	116496	0	Infty
Pre-existant	13.6285	80424	0.00	0.9999	0.05	-157615	157642	829462	0	Infty

Differences of Revised_Groups Least Squares Means

Revised_Groups	*_Revised_Groups*	*Estimate*	*Standard Error*	*z Value*	*Pr > |z|*	*Alpha*	*Lower*	*Upper*	*Exponentiated*	*Exponentiated Lower*	*Exponentiated Upper*
Acquired	Pre-existant	-1.9629	1.1649	-1.68	0.0920	0.05	-4.2462	0.3203	0.1404	0.01432	1.3776

Site Least Squares Means

Site	*Estimate*	*Standard Error*	*z Value*	*Pr > |z|*	*Alpha*	*Lower*	*Upper*	*Exponentiated*	*Exponentiated Lower*	*Exponentiated Upper*
1	28.1409	241273	0.00	0.9999	0.05	-472857	472914	1.665E12	0	Infty
2	4.6151	1.0195	4.53	<.0001	0.05	2.6168	6.6133	101.00	13.6920	744.96
3	5.1853	1.1844	4.38	<.0001	0.05	2.8639	7.5066	178.62	17.5295	1820.06

Differences of Site Least Squares Means

Site	*_Site*	*Estimate*	*Standard Error*	*z Value*	*Pr > |z|*	*Alpha*	*Lower*	*Upper*	*Exponentiated*	*Exponentiated Lower*	*Exponentiated Upper*
1	2	23.5258	241273	0.00	0.9999	0.05	-472862	472909	1.649E10	0	Infty
1	3	22.9556	241273	0.00	0.9999	0.05	-472863	472908	9.3219E9	0	Infty
2	3	-0.5702	1.0486	-0.54	0.5866	0.05	-2.6255	1.4851	0.5654	0.07241	4.4154

Model 22 Final Multivariable Ordinal logistic regression of compromise_sum versus Group, site and confounders

The GENMOD Procedure

Model Information

Data Set	WORK.CARDIAC2
Distribution	Multinomial
Link Function	Cumulative Logit
Dependent Variable	compromise_sum

Number of Observations Read	271
Number of Observations Used	176
Missing Values	95

Class Level Information		
Class	Levels	Values
Revised_Groups	2	Acquired Pre-existant
Site	3	1 2 3

Response Profile		
Ordered Value	compromise_sum	Total Frequency
1	4	163
2	3	9
3	2	1
4	1	3

PROC GENMOD is modeling the probabilities of levels of compromise_sum having LOWER Ordered Values in the response profile table.

Parameter Information			
Parameter	Effect	Revised_Groups	Site
Prm1	Revised_Groups	Acquired	
Prm2	Revised_Groups	Pre-existant	
Prm3	Site		1
Prm4	Site		2
Prm5	Site		3

Criteria For Assessing Goodness Of Fit			
Criterion	DF	Value	Value/DF
Log Likelihood		-55.5528	

Criteria For Assessing Goodness Of Fit			
Criterion	*DF*	*Value*	*Value/DF*
Full Log Likelihood		-55.5528	
AIC (smaller is better)		123.1055	
AICC (smaller is better)		123.6026	
BIC (smaller is better)		142.1284	

Algorithm converged.

Analysis Of Maximum Likelihood Parameter Estimates								
Parameter		*DF*	*Estimate*	*Standard Error*	*Wald 95% Confidence Limits*		*Wald Chi-Square*	*Pr > ChiSq*
Intercept1		1	2.3725	0.5279	1.3378	3.4071	20.20	<.0001
Intercept2		1	3.6108	0.6705	2.2966	4.9250	29.00	<.0001
Intercept3		1	3.9052	0.7310	2.4725	5.3379	28.54	<.0001
Revised_Groups	Acquired	1	-0.1148	0.6012	-1.2932	1.0636	0.04	0.8486
Revised_Groups	Pre-existant	0	0.0000	0.0000	0.0000	0.0000	.	.
Site	1	1	23.3251	107108.8	-209906	209952.7	0.00	0.9998
Site	2	1	0.2615	0.6004	-0.9152	1.4383	0.19	0.6631
Site	3	0	0.0000	0.0000	0.0000	0.0000	.	.
Scale		0	1.0000	0.0000	1.0000	1.0000		

Note: The scale parameter was held fixed.

Wald Statistics For Type 3 Analysis			
Source	*DF*	*Chi-Square*	*Pr > ChiSq*
Revised_Groups	1	0.04	0.8486
Site	2	0.19	0.9095

Revised_Groups Least Squares Means

compromise_sum	*Revised_Groups*	*Estimate*	*Standard Error*	*z Value*	*Pr > |z|*	*Alpha*	*Lower*	*Upper*	*Exponentiated*	*Exponentiated Lower*	*Exponentiated Upper*
4	Acquired	10.1199	35703	0.00	0.9998	0.05	-69966	69987	24832	0	Infty
4	Pre-existant	10.2347	35703	0.00	0.9998	0.05	-69966	69987	27853	0	Infty
3	Acquired	11.3582	35703	0.00	0.9997	0.05	-69965	69988	85669	0	Infty
3	Pre-existant	11.4730	35703	0.00	0.9997	0.05	-69965	69988	96090	0	Infty
2	Acquired	11.6526	35703	0.00	0.9997	0.05	-69965	69988	114991	0	Infty
2	Pre-existant	11.7674	35703	0.00	0.9997	0.05	-69965	69988	128979	0	Infty

Differences of Revised_Groups Least Squares Means

Revised_Groups	*_Revised_Groups*	*Estimate*	*Standard Error*	*z Value*	*Pr > |z|*	*Alpha*	*Lower*	*Upper*	*Exponentiated*	*Exponentiated Lower*	*Exponentiated Upper*
Acquired	Pre-existant	-0.1148	0.6012	-0.19	0.8486	0.05	-1.2932	1.0636	0.8915	0.2744	2.8967

Site Least Squares Means

compromise_sum	*Site*	*Estimate*	*Standard Error*	*z Value*	*Pr > |z|*	*Alpha*	*Lower*	*Upper*	*Exponentiated*	*Exponentiated Lower*	*Exponentiated Upper*
4	1	25.6402	107109	0.00	0.9998	0.05	-209904	209955	1.366E11	0	Infty
4	2	2.5766	0.4369	5.90	<.0001	0.05	1.7203	3.4330	13.1527	5.5861	30.9687
4	3	2.3151	0.3991	5.80	<.0001	0.05	1.5328	3.0974	10.1257	4.6311	22.1395
3	1	26.8785	107109	0.00	0.9998	0.05	-209902	209956	4.712E11	0	Infty
3	2	3.8150	0.6048	6.31	<.0001	0.05	2.6297	5.0003	45.3760	13.8694	148.46
3	3	3.5534	0.5746	6.18	<.0001	0.05	2.4273	4.6796	34.9331	11.3280	107.73
2	1	27.1729	107109	0.00	0.9998	0.05	-209902	209957	6.325E11	0	Infty
2	2	4.1093	0.6698	6.14	<.0001	0.05	2.7966	5.4221	60.9070	16.3885	226.36
2	3	3.8478	0.6431	5.98	<.0001	0.05	2.5873	5.1083	46.8898	13.2941	165.39

Differences of Site Least Squares Means											
Site	_Site	Estimate	Standard Error	z Value	Pr > |z|	Alpha	Lower	Upper	Exponentiated	Exponentiated Lower	Exponentiated Upper
1	2	23.0635	107109	0.00	0.9998	0.05	-209906	209952	1.038E10	0	Infty
1	3	23.3251	107109	0.00	0.9998	0.05	-209906	209953	1.349E10	0	Infty
2	3	0.2615	0.6004	0.44	0.6631	0.05	-0.9152	1.4383	1.2989	0.4004	4.2136

Model 7 Final Multivariable Logistic regression of compromise_4Changing versus Group, site and confounders

The GENMOD Procedure

Model Information	
Data Set	WORK.CARDIAC2
Distribution	Binomial
Link Function	Logit
Dependent Variable	compromise_4Changing

Number of Observations Read	271
Number of Observations Used	176
Number of Events	165
Number of Trials	176
Missing Values	95

Class Level Information		
Class	Levels	Values
Revised_Groups	2	Acquired Pre-existant
Site	3	1 2 3

Response Profile		
Ordered Value	compromise_4Changing	Total Frequency
1	1	165
2	0	11

PROC GENMOD is modeling the probability that compromise_4Changing='1'.

Parameter Information			
Parameter	Effect	Revised_Groups	Site
Prm1	Intercept		
Prm2	Revised_Groups	Acquired	
Prm3	Revised_Groups	Pre-existant	
Prm4	Site		1
Prm5	Site		2
Prm6	Site		3

Criteria For Assessing Goodness Of Fit			
Criterion	DF	Value	Value/DF
Log Likelihood		-40.2079	
Full Log Likelihood		-40.2079	
AIC (smaller is better)		88.4159	
AICC (smaller is better)		88.6498	
BIC (smaller is better)		101.0978	

Algorithm converged.

Analysis Of Maximum Likelihood Parameter Estimates							
Parameter	DF	Estimate	Standard Error	Wald 95% Confidence Limits		Wald Chi-Square	Pr > ChiSq
Intercept	1	2.4757	0.5654	1.3676	3.5838	19.17	<.0001

Analysis Of Maximum Likelihood Parameter Estimates								
Parameter		*DF*	*Estimate*	*Standard Error*	*Wald 95% Confidence Limits*		*Wald Chi-Square*	*Pr > ChiSq*
Revised_Groups	Acquired	1	-0.0083	0.6503	-1.2828	1.2662	0.00	0.9898
Revised_Groups	Pre-existant	0	0.0000	0.0000	0.0000	0.0000	.	.
Site	1	1	23.8931	153307.3	-300453	300500.7	0.00	0.9999
Site	2	1	0.3242	0.6495	-0.9487	1.5971	0.25	0.6177
Site	3	0	0.0000	0.0000	0.0000	0.0000	.	.
Scale		0	1.0000	0.0000	1.0000	1.0000		

Note: The scale parameter was held fixed.

Wald Statistics For Type 3 Analysis			
Source	*DF*	*Chi-Square*	*Pr > ChiSq*
Revised_Groups	1	0.00	0.9898
Site	2	0.25	0.8829

Revised_Groups Least Squares Means

Revised_Groups	*Estimate*	*Standard Error*	*z Value*	*Pr > |z|*	*Alpha*	*Lower*	*Upper*	*Exponentiated*	*Exponentiated Lower*	*Exponentiated Upper*
Acquired	10.5398	51102	0.00	0.9998	0.05	-100148	100169	37789	0	Infty
Pre-existant	10.5481	51102	0.00	0.9998	0.05	-100148	100169	38106	0	Infty

Differences of Revised_Groups Least Squares Means

Revised_Groups	*_Revised_Groups*	*Estimate*	*Standard Error*	*z Value*	*Pr > |z|*	*Alpha*	*Lower*	*Upper*	*Exponentiated*	*Exponentiated Lower*	*Exponentiated Upper*
Acquired	Pre-existant	-0.00833	0.6503	-0.01	0.9898	0.05	-1.2828	1.2662	0.9917	0.2773	3.5472

Site Least Squares Means										
Site	Estimate	Standard Error	z Value	Pr > |z|	Alpha	Lower	Upper	Exponentiated	Exponentiated Lower	Exponentiated Upper
1	26.3646	153307	0.00	0.9999	0.05	-300450	300503	2.818E11	0	Infty
2	2.7957	0.4766	5.87	<.0001	0.05	1.8616	3.7298	16.3742	6.4339	41.6722
3	2.4715	0.4278	5.78	<.0001	0.05	1.6330	3.3100	11.8405	5.1195	27.3851

Differences of Site Least Squares Means											
Site	_Site	Estimate	Standard Error	z Value	Pr > |z|	Alpha	Lower	Upper	Exponentiated	Exponentiated Lower	Exponentiated Upper
1	2	23.5689	153307	0.00	0.9999	0.05	-300453	300500	1.721E10	0	Infty
1	3	23.8931	153307	0.00	0.9999	0.05	-300453	300501	2.38E10	0	Infty
2	3	0.3242	0.6495	0.50	0.6177	0.05	-0.9487	1.5971	1.3829	0.3872	4.9387

Model 8 Final Multivariable Logistic regression of thromboembolism_sum versus Group, site and confounders

The GENMOD Procedure

Model Information	
Data Set	WORK.CARDIAC2
Distribution	Binomial
Link Function	Logit
Dependent Variable	thromboembolism_sum

Number of Observations Read	271
Number of Observations Used	118
Number of Events	59
Number of Trials	118
Missing Values	153

Class Level Information		
Class	Levels	Values
Revised_Groups	2	Acquired Pre-existant
Site	3	1 2 3
team_5Anaesthetist	2	0 1

Response Profile		
Ordered Value	thromboembolism_sum	Total Frequency
1	2	59
2	1	59

PROC GENMOD is modeling the probability that thromboembolism_sum='2'.

Parameter Information				
Parameter	Effect	Revised_Groups	Site	team_5Anaesthetist
Prm1	Intercept			
Prm2	Revised_Groups	Acquired		
Prm3	Revised_Groups	Pre-existant		
Prm4	Site		1	
Prm5	Site		2	
Prm6	Site		3	
Prm7	team_5Anaesthetist			0
Prm8	team_5Anaesthetist			1

Criteria For Assessing Goodness Of Fit			
Criterion	DF	Value	Value/DF
Log Likelihood		-72.4554	
Full Log Likelihood		-72.4554	
AIC (smaller is better)		154.9108	
AICC (smaller is better)		155.4465	

Criteria For Assessing Goodness Of Fit			
Criterion	*DF*	*Value*	*Value/DF*
BIC (smaller is better)		168.7642	

Algorithm converged.

Analysis Of Maximum Likelihood Parameter Estimates									
Parameter		*DF*	*Estimate*	*Standard Error*	*Wald 95% Confidence Limits*		*Wald Chi-Square*	*Pr > ChiSq*
Intercept		1	0.0526	0.4407	-0.8112	0.9165	0.01	0.9049
Revised_Groups	Acquired	1	-0.5391	0.4189	-1.3602	0.2820	1.66	0.1981
Revised_Groups	Pre-existant	0	0.0000	0.0000	0.0000	0.0000	.	.
Site	1	1	-0.9850	0.8823	-2.7142	0.7443	1.25	0.2642
Site	2	1	0.7058	0.4506	-0.1774	1.5889	2.45	0.1173
Site	3	0	0.0000	0.0000	0.0000	0.0000	.	.
team_5Anaesthetist	0	1	-1.6782	0.8128	-3.2713	-0.0851	4.26	0.0390
team_5Anaesthetist	1	0	0.0000	0.0000	0.0000	0.0000	.	.
Scale		0	1.0000	0.0000	1.0000	1.0000		

Note: The scale parameter was held fixed.

Wald Statistics For Type 3 Analysis			
Source	*DF*	*Chi-Square*	*Pr > ChiSq*
Revised_Groups	1	1.66	0.1981
Site	2	5.45	0.0656
team_5Anaesthetist	1	4.26	0.0390

Revised_Groups Least Squares Means

Revised_Groups	Estimate	Standard Error	z Value	Pr > |z|	Alpha	Lower	Upper	Exponentiated	Exponentiated Lower	Exponentiated Upper
Acquired	-1.4186	0.5026	-2.82	0.0048	0.05	-2.4037	-0.4336	0.2420	0.09039	0.6482
Pre-existant	-0.8795	0.5141	-1.71	0.0871	0.05	-1.8871	0.1281	0.4150	0.1515	1.1366

Differences of Revised_Groups Least Squares Means

Revised_Groups	_Revised_Groups	Estimate	Standard Error	z Value	Pr > |z|	Alpha	Lower	Upper	Exponentiated	Exponentiated Lower	Exponentiated Upper
Acquired	Pre-existant	-0.5391	0.4189	-1.29	0.1981	0.05	-1.3602	0.2820	0.5833	0.2566	1.3257

Site Least Squares Means

Site	Estimate	Standard Error	z Value	Pr > |z|	Alpha	Lower	Upper	Exponentiated	Exponentiated Lower	Exponentiated Upper
1	-2.0410	0.8768	-2.33	0.0199	0.05	-3.7594	-0.3226	0.1299	0.02330	0.7243
2	-0.3502	0.4430	-0.79	0.4292	0.05	-1.2185	0.5180	0.7045	0.2957	1.6787
3	-1.0560	0.4955	-2.13	0.0331	0.05	-2.0271	-0.08493	0.3478	0.1317	0.9186

Differences of Site Least Squares Means

Site	_Site	Estimate	Standard Error	z Value	Pr > |z|	Alpha	Lower	Upper	Exponentiated	Exponentiated Lower	Exponentiated Upper
1	2	-1.6908	0.8478	-1.99	0.0461	0.05	-3.3525	-0.02902	0.1844	0.03500	0.9714
1	3	-0.9850	0.8823	-1.12	0.2642	0.05	-2.7142	0.7443	0.3734	0.06626	2.1049
2	3	0.7058	0.4506	1.57	0.1173	0.05	-0.1774	1.5889	2.0254	0.8374	4.8986

team_5Anaesthetist Least Squares Means

team_5Anaesthetist	Estimate	Standard Error	z Value	Pr > |z|	Alpha	Lower	Upper	Exponentiated	Exponentiated Lower	Exponentiated Upper
0	-1.9882	0.8141	-2.44	0.0146	0.05	-3.5838	-0.3925	0.1369	0.02777	0.6753
1	-0.3100	0.3109	-1.00	0.3187	0.05	-0.9193	0.2993	0.7335	0.3988	1.3489

Differences of team_5Anaesthetist Least Squares Means												
team_5Anaesthetist	_team_5Anaesthetist	Estimate	Standard Error	z Value	Pr > |z|	Alpha	Lower	Upper	Exponentiated	Exponentiated Lower	Exponentiated Upper
0	1	-1.6782	0.8128	-2.06	0.0390	0.05	-3.2713	-0.08512	0.1867	0.03796	0.9184

Model 9 Final Multivariable Logistic regression of thromboembolism_1stockings versus Group, site and confounders

The GENMOD Procedure

Model Information	
Data Set	WORK.CARDIAC2
Distribution	Binomial
Link Function	Logit
Dependent Variable	thromboembolism_1stockings

Number of Observations Read	271
Number of Observations Used	115
Number of Events	74
Number of Trials	115
Missing Values	156

Class Level Information		
Class	Levels	Values
Revised_Groups	2	Acquired Pre-existant
Site	3	1 2 3

Response Profile		
Ordered Value	thromboembolism_1stockings	Total Frequency
1	1	74

Response Profile		
Ordered Value	thromboembolism_1stockings	Total Frequency
2	0	41

PROC GENMOD is modeling the probability that thromboembolism_1stockings='1'.

Parameter Information			
Parameter	Effect	Revised_Groups	Site
Prm1	Intercept		
Prm2	Revised_Groups	Acquired	
Prm3	Revised_Groups	Pre-existant	
Prm4	Site		1
Prm5	Site		2
Prm6	Site		3
Prm7	Live_baby_weight		

Criteria For Assessing Goodness Of Fit			
Criterion	DF	Value	Value/DF
Log Likelihood		-68.6009	
Full Log Likelihood		-68.6009	
AIC (smaller is better)		147.2019	
AICC (smaller is better)		147.7523	
BIC (smaller is better)		160.9265	

Algorithm converged.

Analysis Of Maximum Likelihood Parameter Estimates								
Parameter		*DF*	*Estimate*	*Standard Error*	*Wald 95% Confidence Limits*		*Wald Chi-Square*	*Pr > ChiSq*
Intercept		1	2.8485	0.9684	0.9504	4.7466	8.65	0.0033
Revised_Groups	Acquired	1	-0.2226	0.4387	-1.0825	0.6372	0.26	0.6118
Revised_Groups	Pre-existant	0	0.0000	0.0000	0.0000	0.0000	.	.
Site	1	1	-1.3663	0.7259	-2.7891	0.0564	3.54	0.0598
Site	2	1	0.0658	0.4763	-0.8678	0.9995	0.02	0.8901
Site	3	0	0.0000	0.0000	0.0000	0.0000	.	.
Live_baby_weight		1	-0.0007	0.0003	-0.0012	-0.0001	5.68	0.0171
Scale		0	1.0000	0.0000	1.0000	1.0000		

Note: The scale parameter was held fixed.

Wald Statistics For Type 3 Analysis			
Source	*DF*	*Chi-Square*	*Pr > ChiSq*
Revised_Groups	1	0.26	0.6118
Site	2	4.42	0.1098
Live_baby_weight	1	5.68	0.0171

Contrast Estimate Results										
Label	*Mean Estimate*	*Mean Confidence Limits*		*L'Beta Estimate*	*Standard Error*	*Alpha*	*L'Beta Confidence Limits*		*Chi-Square*	*Pr > ChiSq*
Live_baby_weight per 1kg increase	0.3393	0.2290	0.4704	-0.6662	0.2795	0.05	-1.2140	-0.1184	5.68	0.0171
Exp(Live_baby_weight per 1kg increase)				0.5137	0.1436	0.05	0.2970	0.8883		

Revised_Groups	Estimate	Standard Error	z Value	Pr > |z|	Alpha	Lower	Upper	Exponentiated	Exponentiated Lower	Exponentiated Upper
Acquired	0.1993	0.3317	0.60	0.5479	0.05	-0.4508	0.8495	1.2206	0.6371	2.3384
Pre-existant	0.4219	0.3489	1.21	0.2265	0.05	-0.2619	1.1058	1.5249	0.7696	3.0215

Revised_Groups Least Squares Means

Differences of Revised_Groups Least Squares Means

Revised_Groups	_Revised_Groups	Estimate	Standard Error	z Value	Pr > |z|	Alpha	Lower	Upper	Exponentiated	Exponentiated Lower	Exponentiated Upper
Acquired	Pre-existant	-0.2226	0.4387	-0.51	0.6118	0.05	-1.0825	0.6372	0.8004	0.3388	1.8912

Site Least Squares Means

Site	Estimate	Standard Error	z Value	Pr > |z|	Alpha	Lower	Upper	Exponentiated	Exponentiated Lower	Exponentiated Upper
1	-0.6222	0.6281	-0.99	0.3219	0.05	-1.8533	0.6089	0.5368	0.1567	1.8384
2	0.8100	0.2864	2.83	0.0047	0.05	0.2487	1.3713	2.2479	1.2823	3.9404
3	0.7441	0.3748	1.99	0.0471	0.05	0.009592	1.4787	2.1046	1.0096	4.3871

Differences of Site Least Squares Means

Site	_Site	Estimate	Standard Error	z Value	Pr > |z|	Alpha	Lower	Upper	Exponentiated	Exponentiated Lower	Exponentiated Upper
1	2	-1.4322	0.6981	-2.05	0.0402	0.05	-2.8004	-0.06393	0.2388	0.06078	0.9381
1	3	-1.3663	0.7259	-1.88	0.0598	0.05	-2.7891	0.05639	0.2550	0.06148	1.0580
2	3	0.06585	0.4763	0.14	0.8901	0.05	-0.8678	0.9995	1.0681	0.4199	2.7168

Model 10 Final Multivariable Logistic regression of thromboembolism_2heparin versus Group, site and confounders

The GENMOD Procedure

Model Information

Data Set	WORK.CARDIAC2

Model Information	
Distribution	Binomial
Link Function	Logit
Dependent Variable	thromboembolism_2heparin

Number of Observations Read	271
Number of Observations Used	75
Number of Events	67
Number of Trials	75
Missing Values	196

Class Level Information

Class	*Levels*	*Values*
Revised_Groups	2	Acquired Pre-existant
Site	3	1 2 3
Cardiac_consultation	2	1.Yes 2.No

Response Profile

Ordered Value	*thromboembolism_2heparin*	*Total Frequency*
1	1	67
2	0	8

PROC GENMOD is modeling the probability that thromboembolism_2heparin='1'.

Parameter Information

Parameter	*Effect*	*Revised_Groups*	*Site*	*Cardiac_consultation*
Prm1	Intercept			
Prm2	Revised_Groups	Acquired		
Prm3	Revised_Groups	Pre-existant		

Parameter Information				
Parameter	*Effect*	*Revised_Groups*	*Site*	*Cardiac_consultation*
Prm4	Site		1	
Prm5	Site		2	
Prm6	Site		3	
Prm7	Cardiac_consultation			1.Yes
Prm8	Cardiac_consultation			2.No
Prm9	Parity			

Criteria For Assessing Goodness Of Fit			
Criterion	*DF*	*Value*	*Value/DF*
Log Likelihood		-13.9821	
Full Log Likelihood		-13.9821	
AIC (smaller is better)		39.9642	
AICC (smaller is better)		41.1995	
BIC (smaller is better)		53.8692	

Algorithm converged.

Analysis Of Maximum Likelihood Parameter Estimates								
Parameter		*DF*	*Estimate*	*Standard Error*	*Wald 95% Confidence Limits*		*Wald Chi-Square*	*Pr > ChiSq*
Intercept		1	2.8790	1.8895	-0.8243	6.5824	2.32	0.1276
Revised_Groups	Acquired	1	-3.4841	1.5998	-6.6198	-0.3485	4.74	0.0294
Revised_Groups	Pre-existant	0	0.0000	0.0000	0.0000	0.0000	.	.
Site	1	1	26.7358	295367.2	-578882	578935.9	0.00	0.9999
Site	2	1	-0.5984	1.1270	-2.8074	1.6105	0.28	0.5954
Site	3	0	0.0000	0.0000	0.0000	0.0000	.	.
Cardiac_consultation	1.Yes	1	4.1792	1.4730	1.2922	7.0662	8.05	0.0046
Cardiac_consultation	2.No	0	0.0000	0.0000	0.0000	0.0000	.	.

Analysis Of Maximum Likelihood Parameter Estimates							
Parameter	DF	Estimate	Standard Error	Wald 95% Confidence Limits		Wald Chi-Square	Pr > ChiSq
Parity	1	-0.8501	0.3437	-1.5236	-0.1765	6.12	0.0134
Scale	0	1.0000	0.0000	1.0000	1.0000		

Note: The scale parameter was held fixed.

Wald Statistics For Type 3 Analysis			
Source	DF	Chi-Square	Pr > ChiSq
Revised_Groups	1	4.74	0.0294
Site	2	0.28	0.8685
Cardiac_consultation	1	8.05	0.0046
Parity	1	6.12	0.0134

Contrast Estimate Results										
Label	Mean Estimate	Mean Confidence Limits		L'Beta Estimate	Standard Error	Alpha	L'Beta Confidence Limits		Chi-Square	Pr > ChiSq
Parity	0.2994	0.1789	0.4560	-0.8501	0.3437	0.05	-1.5236	-0.1765	6.12	0.0134
Exp(Parity)				0.4274	0.1469	0.05	0.2179	0.8382		

Revised_Groups Least Squares Means										
Revised_Groups	Estimate	Standard Error	z Value	Pr > |z|	Alpha	Lower	Upper	Exponentiated	Exponentiated Lower	Exponentiated Upper
Acquired	8.8709	98456	0.00	0.9999	0.05	-192961	192979	7121.39	0	Infty
Pre-existant	12.3550	98456	0.00	0.9999	0.05	-192957	192982	232118	0	Infty

Differences of Revised_Groups Least Squares Means											
Revised_Groups	_Revised_Groups	Estimate	Standard Error	z Value	Pr > |z|	Alpha	Lower	Upper	Exponentiated	Exponentiated Lower	Exponentiated Upper
Acquired	Pre-existant	-3.4841	1.5998	-2.18	0.0294	0.05	-6.6198	-0.3485	0.03068	0.001334	0.7057

Site Least Squares Means										
Site	Estimate	Standard Error	z Value	Pr > |z|	Alpha	Lower	Upper	Exponentiated	Exponentiated Lower	Exponentiated Upper
1	28.6363	295367	0.00	0.9999	0.05	-578880	578938	2.733E12	0	Infty
2	1.3020	0.7441	1.75	0.0802	0.05	-0.1564	2.7605	3.6768	0.8552	15.8075
3	1.9005	1.0369	1.83	0.0668	0.05	-0.1317	3.9327	6.6891	0.8766	51.0435

Differences of Site Least Squares Means											
Site	_Site	Estimate	Standard Error	z Value	Pr > |z|	Alpha	Lower	Upper	Exponentiated	Exponentiated Lower	Exponentiated Upper
1	2	27.3342	295367	0.00	0.9999	0.05	-578882	578936	7.432E11	0	Infty
1	3	26.7358	295367	0.00	0.9999	0.05	-578882	578936	4.085E11	0	Infty
2	3	-0.5984	1.1270	-0.53	0.5954	0.05	-2.8074	1.6105	0.5497	0.06036	5.0055

Cardiac_consultation Least Squares Means										
Cardiac_consultationx	Estimate	Standard Error	z Value	Pr > |z|	Alpha	Lower	Upper	Exponentiated	Exponentiated Lower	Exponentiated Upper
1.Yes	12.7025	98456	0.00	0.9999	0.05	-192957	192982	328582	0	Infty
2.No	8.5233	98456	0.00	0.9999	0.05	-192961	192978	5030.72	0	Infty

Differences of Cardiac_consultation Least Squares Means											
Cardiac_consultationx	_Cardiac_consultationx	Estimate	Standard Error	z Value	Pr > |z|	Alpha	Lower	Upper	Exponentiated	Exponentiated Lower	Exponentiated Upper
1.Yes	2.No	4.1792	1.4730	2.84	0.0046	0.05	1.2922	7.0662	65.3152	3.6409	1171.72

Model 23 Final Multivariable Ordinal logistic regression of Pain_sum versus Group, site and confounders

The GENMOD Procedure

Model Information	
Data Set	WORK.CARDIAC2
Distribution	Multinomial
Link Function	Cumulative Logit
Dependent Variable	Pain_sum

Number of Observations Read	271
Number of Observations Used	204
Missing Values	67

Class Level Information		
Class	*Levels*	*Values*
Revised_Groups	2	Acquired Pre-existant
Site	3	1 2 3
team_1Obstetricianx	2	1.Yes 2.No
team_5Anaesthetistx	2	1.Yes 2.No

Response Profile		
Ordered Value	*Pain_sum*	*Total Frequency*
1	4	28
2	3	40
3	2	73
4	1	63

PROC GENMOD is modeling the probabilities of levels of Pain_sum having LOWER Ordered Values in the response profile table.

Parameter Information					
Parameter	*Effect*	*Revised_Groups*	*Site*	*team_1Obstetricianx*	*team_5Anaesthetistx*
Prm1	Revised_Groups	Acquired			
Prm2	Revised_Groups	Pre-existant			
Prm3	Site		1		
Prm4	Site		2		
Prm5	Site		3		
Prm6	team_1Obstetricianx			1.Yes	
Prm7	team_1Obstetricianx			2.No	
Prm8	team_5Anaesthetistx				1.Yes
Prm9	team_5Anaesthetistx				2.No
Prm10	Live_baby_weight				

Criteria For Assessing Goodness Of Fit			
Criterion	*DF*	*Value*	*Value/DF*
Log Likelihood		-241.6578	
Full Log Likelihood		-241.6578	
AIC (smaller is better)		501.3155	
AICC (smaller is better)		502.2433	
BIC (smaller is better)		531.1786	

Algorithm converged.

Analysis Of Maximum Likelihood Parameter Estimates							
Parameter	*DF*	*Estimate*	*Standard Error*	*Wald 95% Confidence Limits*		*Wald Chi-Square*	*Pr > ChiSq*
Intercept1	1	-0.5695	0.7391	-2.0181	0.8792	0.59	0.4410
Intercept2	1	0.7420	0.7447	-0.7177	2.2016	0.99	0.3191
Intercept3	1	2.5696	0.7662	1.0679	4.0713	11.25	0.0008

Analysis Of Maximum Likelihood Parameter Estimates								
Parameter		DF	Estimate	Standard Error	Wald 95% Confidence Limits		Wald Chi-Square	Pr > ChiSq
Revised_Groups	Acquired	1	-0.9690	0.2879	-1.5333	-0.4047	11.33	0.0008
Revised_Groups	Pre-existant	0	0.0000	0.0000	0.0000	0.0000	.	.
Site	1	1	-0.6022	0.4734	-1.5299	0.3256	1.62	0.2033
Site	2	1	-0.7536	0.3078	-1.3569	-0.1503	5.99	0.0144
Site	3	0	0.0000	0.0000	0.0000	0.0000	.	.
team_1Obstetricianx	1.Yes	1	1.0321	0.3595	0.3274	1.7367	8.24	0.0041
team_1Obstetricianx	2.No	0	0.0000	0.0000	0.0000	0.0000	.	.
team_5Anaesthetistx	1.Yes	1	1.0374	0.4439	0.1674	1.9073	5.46	0.0194
team_5Anaesthetistx	2.No	0	0.0000	0.0000	0.0000	0.0000	.	.
Live_baby_weight		1	-0.0008	0.0002	-0.0012	-0.0004	17.66	<.0001
Scale		0	1.0000	0.0000	1.0000	1.0000		

Note: The scale parameter was held fixed.

Wald Statistics For Type 3 Analysis			
Source	DF	Chi-Square	Pr > ChiSq
Revised_Groups	1	11.33	0.0008
Site	2	6.36	0.0416
team_1Obstetricianx	1	8.24	0.0041
team_5Anaesthetistx	1	5.46	0.0194
Live_baby_weight	1	17.66	<.0001

Contrast Estimate Results										
Label	Mean Estimate	Mean Confidence Limits		L'Beta Estimate	Standard Error	Alpha	L'Beta Confidence Limits		Chi-Square	Pr > ChiSq
Live_baby_weight per 1kg increase	0.3132	0.2403	0.3968	-0.7850	0.1868	0.05	-1.1511	-0.4188	17.66	<.0001

Contrast Estimate Results										
		Mean					L'Beta			
Label	Mean Estimate	Confidence Limits		L'Beta Estimate	Standard Error	Alpha	Confidence Limits		Chi-Square	Pr > ChiSq
Exp(Live_baby_weight per 1kg increase)				0.4561	0.0852	0.05	0.3163	0.6578		

Revised_Groups Least Squares Means

Pain_sum	Revised_Groups	Estimate	Standard Error	z Value	Pr > |z|	Alpha	Lower	Upper	Exponentiated	Exponentiated Lower	Exponentiated Upper
4	Acquired	-3.3519	0.3641	-9.21	<.0001	0.05	-4.0655	-2.6383	0.03502	0.01715	0.07148
4	Pre-existant	-2.3829	0.3130	-7.61	<.0001	0.05	-2.9964	-1.7695	0.09228	0.04997	0.1704
3	Acquired	-2.0405	0.3173	-6.43	<.0001	0.05	-2.6624	-1.4186	0.1300	0.06978	0.2421
3	Pre-existant	-1.0715	0.2787	-3.85	0.0001	0.05	-1.6177	-0.5253	0.3425	0.1984	0.5914
2	Acquired	-0.2129	0.2833	-0.75	0.4524	0.05	-0.7681	0.3424	0.8083	0.4639	1.4083
2	Pre-existant	0.7561	0.2792	2.71	0.0068	0.05	0.2089	1.3034	2.1300	1.2323	3.6818

Differences of Revised_Groups Least Squares Means

Revised_Groups	_Revised_Groups	Estimate	Standard Error	z Value	Pr > |z|	Alpha	Lower	Upper	Exponentiated	Exponentiated Lower	Exponentiated Upper
Acquired	Pre-existant	-0.9690	0.2879	-3.37	0.0008	0.05	-1.5333	-0.4047	0.3795	0.2158	0.6672

Site Least Squares Means

Pain_sum	Site	Estimate	Standard Error	z Value	Pr > |z|	Alpha	Lower	Upper	Exponentiated	Exponentiated Lower	Exponentiated Upper
4	1	-3.0177	0.4962	-6.08	<.0001	0.05	-3.9902	-2.0451	0.04892	0.01850	0.1294
4	2	-3.1691	0.3520	-9.00	<.0001	0.05	-3.8591	-2.4792	0.04204	0.02109	0.08381
4	3	-2.4155	0.3139	-7.70	<.0001	0.05	-3.0307	-1.8003	0.08932	0.04828	0.1653
3	1	-1.7062	0.4689	-3.64	0.0003	0.05	-2.6252	-0.7872	0.1815	0.07242	0.4551
3	2	-1.8577	0.3058	-6.08	<.0001	0.05	-2.4569	-1.2584	0.1560	0.08570	0.2841
3	3	-1.1041	0.2777	-3.98	<.0001	0.05	-1.6483	-0.5599	0.3315	0.1924	0.5713
2	1	0.1214	0.4565	0.27	0.7903	0.05	-0.7734	1.0162	1.1291	0.4614	2.7626

Site Least Squares Means

Pain_sum	*Site*	*Estimate*	*Standard Error*	*z Value*	*Pr > |z|*	*Alpha*	*Lower*	*Upper*	*Exponentiated*	*Exponentiated Lower*	*Exponentiated Upper*
2	2	-0.03006	0.2772	-0.11	0.9137	0.05	-0.5733	0.5132	0.9704	0.5636	1.6707
2	3	0.7235	0.2739	2.64	0.0082	0.05	0.1868	1.2603	2.0617	1.2054	3.5265

Differences of Site Least Squares Means

Site	*_Site*	*Estimate*	*Standard Error*	*z Value*	*Pr > |z|*	*Alpha*	*Lower*	*Upper*	*Exponentiated*	*Exponentiated Lower*	*Exponentiated Upper*
1	2	0.1514	0.4862	0.31	0.7554	0.05	-0.8015	1.1044	1.1635	0.4486	3.0175
1	3	-0.6022	0.4734	-1.27	0.2033	0.05	-1.5299	0.3256	0.5476	0.2166	1.3849
2	3	-0.7536	0.3078	-2.45	0.0144	0.05	-1.3569	-0.1503	0.4707	0.2575	0.8604

team_1Obstetricianx Least Squares Means

Pain_sum	*team_1Obstetricianx*	*Estimate*	*Standard Error*	*z Value*	*Pr > |z|*	*Alpha*	*Lower*	*Upper*	*Exponentiated*	*Exponentiated Lower*	*Exponentiated Upper*
4	1.Yes	-2.3514	0.3370	-6.98	<.0001	0.05	-3.0120	-1.6908	0.09524	0.04919	0.1844
4	2.No	-3.3835	0.3743	-9.04	<.0001	0.05	-4.1171	-2.6498	0.03393	0.01629	0.07066
3	1.Yes	-1.0400	0.3010	-3.46	0.0006	0.05	-1.6299	-0.4500	0.3535	0.1959	0.6376
3	2.No	-2.0720	0.3331	-6.22	<.0001	0.05	-2.7248	-1.4192	0.1259	0.06556	0.2419
2	1.Yes	0.7877	0.2978	2.65	0.0082	0.05	0.2040	1.3713	2.1982	1.2263	3.9405
2	2.No	-0.2444	0.3045	-0.80	0.4222	0.05	-0.8413	0.3525	0.7832	0.4312	1.4226

Differences of team_1Obstetricianx Least Squares Means

team_1Obstetricianx	*_team_1Obstetricianx*	*Estimate*	*Standard Error*	*z Value*	*Pr > |z|*	*Alpha*	*Lower*	*Upper*	*Exponentiated*	*Exponentiated Lower*	*Exponentiated Upper*
1.Yes	2.No	1.0321	0.3595	2.87	0.0041	0.05	0.3274	1.7367	2.8068	1.3873	5.6786

Pain_sum	team_5Anaesthetistx	Estimate	Standard Error	z Value	Pr > |z|	Alpha	Lower	Upper	Exponentiated	Exponentiated Lower	Exponentiated Upper
4	1.Yes	-2.3487	0.2738	-8.58	<.0001	0.05	-2.8855	-1.8120	0.09549	0.05583	0.1633
4	2.No	-3.3861	0.4611	-7.34	<.0001	0.05	-4.2898	-2.4824	0.03384	0.01371	0.08354
3	1.Yes	-1.0373	0.2250	-4.61	<.0001	0.05	-1.4784	-0.5963	0.3544	0.2280	0.5509
3	2.No	-2.0747	0.4298	-4.83	<.0001	0.05	-2.9172	-1.2322	0.1256	0.05409	0.2917
2	1.Yes	0.7903	0.2247	3.52	0.0004	0.05	0.3499	1.2307	2.2041	1.4190	3.4236
2	2.No	-0.2471	0.4060	-0.61	0.5428	0.05	-1.0427	0.5486	0.7811	0.3525	1.7309

team_5Anaesthetistx Least Squares Means

Differences of team_5Anaesthetistx Least Squares Means

team_5Anaesthetistx	_team_5Anaesthetistx	Estimate	Standard Error	z Value	Pr > |z|	Alpha	Lower	Upper	Exponentiated	Exponentiated Lower	Exponentiated Upper
1.Yes	2.No	1.0374	0.4439	2.34	0.0194	0.05	0.1674	1.9073	2.8218	1.1822	6.7350

Model 11 Final Multivariable Logistic regression of Pain_1Anaesthetic versus Group, site and confounders

The GENMOD Procedure

Model Information

Data Set	WORK.CARDIAC2
Distribution	Binomial
Link Function	Logit
Dependent Variable	Pain_1Anaesthetic

Number of Observations Read	271
Number of Observations Used	193
Number of Events	91
Number of Trials	193
Missing Values	78

Class Level Information		
Class	Levels	Values
Revised_Groups	2	Acquired Pre-existant
Site	3	1 2 3
Highrisk1	2	1.Yes 2.No
team_5Anaesthetistx	2	1.Yes 2.No

Response Profile		
Ordered Value	Pain_1Anaesthetic	Total Frequency
1	1	91
2	0	102

PROC GENMOD is modeling the probability that Pain_1Anaesthetic='1'.

Parameter Information					
Parameter	Effect	Revised_Groups	Site	Highrisk1	team_5Anaesthetistx
Prm1	Intercept				
Prm2	Revised_Groups	Acquired			
Prm3	Revised_Groups	Pre-existant			
Prm4	Site		1		
Prm5	Site		2		
Prm6	Site		3		
Prm7	Highrisk1			1.Yes	
Prm8	Highrisk1			2.No	
Prm9	team_5Anaesthetistx				1.Yes
Prm10	team_5Anaesthetistx				2.No
Prm11	Live_baby_weight				

Criteria For Assessing Goodness Of Fit			
Criterion	*DF*	*Value*	*Value/DF*
Log Likelihood		-114.2735	
Full Log Likelihood		-114.2735	
AIC (smaller is better)		242.5470	
AICC (smaller is better)		243.1524	
BIC (smaller is better)		265.3859	

Algorithm converged.

Analysis Of Maximum Likelihood Parameter Estimates								
Parameter		*DF*	*Estimate*	*Standard Error*	*Wald 95% Confidence Limits*		*Wald Chi-Square*	*Pr > ChiSq*
Intercept		1	1.2085	0.9792	-0.7107	3.1277	1.52	0.2171
Revised_Groups	Acquired	1	-0.9199	0.3484	-1.6028	-0.2370	6.97	0.0083
Revised_Groups	Pre-existant	0	0.0000	0.0000	0.0000	0.0000	.	.
Site	1	1	-0.4199	0.5836	-1.5636	0.7239	0.52	0.4718
Site	2	1	-0.6504	0.3493	-1.3350	0.0342	3.47	0.0626
Site	3	0	0.0000	0.0000	0.0000	0.0000	.	.
Highrisk1	1.Yes	1	0.9424	0.3616	0.2337	1.6511	6.79	0.0092
Highrisk1	2.No	0	0.0000	0.0000	0.0000	0.0000	.	.
team_5Anaesthetistx	1.Yes	1	1.0283	0.4907	0.0664	1.9901	4.39	0.0361
team_5Anaesthetistx	2.No	0	0.0000	0.0000	0.0000	0.0000	.	.
Live_baby_weight		1	-0.0007	0.0002	-0.0012	-0.0002	8.20	0.0042
Scale		0	1.0000	0.0000	1.0000	1.0000		

Note: The scale parameter was held fixed.

Wald Statistics For Type 3 Analysis			
Source	DF	Chi-Square	Pr > ChiSq
Revised_Groups	1	6.97	0.0083
Site	2	3.49	0.1745
Highrisk1	1	6.79	0.0092
team_5Anaesthetistx	1	4.39	0.0361
Live_baby_weight	1	8.20	0.0042

Contrast Estimate Results

Label	Mean Estimate	Mean Confidence Limits		L'Beta Estimate	Standard Error	Alpha	L'Beta Confidence Limits		Chi-Square	Pr > ChiSq
Live_baby_weight per 1kg increase	0.3328	0.2365	0.4454	-0.6956	0.2429	0.05	-1.1718	-0.2195	8.20	0.0042
Exp(Live_baby_weight per 1kg increase)				0.4988	0.1212	0.05	0.3098	0.8030		

Revised_Groups Least Squares Means

Revised_Groups	Estimate	Standard Error	z Value	Pr > |z|	Alpha	Lower	Upper	Exponentiated	Exponentiated Lower	Exponentiated Upper
Acquired	-1.1995	0.3600	-3.33	0.0009	0.05	-1.9051	-0.4939	0.3013	0.1488	0.6103
Pre-existant	-0.2796	0.3062	-0.91	0.3612	0.05	-0.8798	0.3206	0.7561	0.4149	1.3779

Differences of Revised_Groups Least Squares Means

Revised_Groups	_Revised_Groups	Estimate	Standard Error	z Value	Pr > |z|	Alpha	Lower	Upper	Exponentiated	Exponentiated Lower	Exponentiated Upper
Acquired	Pre-existant	-0.9199	0.3484	-2.64	0.0083	0.05	-1.6028	-0.2370	0.3986	0.2013	0.7890

Site Least Squares Means

Site	Estimate	Standard Error	z Value	Pr > |z|	Alpha	Lower	Upper	Exponentiated	Exponentiated Lower	Exponentiated Upper
1	-0.8027	0.5595	-1.43	0.1514	0.05	-1.8992	0.2938	0.4481	0.1497	1.3416
2	-1.0332	0.3176	-3.25	0.0011	0.05	-1.6556	-0.4108	0.3559	0.1910	0.6631

Site Least Squares Means

Site	Estimate	Standard Error	z Value	Pr > |z|	Alpha	Lower	Upper	Exponentiated	Exponentiated Lower	Exponentiated Upper
3	-0.3828	0.3115	-1.23	0.2191	0.05	-0.9934	0.2278	0.6819	0.3703	1.2558

Differences of Site Least Squares Means

Site	_Site	Estimate	Standard Error	z Value	Pr > |z|	Alpha	Lower	Upper	Exponentiated	Exponentiated Lower	Exponentiated Upper
1	2	0.2305	0.5814	0.40	0.6917	0.05	-0.9090	1.3700	1.2592	0.4029	3.9354
1	3	-0.4199	0.5836	-0.72	0.4718	0.05	-1.5636	0.7239	0.6571	0.2094	2.0624
2	3	-0.6504	0.3493	-1.86	0.0626	0.05	-1.3350	0.03424	0.5218	0.2632	1.0348

Highrisk1 Least Squares Means

Highrisk1	Estimate	Standard Error	z Value	Pr > |z|	Alpha	Lower	Upper	Exponentiated	Exponentiated Lower	Exponentiated Upper
1.Yes	-0.2684	0.2977	-0.90	0.3674	0.05	-0.8519	0.3152	0.7646	0.4266	1.3705
2.No	-1.2108	0.3734	-3.24	0.0012	0.05	-1.9426	-0.4789	0.2980	0.1433	0.6195

Differences of Highrisk1 Least Squares Means

Highrisk1	_Highrisk1	Estimate	Standard Error	z Value	Pr > |z|	Alpha	Lower	Upper	Exponentiated	Exponentiated Lower	Exponentiated Upper
1.Yes	2.No	0.9424	0.3616	2.61	0.0092	0.05	0.2337	1.6511	2.5661	1.2633	5.2127

team_5Anaesthetistx Least Squares Means

team_5Anaesthetistx	Estimate	Standard Error	z Value	Pr > |z|	Alpha	Lower	Upper	Exponentiated	Exponentiated Lower	Exponentiated Upper
1.Yes	-0.2254	0.2368	-0.95	0.3411	0.05	-0.6896	0.2387	0.7982	0.5018	1.2696
2.No	-1.2537	0.4765	-2.63	0.0085	0.05	-2.1876	-0.3198	0.2854	0.1122	0.7263

Differences of team_5Anaesthetistx Least Squares Means												
team_5Anaesthetistx	_team_5Anaesthetistx	Estimate	Standard Error	z Value	Pr > |z|	Alpha	Lower	Upper	Exponentiated	Exponentiated Lower	Exponentiated Upper
1.Yes	2.No	1.0283	0.4907	2.10	0.0361	0.05	0.06645	1.9901	2.7962	1.0687	7.3161

Model 12 Final Multivariable Logistic regression of Pain_2Epidural versus Group, site and confounders

The GENMOD Procedure

Model Information	
Data Set	WORK.CARDIAC2
Distribution	Binomial
Link Function	Logit
Dependent Variable	Pain_2Epidural

Number of Observations Read	271
Number of Observations Used	203
Number of Events	157
Number of Trials	203
Missing Values	68

Class Level Information		
Class	Levels	Values
Revised_Groups	2	Acquired Pre-existant
Site	3	1 2 3
team_5Anaesthetistx	2	1.Yes 2.No

Response Profile		
Ordered Value	Pain_2Epidural	Total Frequency
1	1	157
2	0	46

PROC GENMOD is modeling the probability that Pain_2Epidural='1'.

Parameter Information				
Parameter	Effect	Revised_Groups	Site	team_5Anaesthetistx
Prm1	Intercept			
Prm2	Revised_Groups	Acquired		
Prm3	Revised_Groups	Pre-existant		
Prm4	Site		1	
Prm5	Site		2	
Prm6	Site		3	
Prm7	team_5Anaesthetistx			1.Yes
Prm8	team_5Anaesthetistx			2.No
Prm9	Apgar_1min			

Criteria For Assessing Goodness Of Fit			
Criterion	DF	Value	Value/DF
Log Likelihood		-91.6808	
Full Log Likelihood		-91.6808	
AIC (smaller is better)		195.3615	
AICC (smaller is better)		195.7901	
BIC (smaller is better)		215.2408	

Algorithm converged.

Analysis Of Maximum Likelihood Parameter Estimates								
Parameter		DF	Estimate	Standard Error	Wald 95% Confidence Limits		Wald Chi-Square	Pr > ChiSq
Intercept		1	-2.2675	0.9241	-4.0788	-0.4563	6.02	0.0141
Revised_Groups	Acquired	1	-0.7592	0.3896	-1.5228	0.0045	3.80	0.0514
Revised_Groups	Pre-existant	0	0.0000	0.0000	0.0000	0.0000	.	.
Site	1	1	0.1179	0.6831	-1.2209	1.4567	0.03	0.8630
Site	2	1	-0.3128	0.4152	-1.1266	0.5011	0.57	0.4513
Site	3	0	0.0000	0.0000	0.0000	0.0000	.	.
team_5Anaesthetistx	1.Yes	1	1.9225	0.4410	1.0582	2.7869	19.00	<.0001
team_5Anaesthetistx	2.No	0	0.0000	0.0000	0.0000	0.0000	.	.
Apgar_1min		1	0.3244	0.0875	0.1530	0.4959	13.75	0.0002
Scale		0	1.0000	0.0000	1.0000	1.0000		

Note: The scale parameter was held fixed.

Wald Statistics For Type 3 Analysis			
Source	DF	Chi-Square	Pr > ChiSq
Revised_Groups	1	3.80	0.0514
Site	2	0.74	0.6897
team_5Anaesthetistx	1	19.00	<.0001
Apgar_1min	1	13.75	0.0002

Contrast Estimate Results										
Label	Mean Estimate	Mean Confidence Limits		L'Beta Estimate	Standard Error	Alpha	L'Beta Confidence Limits		Chi-Square	Pr > ChiSq
Apgar_1min	0.5804	0.5382	0.6215	0.3244	0.0875	0.05	0.1530	0.4959	13.75	0.0002
Exp(Apgar_1min)				1.3832	0.1210	0.05	1.1653	1.6419		

Revised_Groups Least Squares Means

Revised_Groups	Estimate	Standard Error	z Value	Pr > |z|	Alpha	Lower	Upper	Exponentiated	Exponentiated Lower	Exponentiated Upper
Acquired	0.4393	0.3140	1.40	0.1617	0.05	-0.1760	1.0547	1.5517	0.8386	2.8711
Pre-existant	1.1985	0.3349	3.58	0.0003	0.05	0.5422	1.8548	3.3151	1.7198	6.3905

Differences of Revised_Groups Least Squares Means

Revised_Groups	_Revised_Groups	Estimate	Standard Error	z Value	Pr > |z|	Alpha	Lower	Upper	Exponentiated	Exponentiated Lower	Exponentiated Upper
Acquired	Pre-existant	-0.7592	0.3896	-1.95	0.0514	0.05	-1.5228	0.004458	0.4681	0.2181	1.0045

Site Least Squares Means

Site	Estimate	Standard Error	z Value	Pr > |z|	Alpha	Lower	Upper	Exponentiated	Exponentiated Lower	Exponentiated Upper
1	1.0018	0.6221	1.61	0.1073	0.05	-0.2175	2.2210	2.7230	0.8045	9.2170
2	0.5711	0.2964	1.93	0.0540	0.05	-0.00981	1.1520	1.7702	0.9902	3.1646
3	0.8839	0.3105	2.85	0.0044	0.05	0.2754	1.4924	2.4203	1.3171	4.4476

Differences of Site Least Squares Means

Site	_Site	Estimate	Standard Error	z Value	Pr > |z|	Alpha	Lower	Upper	Exponentiated	Exponentiated Lower	Exponentiated Upper
1	2	0.4306	0.6842	0.63	0.5291	0.05	-0.9103	1.7716	1.5382	0.4024	5.8801
1	3	0.1179	0.6831	0.17	0.8630	0.05	-1.2209	1.4567	1.1251	0.2950	4.2917
2	3	-0.3128	0.4152	-0.75	0.4513	0.05	-1.1266	0.5011	0.7314	0.3241	1.6505

team_5Anaesthetistx Least Squares Means

team_5Anaesthetistx	Estimate	Standard Error	z Value	Pr > |z|	Alpha	Lower	Upper	Exponentiated	Exponentiated Lower	Exponentiated Upper
1.Yes	1.7802	0.2821	6.31	<.0001	0.05	1.2273	2.3331	5.9310	3.4119	10.3098
2.No	-0.1424	0.3905	-0.36	0.7154	0.05	-0.9077	0.6230	0.8673	0.4035	1.8645

Differences of team_5Anaesthetistx Least Squares Means											
team_5Anaesthetistx	_team_5Anaesthetistx	Estimate	Standard Error	z Value	Pr > |z|	Alpha	Lower	Upper	Exponentiated	Exponentiated Lower	Exponentiated Upper
1.Yes	2.No	1.9225	0.4410	4.36	<.0001	0.05	1.0582	2.7869	6.8383	2.8811	16.2310

Model 13 Final Multivariable Logistic regression of Pain_3Combined versus Group, site and confounders

The GENMOD Procedure

Model Information	
Data Set	WORK.CARDIAC2
Distribution	Binomial
Link Function	Logit
Dependent Variable	Pain_3Combined

Number of Observations Read	271
Number of Observations Used	192
Number of Events	70
Number of Trials	192
Missing Values	79

Class Level Information		
Class	*Levels*	*Values*
Revised_Groups	2	Acquired Pre-existant
Site	3	1 2 3
Highrisk3	2	1.Yes 2.No
Highrisk9_4Nox	2	1.Yes 2.No
team_1Obstetricianx	2	1.Yes 2.No
team_4Physicianx	2	1.Yes 2.No
team_5Anaesthetistx	2	1.Yes 2.No

Response Profile		
Ordered Value	Pain_3Combined	Total Frequency
1	1	70
2	0	122

PROC GENMOD is modeling the probability that Pain_3Combined='1'.

Parameter Information								
Parameter	Effect	Revised_Groups	Site	Highrisk3	Highrisk9_4Nox	team_1Obstetricianx	team_4Physicianx	team_5Anaesthetistx
Prm1	Intercept							
Prm2	Revised_Groups	Acquired						
Prm3	Revised_Groups	Pre-existant						
Prm4	Site		1					
Prm5	Site		2					
Prm6	Site		3					
Prm7	Highrisk3			1.Yes				
Prm8	Highrisk3			2.No				
Prm9	Highrisk9_4Nox				1.Yes			
Prm10	Highrisk9_4Nox				2.No			
Prm11	team_1Obstetricianx					1.Yes		
Prm12	team_1Obstetricianx					2.No		
Prm13	team_4Physicianx						1.Yes	
Prm14	team_4Physicianx						2.No	
Prm15	team_5Anaesthetistx							1.Yes
Prm16	team_5Anaesthetistx							2.No

Criteria For Assessing Goodness Of Fit			
Criterion	DF	Value	Value/DF
Log Likelihood		-97.4691	

Criteria For Assessing Goodness Of Fit			
Criterion	DF	Value	Value/DF
Full Log Likelihood		-97.4691	
AIC (smaller is better)		212.9381	
AICC (smaller is better)		213.9271	
BIC (smaller is better)		242.2556	

Algorithm converged.

Analysis Of Maximum Likelihood Parameter Estimates								
Parameter		DF	Estimate	Standard Error	Wald 95% Confidence Limits		Wald Chi-Square	Pr > ChiSq
Intercept		1	-7.7783	1.6731	-11.0575	-4.4991	21.61	<.0001
Revised_Groups	Acquired	1	0.3455	0.3760	-0.3914	1.0825	0.84	0.3581
Revised_Groups	Pre-existant	0	0.0000	0.0000	0.0000	0.0000	.	.
Site	1	1	-1.3970	0.8613	-3.0852	0.2912	2.63	0.1048
Site	2	1	0.0834	0.4199	-0.7396	0.9064	0.04	0.8425
Site	3	0	0.0000	0.0000	0.0000	0.0000	.	.
Highrisk3	1.Yes	1	2.2568	1.0912	0.1180	4.3955	4.28	0.0386
Highrisk3	2.No	0	0.0000	0.0000	0.0000	0.0000	.	.
Highrisk9_4Nox	1.Yes	1	1.3559	0.6099	0.1606	2.5513	4.94	0.0262
Highrisk9_4Nox	2.No	0	0.0000	0.0000	0.0000	0.0000	.	.
team_1Obstetricianx	1.Yes	1	1.2921	0.6265	0.0643	2.5200	4.25	0.0392
team_1Obstetricianx	2.No	0	0.0000	0.0000	0.0000	0.0000	.	.
team_4Physicianx	1.Yes	1	0.7859	0.3876	0.0263	1.5456	4.11	0.0426
team_4Physicianx	2.No	0	0.0000	0.0000	0.0000	0.0000	.	.
team_5Anaesthetistx	1.Yes	1	2.4502	1.0689	0.3552	4.5452	5.25	0.0219
team_5Anaesthetistx	2.No	0	0.0000	0.0000	0.0000	0.0000	.	.
Scale		0	1.0000	0.0000	1.0000	1.0000		

Note: The scale parameter was held fixed.

Wald Statistics For Type 3 Analysis

Source	*DF*	*Chi-Square*	*Pr > ChiSq*
Revised_Groups	1	0.84	0.3581
Site	2	3.18	0.2040
Highrisk3	1	4.28	0.0386
Highrisk9_4Nox	1	4.94	0.0262
team_1Obstetricianx	1	4.25	0.0392
team_4Physicianx	1	4.11	0.0426
team_5Anaesthetistx	1	5.25	0.0219

Revised_Groups Least Squares Means

Revised_Groups	*Estimate*	*Standard Error*	*z Value*	*Pr > |z|*	*Alpha*	*Lower*	*Upper*	*Exponentiated*	*Exponentiated Lower*	*Exponentiated Upper*
Acquired	-3.8002	0.8736	-4.35	<.0001	0.05	-5.5124	-2.0880	0.02237	0.004037	0.1239
Pre-existant	-4.1457	0.8727	-4.75	<.0001	0.05	-5.8562	-2.4352	0.01583	0.002862	0.08758

Differences of Revised_Groups Least Squares Means

Revised_Groups	*_Revised_Groups*	*Estimate*	*Standard Error*	*z Value*	*Pr > |z|*	*Alpha*	*Lower*	*Upper*	*Exponentiated*	*Exponentiated Lower*	*Exponentiated Upper*
Acquired	Pre-existant	0.3455	0.3760	0.92	0.3581	0.05	-0.3914	1.0825	1.4128	0.6761	2.9520

Site Least Squares Means

Site	*Estimate*	*Standard Error*	*z Value*	*Pr > |z|*	*Alpha*	*Lower*	*Upper*	*Exponentiated*	*Exponentiated Lower*	*Exponentiated Upper*
1	-4.9321	1.1553	-4.27	<.0001	0.05	-7.1965	-2.6678	0.007211	0.000749	0.06941
2	-3.4516	0.8558	-4.03	<.0001	0.05	-5.1290	-1.7743	0.03169	0.005923	0.1696
3	-3.5351	0.8069	-4.38	<.0001	0.05	-5.1165	-1.9536	0.02916	0.005997	0.1418

Differences of Site Least Squares Means

Site	*_Site*	*Estimate*	*Standard Error*	*z Value*	*Pr > |z|*	*Alpha*	*Lower*	*Upper*	*Exponentiated*	*Exponentiated Lower*	*Exponentiated Upper*
1	2	-1.4805	0.8324	-1.78	0.0753	0.05	-3.1119	0.1510	0.2275	0.04451	1.1630
1	3	-1.3970	0.8613	-1.62	0.1048	0.05	-3.0852	0.2912	0.2473	0.04572	1.3380
2	3	0.08342	0.4199	0.20	0.8425	0.05	-0.7396	0.9064	1.0870	0.4773	2.4755

Highrisk3 Least Squares Means

Highrisk3	*Estimate*	*Standard Error*	*z Value*	*Pr > |z|*	*Alpha*	*Lower*	*Upper*	*Exponentiated*	*Exponentiated Lower*	*Exponentiated Upper*
1.Yes	-2.8445	0.6687	-4.25	<.0001	0.05	-4.1551	-1.5340	0.05816	0.01568	0.2157
2.No	-5.1013	1.2658	-4.03	<.0001	0.05	-7.5823	-2.6203	0.006089	0.000509	0.07278

Differences of Highrisk3 Least Squares Means

Highrisk3	*_Highrisk3*	*Estimate*	*Standard Error*	*z Value*	*Pr > |z|*	*Alpha*	*Lower*	*Upper*	*Exponentiated*	*Exponentiated Lower*	*Exponentiated Upper*
1.Yes	2.No	2.2568	1.0912	2.07	0.0386	0.05	0.1180	4.3955	9.5524	1.1253	81.0881

Highrisk9_4Nox Least Squares Means

Highrisk9_4Nox	*Estimate*	*Standard Error*	*z Value*	*Pr > |z|*	*Alpha*	*Lower*	*Upper*	*Exponentiated*	*Exponentiated Lower*	*Exponentiated Upper*
1.Yes	-3.2950	0.8051	-4.09	<.0001	0.05	-4.8728	-1.7171	0.03707	0.007652	0.1796
2.No	-4.6509	0.9960	-4.67	<.0001	0.05	-6.6030	-2.6988	0.009553	0.001356	0.06728

Differences of Highrisk9_4Nox Least Squares Means

Highrisk9_4Nox	*_Highrisk9_4Nox*	*Estimate*	*Standard Error*	*z Value*	*Pr > |z|*	*Alpha*	*Lower*	*Upper*	*Exponentiated*	*Exponentiated Lower*	*Exponentiated Upper*
1.Yes	2.No	1.3559	0.6099	2.22	0.0262	0.05	0.1606	2.5513	3.8804	1.1742	12.8236

team_1Obstetricianx Least Squares Means

team_1Obstetricianx	Estimate	Standard Error	z Value	Pr > |z|	Alpha	Lower	Upper	Exponentiated	Exponentiated Lower	Exponentiated Upper
1.Yes	-3.3269	0.8436	-3.94	<.0001	0.05	-4.9804	-1.6734	0.03590	0.006871	0.1876
2.No	-4.6190	0.9688	-4.77	<.0001	0.05	-6.5178	-2.7202	0.009863	0.001477	0.06586

Differences of team_1Obstetricianx Least Squares Means

team_1Obstetricianx	_team_1Obstetricianx	Estimate	Standard Error	z Value	Pr > |z|	Alpha	Lower	Upper	Exponentiated	Exponentiated Lower	Exponentiated Upper
1.Yes	2.No	1.2921	0.6265	2.06	0.0392	0.05	0.06427	2.5200	3.6405	1.0664	12.4283

team_4Physicianx Least Squares Means

team_4Physicianx	Estimate	Standard Error	z Value	Pr > |z|	Alpha	Lower	Upper	Exponentiated	Exponentiated Lower	Exponentiated Upper
1.Yes	-3.5800	0.8777	-4.08	<.0001	0.05	-5.3003	-1.8597	0.02788	0.004990	0.1557
2.No	-4.3659	0.8711	-5.01	<.0001	0.05	-6.0732	-2.6586	0.01270	0.002304	0.07005

Differences of team_4Physicianx Least Squares Means

team_4Physicianx	_team_4Physicianx	Estimate	Standard Error	z Value	Pr > |z|	Alpha	Lower	Upper	Exponentiated	Exponentiated Lower	Exponentiated Upper
1.Yes	2.No	0.7859	0.3876	2.03	0.0426	0.05	0.02628	1.5456	2.1944	1.0266	4.6906

team_5Anaesthetistx Least Squares Means

team_5Anaesthetistx	Estimate	Standard Error	z Value	Pr > |z|	Alpha	Lower	Upper	Exponentiated	Exponentiated Lower	Exponentiated Upper
1.Yes	-2.7479	0.7033	-3.91	<.0001	0.05	-4.1263	-1.3695	0.06406	0.01614	0.2542
2.No	-5.1980	1.2372	-4.20	<.0001	0.05	-7.6230	-2.7731	0.005527	0.000489	0.06247

Differences of team_5Anaesthetistx Least Squares Means											
team_5Anaesthetistx	_team_5Anaesthetistx	Estimate	Standard Error	z Value	Pr > |z|	Alpha	Lower	Upper	Exponentiated	Exponentiated Lower	Exponentiated Upper
1.Yes	2.No	2.4502	1.0689	2.29	0.0219	0.05	0.3552	4.5452	11.5902	1.4264	94.1748

Model 14 Final Multivariable Logistic regression of Pain_4Other versus Group, site and confounders

The GENMOD Procedure

Model Information	
Data Set	WORK.CARDIAC2
Distribution	Binomial
Link Function	Logit
Dependent Variable	Pain_4Other

Number of Observations Read	271
Number of Observations Used	194
Number of Events	113
Number of Trials	194
Missing Values	77

Class Level Information		
Class	Levels	Values
Revised_Groups	2	Acquired Pre-existant
Site	3	1 2 3
Highrisk1	2	1.Yes 2.No

Response Profile		
Ordered Value	Pain_4Other	Total Frequency
1	1	113
2	0	81

PROC GENMOD is modeling the probability that Pain_4Other='1'.

Parameter Information				
Parameter	Effect	Revised_Groups	Site	Highrisk1
Prm1	Intercept			
Prm2	Revised_Groups	Acquired		
Prm3	Revised_Groups	Pre-existant		
Prm4	Site		1	
Prm5	Site		2	
Prm6	Site		3	
Prm7	Highrisk1			1.Yes
Prm8	Highrisk1			2.No
Prm9	Live_baby_weight			

Criteria For Assessing Goodness Of Fit			
Criterion	DF	Value	Value/DF
Log Likelihood		-122.4862	
Full Log Likelihood		-122.4862	
AIC (smaller is better)		256.9725	
AICC (smaller is better)		257.4216	
BIC (smaller is better)		276.5796	

Algorithm converged.

Analysis Of Maximum Likelihood Parameter Estimates								
Parameter		*DF*	*Estimate*	*Standard Error*	*Wald 95% Confidence Limits*		*Wald Chi-Square*	*Pr > ChiSq*
Intercept		1	1.9477	0.8536	0.2747	3.6208	5.21	0.0225
Revised_Groups	Acquired	1	-0.6735	0.3234	-1.3073	-0.0397	4.34	0.0373
Revised_Groups	Pre-existant	0	0.0000	0.0000	0.0000	0.0000	.	.
Site	1	1	-0.3252	0.5539	-1.4108	0.7604	0.34	0.5571
Site	2	1	-0.2343	0.3310	-0.8830	0.4144	0.50	0.4791
Site	3	0	0.0000	0.0000	0.0000	0.0000	.	.
Highrisk1	1.Yes	1	0.7068	0.3357	0.0489	1.3647	4.43	0.0352
Highrisk1	2.No	0	0.0000	0.0000	0.0000	0.0000	.	.
Live_baby_weight		1	-0.0005	0.0002	-0.0010	-0.0001	5.25	0.0219
Scale		0	1.0000	0.0000	1.0000	1.0000		

Note: The scale parameter was held fixed.

Wald Statistics For Type 3 Analysis			
Source	*DF*	*Chi-Square*	*Pr > ChiSq*
Revised_Groups	1	4.34	0.0373
Site	2	0.65	0.7209
Highrisk1	1	4.43	0.0352
Live_baby_weight	1	5.25	0.0219

Contrast Estimate Results										
Label	*Mean Estimate*	*Mean Confidence Limits*		*L'Beta Estimate*	*Standard Error*	*Alpha*	*L'Beta Confidence Limits*		*Chi-Square*	*Pr > ChiSq*
Live_baby_weight per 1kg increase	0.3700	0.2714	0.4808	-0.5321	0.2322	0.05	-0.9873	-0.0770	5.25	0.0219
Exp(Live_baby_weight per 1kg increase)				0.5874	0.1364	0.05	0.3726	0.9259		

Revised_Groups Least Squares Means

Revised_Groups	*Estimate*	*Standard Error*	*z Value*	*Pr > |z|*	*Alpha*	*Lower*	*Upper*	*Exponentiated*	*Exponentiated Lower*	*Exponentiated Upper*
Acquired	-0.1783	0.2778	-0.64	0.5210	0.05	-0.7229	0.3662	0.8367	0.4853	1.4423
Pre-existant	0.4952	0.2480	2.00	0.0459	0.05	0.009030	0.9813	1.6408	1.0091	2.6680

Differences of Revised_Groups Least Squares Means

Revised_Groups	*_Revised_Groups*	*Estimate*	*Standard Error*	*z Value*	*Pr > |z|*	*Alpha*	*Lower*	*Upper*	*Exponentiated*	*Exponentiated Lower*	*Exponentiated Upper*
Acquired	Pre-existant	-0.6735	0.3234	-2.08	0.0373	0.05	-1.3073	-0.03969	0.5099	0.2705	0.9611

Site Least Squares Means

Site	*Estimate*	*Standard Error*	*z Value*	*Pr > |z|*	*Alpha*	*Lower*	*Upper*	*Exponentiated*	*Exponentiated Lower*	*Exponentiated Upper*
1	0.01968	0.5078	0.04	0.9691	0.05	-0.9757	1.0150	1.0199	0.3769	2.7595
2	0.1107	0.2329	0.48	0.6347	0.05	-0.3458	0.5672	1.1170	0.7076	1.7632
3	0.3449	0.2412	1.43	0.1527	0.05	-0.1278	0.8176	1.4119	0.8801	2.2651

Differences of Site Least Squares Means

Site	*_Site*	*Estimate*	*Standard Error*	*z Value*	*Pr > |z|*	*Alpha*	*Lower*	*Upper*	*Exponentiated*	*Exponentiated Lower*	*Exponentiated Upper*
1	2	-0.09098	0.5529	-0.16	0.8693	0.05	-1.1746	0.9926	0.9130	0.3090	2.6983
1	3	-0.3252	0.5539	-0.59	0.5571	0.05	-1.4108	0.7604	0.7224	0.2439	2.1391
2	3	-0.2343	0.3310	-0.71	0.4791	0.05	-0.8830	0.4144	0.7912	0.4136	1.5135

Highrisk1 Least Squares Means

Highrisk1	*Estimate*	*Standard Error*	*z Value*	*Pr > |z|*	*Alpha*	*Lower*	*Upper*	*Exponentiated*	*Exponentiated Lower*	*Exponentiated Upper*
1.Yes	0.5118	0.2256	2.27	0.0233	0.05	0.06964	0.9540	1.6683	1.0721	2.5961
2.No	-0.1950	0.3031	-0.64	0.5200	0.05	-0.7891	0.3991	0.8228	0.4543	1.4905

Differences of Highrisk1 Least Squares Means												
Highrisk1	_Highrisk1	Estimate	Standard Error	z Value	Pr > |z|	Alpha	Lower	Upper	Exponentiated	Exponentiated Lower	Exponentiated Upper
1.Yes	2.No	0.7068	0.3357	2.11	0.0352	0.05	0.04891	1.3647	2.0275	1.0501	3.9146

Model 16 Final Multivariable Logistic regression of Paediatric_neonatal_staff versus Group, site and confounders

The GENMOD Procedure

Model Information	
Data Set	WORK.CARDIAC2
Distribution	Binomial
Link Function	Logit
Dependent Variable	Paediatric_neonatal_staff

Number of Observations Read	271
Number of Observations Used	195
Number of Events	154
Number of Trials	195
Missing Values	76

Class Level Information		
Class	Levels	Values
Revised_Groups	2	Acquired Pre-existant
Site	3	1 2 3
Highrisk5_3Paediatri	2	1.Yes 2.No
team_4Physicianx	2	1.Yes 2.No

Response Profile		
Ordered Value	Paediatric_neonatal_staff	Total Frequency
1	Yes	154
2	No	41

PROC GENMOD is modeling the probability that Paediatric_neonatal_staff='Yes'.

Parameter Information					
Parameter	Effect	Revised_Groups	Site	Highrisk5_3Paediatri	team_4Physicianx
Prm1	Intercept				
Prm2	Revised_Groups	Acquired			
Prm3	Revised_Groups	Pre-existant			
Prm4	Site		1		
Prm5	Site		2		
Prm6	Site		3		
Prm7	Highrisk5_3Paediatri			1.Yes	
Prm8	Highrisk5_3Paediatri			2.No	
Prm9	team_4Physicianx				1.Yes
Prm10	team_4Physicianx				2.No
Prm11	Live_baby_weight				

Criteria For Assessing Goodness Of Fit			
Criterion	DF	Value	Value/DF
Log Likelihood		-58.6168	
Full Log Likelihood		-58.6168	
AIC (smaller is better)		131.2336	
AICC (smaller is better)		131.8325	
BIC (smaller is better)		154.1446	

Algorithm converged.

Analysis Of Maximum Likelihood Parameter Estimates								
Parameter		DF	Estimate	Standard Error	Wald 95% Confidence Limits		Wald Chi-Square	Pr > ChiSq
Intercept		1	1.2456	1.4245	-1.5463	4.0375	0.76	0.3819
Revised_Groups	Acquired	1	-0.4550	0.5032	-1.4412	0.5311	0.82	0.3658
Revised_Groups	Pre-existant	0	0.0000	0.0000	0.0000	0.0000	.	.
Site	1	1	0.2018	0.8950	-1.5524	1.9559	0.05	0.8216
Site	2	1	0.4128	0.5886	-0.7409	1.5664	0.49	0.4831
Site	3	0	0.0000	0.0000	0.0000	0.0000	.	.
Highrisk5_3Paediatri	1.Yes	1	2.7886	0.5275	1.7547	3.8224	27.94	<.0001
Highrisk5_3Paediatri	2.No	0	0.0000	0.0000	0.0000	0.0000	.	.
team_4Physicianx	1.Yes	1	1.9718	0.5801	0.8349	3.1087	11.56	0.0007
team_4Physicianx	2.No	0	0.0000	0.0000	0.0000	0.0000	.	.
Live_baby_weight		1	-0.0008	0.0004	-0.0016	-0.0000	3.92	0.0476
Scale		0	1.0000	0.0000	1.0000	1.0000		

Note: The scale parameter was held fixed.

Wald Statistics For Type 3 Analysis			
Source	DF	Chi-Square	Pr > ChiSq
Revised_Groups	1	0.82	0.3658
Site	2	0.50	0.7804
Highrisk5_3Paediatri	1	27.94	<.0001
team_4Physicianx	1	11.56	0.0007
Live_baby_weight	1	3.92	0.0476

Contrast Estimate Results

Label	*Mean Estimate*	*Mean Confidence Limits*		*L'Beta Estimate*	*Standard Error*	*Alpha*	*L'Beta Confidence Limits*		*Chi-Square*	*Pr > ChiSq*
Live_baby_weight per 1kg increase	0.3073	0.1656	0.4979	-0.8127	0.4103	0.05	-1.6169	-0.0084	3.92	0.0476
Exp(Live_baby_weight per 1kg increase)				0.4437	0.1821	0.05	0.1985	0.9916		

Revised_Groups Least Squares Means

Revised_Groups	*Estimate*	*Standard Error*	*z Value*	*Pr > |z|*	*Alpha*	*Lower*	*Upper*	*Exponentiated*	*Exponentiated Lower*	*Exponentiated Upper*
Acquired	0.9207	0.4281	2.15	0.0315	0.05	0.08166	1.7597	2.5110	1.0851	5.8109
Pre-existant	1.3757	0.4197	3.28	0.0010	0.05	0.5532	2.1983	3.9580	1.7389	9.0092

Differences of Revised_Groups Least Squares Means

Revised_Groups	*_Revised_Groups*	*Estimate*	*Standard Error*	*z Value*	*Pr > |z|*	*Alpha*	*Lower*	*Upper*	*Exponentiated*	*Exponentiated Lower*	*Exponentiated Upper*
Acquired	Pre-existant	-0.4550	0.5032	-0.90	0.3658	0.05	-1.4412	0.5311	0.6344	0.2366	1.7009

Site Least Squares Means

Site	*Estimate*	*Standard Error*	*z Value*	*Pr > |z|*	*Alpha*	*Lower*	*Upper*	*Exponentiated*	*Exponentiated Lower*	*Exponentiated Upper*
1	1.1451	0.8205	1.40	0.1628	0.05	-0.4631	2.7533	3.1429	0.6294	15.6948
2	1.3562	0.4667	2.91	0.0037	0.05	0.4415	2.2709	3.8813	1.5550	9.6878
3	0.9434	0.3762	2.51	0.0122	0.05	0.2059	1.6808	2.5686	1.2287	5.3699

Differences of Site Least Squares Means

Site	*_Site*	*Estimate*	*Standard Error*	*z Value*	*Pr > |z|*	*Alpha*	*Lower*	*Upper*	*Exponentiated*	*Exponentiated Lower*	*Exponentiated Upper*
1	2	-0.2110	0.9502	-0.22	0.8242	0.05	-2.0734	1.6513	0.8098	0.1258	5.2140
1	3	0.2018	0.8950	0.23	0.8216	0.05	-1.5524	1.9559	1.2236	0.2117	7.0703
2	3	0.4128	0.5886	0.70	0.4831	0.05	-0.7409	1.5664	1.5110	0.4767	4.7895

Highrisk5_3Paediatri Least Squares Means

Highrisk5_3Paediatricianx	*Estimate*	*Standard Error*	*z Value*	*Pr > |z|*	*Alpha*	*Lower*	*Upper*	*Exponentiated*	*Exponentiated Lower*	*Exponentiated Upper*
1.Yes	2.5425	0.4283	5.94	<.0001	0.05	1.7030	3.3820	12.7114	5.4905	29.4289
2.No	-0.2461	0.4341	-0.57	0.5709	0.05	-1.0969	0.6048	0.7819	0.3339	1.8309

Differences of Highrisk5_3Paediatri Least Squares Means

Highrisk5_3Paediatricianx	*_Highrisk5_3Paediatricianx*	*Estimate*	*Standard Error*	*z Value*	*Pr > |z|*	*Alpha*	*Lower*	*Upper*	*Exponentiated*	*Exponentiated Lower*	*Exponentiated Upper*
1.Yes	2.No	2.7886	0.5275	5.29	<.0001	0.05	1.7547	3.8224	16.2575	5.7815	45.7160

team_4Physicianx Least Squares Means

team_4Physicianx	*Estimate*	*Standard Error*	*z Value*	*Pr > |z|*	*Alpha*	*Lower*	*Upper*	*Exponentiated*	*Exponentiated Lower*	*Exponentiated Upper*
1.Yes	2.1341	0.4932	4.33	<.0001	0.05	1.1675	3.1007	8.4496	3.2140	22.2144
2.No	0.1623	0.3972	0.41	0.6828	0.05	-0.6162	0.9409	1.1762	0.5400	2.5622

Differences of team_4Physicianx Least Squares Means

team_4Physicianx	*_team_4Physicianx*	*Estimate*	*Standard Error*	*z Value*	*Pr > |z|*	*Alpha*	*Lower*	*Upper*	*Exponentiated*	*Exponentiated Lower*	*Exponentiated Upper*
1.Yes	2.No	1.9718	0.5801	3.40	0.0007	0.05	0.8349	3.1087	7.1836	2.3046	22.3919
