# Supplementary material for: Adherence to clinical practice guidelines for South Australian pregnant women with cardiac conditions between 2003 and 2013
Source: PLoS One. 2020 Mar 17;15(3):e0230459. doi: 10.1371/journal.pone.0230459 (PMC7077829; doi:10.1371/journal.pone.0230459)
Supplement: S3 Table — (PDF) [file pone.0230459.s003.pdf]

S3 Linear regressions of total score versus various predictors

Model number	*Predictor*	*Comparison*	*Estimate (95% CI)*	*Comparison P value*	*Global P value*
1	Revised_Groups	Acquired vs Pre-existent	-2.31 (-3.88, -0.74)		0.0039
2	Plannedcare3x	Caesarean vs Vaginal	6.04 (4.58, 7.49)		<.0001
3	RaceC	ATSI vs Asian	1.84 (-2.19, 5.87)	0.3706	0.1298
		ATSI vs Caucasian	2.65 (0.25, 5.05)	0.0305	
		ATSI vs Other	5.11 (-1.12, 11.33)	0.1077	
		Asian vs Caucasian	0.81 (-2.66, 4.28)	0.6473	
		Asian vs Other	3.27 (-3.44, 9.97)	0.3398	
		Caucasian vs Other	2.46 (-3.42, 8.33)	0.4126	
4	Site	1 vs 2	-3.42 (-5.95, -0.88)	0.0082	<0.0001
		1 vs 3	0.53 (-2.00, 3.06)	0.6832	
		2 vs 3	3.94 (2.32, 5.56)	<.0001	
5	PC	Metropolitan vs Remote	-5.05 (-8.87, -1.24)	0.0095	0.0009
		Metropolitan vs Rural	-3.13 (-5.21, -1.05)	0.0031	
		Remote vs Rural	1.92 (-2.24, 6.08)	0.3649	
6	Status	De facto/ living with partner versus Divorced/Separated	0.22 (-5.33, 5.78)	0.9381	0.1814
		De facto/ living with partner versus Married	1.79 (-0.21, 3.79)	0.0789	
		De facto/ living with partner versus Never married	2.87 (0.03, 5.71)	0.0473	
		Divorced/Separated vs Married	1.57 (-3.80, 6.95)	0.5663	
		Divorced/Separated vs Never Married	2.65 (-3.09, 8.39)	0.3652	
		Married vs Never married	1.08 (-1.39, 3.54)	0.3912	
7	Highrisk9_1episiotom	No vs Yes	-0.96 (-5.22, 3.29)		0.6571
8	Highrisk9_2forcepsx	Yes vs No	2.39 (-0.28, 5.06)		0.0798
9	Highrisk9_4No	No vs Yes	0.97 (-1.46, 3.40)		0.4354
10	Prev_preg_cardiac	No vs Yes	-1.66 (-3.25, -0.07)		0.0404
11	COBB	Born in Australia vs Born overseas	1.15 (-0.90, 3.21)		0.2715
12	Cause_1Rheumatic	No vs Yes	-1.08 (-3.43, 1.26)		0.3654
13	Cause_2Congenital	No vs Yes	-1.96 (-3.85, -0.07)		0.0416
14	Cause_3Arrhythmias	No vs Yes	0.24 (-1.54, 2.02)		0.7935
15	Cause_4Ischaemic	No vs Yes	0.20 (-4.03, 4.43)		0.9256
16	Cause_5Failure	No vs Yes	-2.19 (-4.75, 0.38)		0.0952

Model number	*Predictor*	*Comparison*	*Estimate (95% CI)*	*Comparison P value*	*Global P value*
17	Cause_6Arrest	No vs Yes	1.28 (-3.74, 6.31)		0.6164
18	Cause_7Conduction	No vs Yes	13.44 (0.39, 26.50)		0.0436
19	Cause_8PE	No vs Yes	2.70 (0.06, 5.35)		0.0452
20	Cause_9Hypertension	No vs Yes	-0.42 (-4.65, 3.81)		0.8452
21	Cause_9Hypertension	No vs Yes	-0.42 (-4.65, 3.81)		0.8452
22	Cause_11Other	No vs Yes	1.37 (-0.83, 3.57)		0.2222
23	Age		0.03 (-0.10, 0.16)		0.6728
24	Weight		0.03 (-0.01, 0.07)		0.1999
25	Height		-0.14 (-0.25, -0.03)		0.0162
26	BMI		0.13 (0.02, 0.25)		0.0256
27	SBP		0.01 (-0.04, 0.06)		0.6602
28	DBP		0.00 (-0.05, 0.06)		0.9031
29	ANRQ_score		0.01 (-0.09, 0.10)		0.8875
30	EPPSD_score		0.15 (-0.05, 0.36)		0.1413
31	Maternal_age		0.03 (-0.10, 0.16)		0.6640
32	Gestational_age_admission		-0.05 (-0.24, 0.15)		0.6365
33	Gravida		-0.02 (-0.40, 0.36)		0.9145
34	Parity		-0.25 (-0.70, 0.20)		0.2796
35	Gestational_age		-0.14 (-0.37, 0.08)		0.2033
36	Live_baby_weight per 100g increase		-0.24 (-0.33, -0.17)		<.0001
37	Babys_length_birth		-0.38 (-0.58, -0.17)		0.0003
38	Babys_Head_circ_birth		-0.38 (-0.71, -0.05)		0.0256
39	Apgar_1min		-0.66 (-1.09, -0.23)		0.0027
40	Apgar_5min		-1.02 (-1.73, -0.31)		0.0051
