# Supplementary material for: Adherence to clinical practice guidelines for South Australian pregnant women with cardiac conditions between 2003 and 2013
Source: PLoS One. 2020 Mar 17;15(3):e0230459. doi: 10.1371/journal.pone.0230459 (PMC7077829; doi:10.1371/journal.pone.0230459)
Supplement: S4 Table — (PDF) [file pone.0230459.s004.pdf]

STUDY OF CARDIAC PROBLEMS DURING PREGNANCY – PART 3: UNIVARIATE REGRESSIONS FURTHER CORRECTED

Researcher: Sindy Millington
Statistician: Suzanne Edwards

Date: 23 May 2019

The following outcomes were investigated: preconception, ANC1, ANC2, ANC3, ANCQ, Fetal risk 1_sum, Fetal risk 2_sum, Planned care 1, Planned care 2, Planned care 3, Compromise_sum, Compromise_1Tachycardia, Compromise_2Bradycardia, Compromise_3Respiratory, Compromise_4Changing, Thromboembolism_sum, Thromboembolism_1stockings, Thromboembolism_2heparin, Pain_sum, Pain_1Anaesthetic, Pain_2Epidural, Pain_3Combined, Pain_4Other, Cardiac consultation, and Paediatric neonatal staff. Manage labour resuscitation equipment and Management labour 5 mins cardiac arrest were not included because only for the first, No=2/284 and for the second, No=1/283 so a logistic regression model would not converge.

The following predictors were investigated: Group, Site, High risk 1, High risk 2, High risk 3, High risk 4, High risk 6, High risk 7, High risk 5_sum, High risk 5_1Anaesthetist, High risk 5_2Cardiologist, High risk 5_3Paediatrician, High risk 9_1episiotomy, High risk 9_2forceps, High risk9_4No, team_sum, team_1Obstetrician, team_2Midwife, team_3Cardologist, team_4Physician. team_5Anaesthetist, team_6Intensivist, team_8Other, Cardiac consultation ANRQ score recorded (zero score is No), EPPSD score recorded, Age, Weight, Live baby weight, Baby's length birth, Baby's head circumference birth, ANRQ score (zero score is missing), EPPSD score, Gestational age admission, Gravida, Parity, Gestational age, Apgar 1 minute, and Apgar 5 minutes.

Univariate binary logistic regressions were performed for each binary outcome versus each predictor. Global P values are given in Table 2. Univariate ordinal logistic regressions were performed for each ordinal outcome versus each predictor. Global P values are given in Table 3.

The statistical software used was SAS 9.4 (SAS Institute Inc., Cary, NC, USA).

Table 1. Frequency table of revised groups

Revised_Groups	*Frequency*	*Percent*	*Cumulative Frequency*	*Cumulative Percent*
Acquired	128	47.23	128	47.23
Pre-existant	143	52.77	271	100.00

Table 2. Univariate binary logistic regression results

Model number	*Outcome variable*	*Predictor variable*	*Global P value*
1	preconception	Revised_Groups	0.9959
2	preconception	Site	0.5744
3	preconception	Highrisk1	0.0064
4	preconception	Highrisk2	0.6888
5	preconception	Highrisk3	0.0407
6	preconception	Highrisk4	0.0570
7	preconception	Highrisk6	0.1678
8	preconception	Highrisk7	0.0004
9	preconception	Highrisk5_sum	0.0316
10	preconception	Highrisk5_1Anaesthet	.
11	preconception	Highrisk5_2Cardiolog	0.0373
12	preconception	Highrisk5_3Paediatri	0.0351
13	preconception	Highrisk9_1episiotom	.
14	preconception	Highrisk9_2forceps	0.6189
15	preconception	Highrisk9_4No	0.8050
16	Preconception	team_sum	0.1784
17	Preconception	team_1Obstetrician	0.3052
18	Preconception	team_2Midwife	.
19	Preconception	team_3Cardiologist	0.2038
20	Preconception	team_4Physician	0.1644
21	Preconception	team_5Anaesthetist	0.5981
22	Preconception	team_6Intensivist	0.1556
24	Preconception	team_8Other	0.9373
25	Preconception	Cardiac_consultation	0.0346
26	Preconception	ANRQ_scoreRECORDED	0.2062
27	preconception	EPPSD_scoreRECORDED	0.6733

Model number	*Outcome variable*	*Predictor variable*	*Global P value*
28	preconception	Age	0.5986
29	preconception	Weight	0.8116
31	preconception	BMI	0.4987
34	preconception	Live_baby_weight	0.1138
35	preconception	Babys_length_birth	0.2062
36	preconception	Babys_Head_circ_birth	0.4218
37	preconception	ANRQ_score	0.1449
38	preconception	EPPSD_score	0.5997
39	preconception	Gestational_age_admission	0.3907
40	preconception	Gravida	0.0064
41	preconception	Parity	0.0300
42	preconception	Gestational_age	0.2004
43	preconception	Apgar_1min	0.1904
44	preconception	Apgar_5min	0.1046
1	ANCQ	Revised_Groups	0.0002
2	ANCQ	Site	<.0001
3	ANCQ	Highrisk1	0.2053
4	ANCQ	Highrisk2	<.0001
5	ANCQ	Highrisk3	0.0382
6	ANCQ	Highrisk4	0.0265
6	ANCQ	Highrisk4	0.0265
6	ANCQ	Highrisk4	0.0265
7	ANCQ	Highrisk6	0.1155
8	ANCQ	Highrisk7	0.2681
9	ANCQ	Highrisk5_sum	0.5050
10	ANCQ	Highrisk5_1Anaesthet	0.2252
11	ANCQ	Highrisk5_2Cardiolog	0.5919
12	ANCQ	Highrisk5_3Paediatri	0.6763
13	ANCQ	Highrisk9_1episiotom	.
14	ANCQ	Highrisk9_2forceps	0.6661
15	ANCQ	Highrisk9_4No	0.2434
16	ANCQ	team_sum	0.5665
17	ANCQ	team_1Obstetrician	0.4031
18	ANCQ	team_2Midwife	0.8791
19	ANCQ	team_3Cardiologist	0.4568

Model number	*Outcome variable*	*Predictor variable*	*Global P value*
20	ANCQ	team_4Physician	0.1166
21	ANCQ	team_5Anaesthetist	0.6020
22	ANCQ	team_6Intensivist	0.3457
24	ANCQ	team_8Other	0.8277
25	ANCQ	Cardiac_consultation	0.0359
26	ANCQ	ANRQ_scoreRECORDED	<.0001
27	ANCQ	EPPSD_scoreRECORDED	<.0001
28	ANCQ	Age	0.3341
29	ANCQ	Weight	0.5589
31	ANCQ	BMI	0.1603
34	ANCQ	Live_baby_weight	0.0212
35	ANCQ	Babys_length_birth	0.0120
36	ANCQ	Babys_Head_circ_birth	0.9100
37	ANCQ	ANRQ_score	0.3664
38	ANCQ	EPPSD_score	0.1680
39	ANCQ	Gestational_age_admission	0.4354
40	ANCQ	Gravida	0.7751
41	ANCQ	Parity	0.5044
42	ANCQ	Gestational_age	0.2061
43	ANCQ	Apgar_1min	0.3682
44	ANCQ	Apgar_5min	0.4192
1	FetalRisk2_sum	Revised_Groups	0.4736
2	FetalRisk2_sum	Site	0.8924
3	FetalRisk2_sum	Highrisk1	0.6230
4	FetalRisk2_sum	Highrisk2	0.6500
5	FetalRisk2_sum	Highrisk3	0.6258
6	FetalRisk2_sum	Highrisk4	<.0001
7	FetalRisk2_sum	Highrisk6	.
8	FetalRisk2_sum	Highrisk7	0.4611
9	FetalRisk2_sum	Highrisk5_sum	0.9360
10	FetalRisk2_sum	Highrisk5_1Anaesthet	0.4218
11	FetalRisk2_sum	Highrisk5_2Cardiolog	0.5902
12	FetalRisk2_sum	Highrisk5_3Paediatri	0.5192
13	FetalRisk2_sum	Highrisk9_1episiotom	0.9456
14	FetalRisk2_sum	Highrisk9_2forceps	0.3585

Model number	*Outcome variable*	*Predictor variable*	*Global P value*
15	FetalRisk2_sum	Highrisk9_4No	0.6821
16	FetalRisk2_sum	team_sum	0.8127
17	FetalRisk2_sum	team_1Obstetrician	0.8285
18	FetalRisk2_sum	team_2Midwife	0.7262
19	FetalRisk2_sum	team_3Cardiologist	0.8048
20	FetalRisk2_sum	team_4Physician	0.4156
21	FetalRisk2_sum	team_5Anaesthetist	0.8573
22	FetalRisk2_sum	team_6Intensivist	0.6392
24	FetalRisk2_sum	team_8Other	0.9889
25	FetalRisk2_sum	Cardiac_consultation	0.2012
26	FetalRisk2_sum	ANRQ_scoreRECORDED	0.7571
27	FetalRisk2_sum	EPPSD_scoreRECORDED	0.5739
28	FetalRisk2_sum	Age	0.1355
29	FetalRisk2_sum	Weight	0.9198
31	FetalRisk2_sum	BMI	0.9669
34	FetalRisk2_sum	Live_baby_weight	0.7901
35	FetalRisk2_sum	Babys_length_birth	0.6583
36	FetalRisk2_sum	Babys_Head_circ_birth	0.7096
37	FetalRisk2_sum	ANRQ_score	0.8734
38	FetalRisk2_sum	EPPSD_score	0.8194
39	FetalRisk2_sum	Gestational_age_admission	0.3897
40	FetalRisk2_sum	Gravida	0.6335
41	FetalRisk2_sum	Parity	0.5617
42	FetalRisk2_sum	Gestational_age	0.4715
43	FetalRisk2_sum	Apgar_1min	0.3404
44	FetalRisk2_sum	Apgar_5min	0.8797
1	Plannedcare1	Revised_Groups	0.1902
2	Plannedcare1	Site	0.2330
3	Plannedcare1	Highrisk1	0.0032
4	Plannedcare1	Highrisk2	0.0740
5	Plannedcare1	Highrisk3	<.0001
6	Plannedcare1	Highrisk4	<.0001
7	Plannedcare1	Highrisk6	0.0661
8	Plannedcare1	Highrisk7	0.0002
9	Plannedcare1	Highrisk5_sum	0.0326

Model number	*Outcome variable*	*Predictor variable*	*Global P value*
10	Plannedcare1	Highrisk5_1Anaesthet	0.0036
11	Plannedcare1	Highrisk5_2Cardiolog	0.1872
12	Plannedcare1	Highrisk5_3Paediatri	0.1486
13	Plannedcare1	Highrisk9_1episiotom	0.0253
14	Plannedcare1	Highrisk9_2forceps	0.2251
15	Plannedcare1	Highrisk9_4No	0.9771
16	Plannedcare1	team_sum	0.0147
17	Plannedcare1	team_1Obstetrician	0.0139
18	Plannedcare1	team_2Midwife	0.6513
19	Plannedcare1	team_3Cardiologist	.
20	Plannedcare1	team_4Physician	0.6317
21	Plannedcare1	team_5Anaesthetist	0.0145
22	Plannedcare1	team_6Intensivist	.
24	Plannedcare1	team_8Other	0.9465
25	Plannedcare1	Cardiac_consultation	<.0001
26	Plannedcare1	ANRQ_scoreRECORDED	0.9440
27	Plannedcare1	EPPSD_scoreRECORDED	0.2897
28	Plannedcare1	Age	0.3543
29	Plannedcare1	Weight	0.9166
31	Plannedcare1	BMI	0.7396
34	Plannedcare1	Live_baby_weight	0.6693
35	Plannedcare1	Babys_length_birth	0.6329
36	Plannedcare1	Babys_Head_circ_birth	0.3931
37	Plannedcare1	ANRQ_score	0.6211
38	Plannedcare1	EPPSD_score	0.9771
39	Plannedcare1	Gestational_age_admission	0.9473
40	Plannedcare1	Gravida	0.1260
41	Plannedcare1	Parity	0.1626
42	Plannedcare1	Gestational_age	0.7758
43	Plannedcare1	Apgar_1min	0.1830
44	Plannedcare1	Apgar_5min	0.1293
1	Plannedcare2	Revised_Groups	0.4320
2	Plannedcare2	Site	0.8166
3	Plannedcare2	Highrisk1	0.0172
4	Plannedcare2	Highrisk2	0.2999

Model number	*Outcome variable*	*Predictor variable*	*Global P value*
5	Plannedcare2	Highrisk3	.
6	Plannedcare2	Highrisk4	0.0735
7	Plannedcare2	Highrisk6	0.5601
8	Plannedcare2	Highrisk7	.
9	Plannedcare2	Highrisk5_sum	<.0001
10	Plannedcare2	Highrisk5_1Anaesthet	.
11	Plannedcare2	Highrisk5_2Cardiolog	.
12	Plannedcare2	Highrisk5_3Paediatri	0.1864
13	Plannedcare2	Highrisk9_1episiotom	.
14	Plannedcare2	Highrisk9_2forceps	.
15	Plannedcare2	Highrisk9_4No	.
16	Plannedcare2	team_sum	0.3790
17	Plannedcare2	team_1Obstetrician	0.5975
18	Plannedcare2	team_2Midwife	0.1070
19	Plannedcare2	team_3Cardiologist	0.4169
20	Plannedcare2	team_4Physician	0.7781
21	Plannedcare2	team_5Anaesthetist	0.1823
22	Plannedcare2	team_6Intensivist	0.0793
24	Plannedcare2	team_8Other	.
25	Plannedcare2	Cardiac_consultation	.
26	Plannedcare2	ANRQ_scoreRECORDED	0.5502
27	Plannedcare2	EPPSD_scoreRECORDED	0.9395
28	Plannedcare2	Age	0.4794
29	Plannedcare2	Weight	0.6809
31	Plannedcare2	BMI	0.8773
34	Plannedcare2	Live_baby_weight	0.0373
35	Plannedcare2	Babys_length_birth	0.9338
36	Plannedcare2	Babys_Head_circ_birth	0.9342
37	Plannedcare2	ANRQ_score	0.5530
38	Plannedcare2	EPPSD_score	0.4655
39	Plannedcare2	Gestational_age_admission	0.1911
40	Plannedcare2	Gravida	0.2525
41	Plannedcare2	Parity	0.0137
42	Plannedcare2	Gestational_age	0.0227
43	Plannedcare2	Apgar_1min	0.1772

Model number	*Outcome variable*	*Predictor variable*	*Global P value*
44	Plannedcare2	Apgar_5min	0.2124
1	Plannedcare3x	Revised_Groups	0.9368
2	Plannedcare3x	Site	0.3874
3	Plannedcare3x	Highrisk1	<.0001
4	Plannedcare3x	Highrisk2	0.4857
5	Plannedcare3x	Highrisk3	0.0034
6	Plannedcare3x	Highrisk4	<.0001
7	Plannedcare3x	Highrisk6	0.0012
8	Plannedcare3x	Highrisk7	0.1221
9	Plannedcare3x	Highrisk5_sum	<.0001
10	Plannedcare3x	Highrisk5_1Anaesthet	0.0166
11	Plannedcare3x	Highrisk5_2Cardiolog	0.0205
12	Plannedcare3x	Highrisk5_3Paediatri	<.0001
13	Plannedcare3x	Highrisk9_1episiotom	.
14	Plannedcare3x	Highrisk9_2forceps	0.7089
15	Plannedcare3x	Highrisk9_4No	0.0019
16	Plannedcare3x	team_sum	<.0001
17	Plannedcare3x	team_1Obstetrician	<.0001
18	Plannedcare3x	team_2Midwife	0.0408
19	Plannedcare3x	team_3Cardiologist	0.2994
20	Plannedcare3x	team_4Physician	<.0001
21	Plannedcare3x	team_5Anaesthetist	<.0001
22	Plannedcare3x	team_6Intensivist	0.0035
24	Plannedcare3x	team_8Other	0.3503
25	Plannedcare3x	Cardiac_consultation	0.0409
26	Plannedcare3x	ANRQ_scoreRECORDED	0.1078
27	Plannedcare3x	EPPSD_scoreRECORDED	0.4008
28	Plannedcare3x	Age	0.4435
29	Plannedcare3x	Weight	0.4122
31	Plannedcare3x	BMI	0.0511
34	Plannedcare3x	Live_baby_weight	0.0001
35	Plannedcare3x	Babys_length_birth	0.0169
36	Plannedcare3x	Babys_Head_circ_birth	0.0350
37	Plannedcare3x	ANRQ_score	0.6978
38	Plannedcare3x	EPPSD_score	0.2757

Model number	*Outcome variable*	*Predictor variable*	*Global P value*
39	Plannedcare3x	Gestational_age_admission	0.0016
40	Plannedcare3x	Gravida	0.4471
41	Plannedcare3x	Parity	0.6649
42	Plannedcare3x	Gestational_age	<.0001
43	Plannedcare3x	Apgar_1min	0.0002
44	Plannedcare3x	Apgar_5min	0.0013
1	compromise_1Tachycardia	Revised_Groups	0.9999
3	compromise_1Tachycardia	Highrisk1	.
4	compromise_1Tachycardia	Highrisk2	.
5	compromise_1Tachycardia	Highrisk3	.
5	compromise_1Tachycardia	Highrisk3	.
7	compromise_1Tachycardia	Highrisk6	.
8	compromise_1Tachycardia	Highrisk7	1.0000
9	compromise_1Tachycardia	Highrisk5_sum	1.0000
10	compromise_1Tachycardia	Highrisk5_1Anaesthet	.
11	compromise_1Tachycardia	Highrisk5_2Cardiolog	.
12	compromise_1Tachycardia	Highrisk5_3Paediatri	.
13	compromise_1Tachycardia	Highrisk9_1episiotom	0.9999
14	compromise_1Tachycardia	Highrisk9_2forceps	1.0000
15	compromise_1Tachycardia	Highrisk9_4No	1.0000
44	Plannedcare3x	Apgar_5min	0.0013
17	compromise_1Tachycardia	team_1Obstetrician	0.9999
18	compromise_1Tachycardia	team_2Midwife	.
19	compromise_1Tachycardia	team_3Cardiologist	.
20	compromise_1Tachycardia	team_4Physician	0.9999
21	compromise_1Tachycardia	team_5Anaesthetist	0.9999
22	compromise_1Tachycardia	team_6Intensivist	.
24	compromise_1Tachycardia	team_8Other	.
25	compromise_1Tachycardia	Cardiac_consultation	0.9999
26	compromise_1Tachycardia	ANRQ_scoreRECORDED	0.9999
27	compromise_1Tachycardia	EPPSD_scoreRECORDED	.
28	compromise_1Tachycardia	Age	0.3991
29	compromise_1Tachycardia	Weight	0.1339
31	compromise_1Tachycardia	BMI	0.2292
34	compromise_1Tachycardia	Live_baby_weight	0.2020

Model number	*Outcome variable*	*Predictor variable*	*Global P value*
35	compromise_1Tachycardia	Babys_length_birth	0.2999
36	compromise_1Tachycardia	Babys_Head_circ_birth	0.4368
.		Babys_Head_circ_birt	0.4368
36	compromise_1Tachycardia	Babys_Head_circ_birth	.
38	compromise_1Tachycardia	EPPSD_score	1.0000
39	compromise_1Tachycardia	Gestational_age_admission	0.1841
40	compromise_1Tachycardia	Gravida	0.5801
41	compromise_1Tachycardia	Parity	0.7616
42	compromise_1Tachycardia	Gestational_age	0.1081
43	compromise_1Tachycardia	Apgar_1min	0.5567
43	compromise_1Tachycardia	Apgar_1min	0.5567
1	compromise_2Bradycardia	Revised_Groups	0.9999
2	compromise_2Bradycardia	Site	<.0001
3	compromise_2Bradycardia	Highrisk1	.
4	compromise_2Bradycardia	Highrisk2	0.8374
5	compromise_2Bradycardia	Highrisk3	0.0936
5	compromise_2Bradycardia	Highrisk3	0.0936
7	compromise_2Bradycardia	Highrisk6	0.2630
8	compromise_2Bradycardia	Highrisk7	.
9	compromise_2Bradycardia	Highrisk5_sum	1.0000
10	compromise_2Bradycardia	Highrisk5_1Anaesthet	.
11	compromise_2Bradycardia	Highrisk5_2Cardiolog	.
12	compromise_2Bradycardia	Highrisk5_3Paediatri	.
13	compromise_2Bradycardia	Highrisk9_1episiotom	1.0000
14	compromise_2Bradycardia	Highrisk9_2forceps	1.0000
15	compromise_2Bradycardia	Highrisk9_4No	.
16	compromise_2Bradycardia	team_sum	0.3825
17	compromise_2Bradycardia	team_1Obstetrician	.
18	compromise_2Bradycardia	team_2Midwife	.
19	compromise_2Bradycardia	team_3Cardiologist	.
20	compromise_2Bradycardia	team_4Physician	0.9999
21	compromise_2Bradycardia	team_5Anaesthetist	0.5442
22	compromise_2Bradycardia	team_6Intensivist	.
24	compromise_2Bradycardia	team_8Other	.
25	compromise_2Bradycardia	Cardiac_consultation	.

Model number	*Outcome variable*	*Predictor variable*	*Global P value*
26	compromise_2Bradycardia	ANRQ_scoreRECORDED	0.7739
27	compromise_2Bradycardia	EPPSD_scoreRECORDED	0.7739
28	compromise_2Bradycardia	Age	0.0491
29	compromise_2Bradycardia	Weight	0.5421
31	compromise_2Bradycardia	BMI	0.2235
34	compromise_2Bradycardia	Live_baby_weight	0.1913
35	compromise_2Bradycardia	Babys_length_birth	0.3584
36	compromise_2Bradycardia	Babys_Head_circ_birth	0.7262
37	compromise_2Bradycardia	ANRQ_score	0.5055
38	compromise_2Bradycardia	EPPSD_score	0.0998
39	compromise_2Bradycardia	Gestational_age_admission	0.1608
40	compromise_2Bradycardia	Gravida	0.2407
41	compromise_2Bradycardia	Parity	0.5458
42	compromise_2Bradycardia	Gestational_age	0.1721
43	compromise_2Bradycardia	Apgar_1min	0.5282
44	compromise_2Bradycardia	Apgar_5min	0.2710
1	compromise_3Respiratory	Revised_Groups	0.1310
2	compromise_3Respiratory	Site	<.0001
3	compromise_3Respiratory	Highrisk1	0.7581
4	compromise_3Respiratory	Highrisk2	0.3447
5	compromise_3Respiratory	Highrisk3	0.0170
6	compromise_3Respiratory	Highrisk4	<.0001
7	compromise_3Respiratory	Highrisk6	0.5457
8	compromise_3Respiratory	Highrisk7	0.2475
9	compromise_3Respiratory	Highrisk5_sum	0.5366
10	compromise_3Respiratory	Highrisk5_1Anaesthet	0.0671
11	compromise_3Respiratory	Highrisk5_2Cardiolog	.
12	compromise_3Respiratory	Highrisk5_3Paediatri	0.4914
13	compromise_3Respiratory	Highrisk9_1episiotom	0.0044
14	compromise_3Respiratory	Highrisk9_2forceps	0.1907
15	compromise_3Respiratory	Highrisk9_4No	0.1249
16	compromise_3Respiratory	team_sum	0.0544
17	compromise_3Respiratory	team_1Obstetrician	0.1225
18	compromise_3Respiratory	team_2Midwife	.
19	compromise_3Respiratory	team_3Cardiologist	.

Model number	*Outcome variable*	*Predictor variable*	*Global P value*
20	compromise_3Respiratory	team_4Physician	0.1727
21	compromise_3Respiratory	team_5Anaesthetist	0.2548
22	compromise_3Respiratory	team_6Intensivist	.
24	compromise_3Respiratory	team_8Other	.
25	compromise_3Respiratory	Cardiac_consultation	0.2970
26	compromise_3Respiratory	ANRQ_scoreRECORDED	0.9458
27	compromise_3Respiratory	EPPSD_scoreRECORDED	0.3441
28	compromise_3Respiratory	Age	0.0502
29	compromise_3Respiratory	Weight	0.0901
31	compromise_3Respiratory	BMI	0.6285
34	compromise_3Respiratory	Live_baby_weight	0.1662
35	compromise_3Respiratory	Babys_length_birth	0.5267
36	compromise_3Respiratory	Babys_Head_circ_birth	0.8825
37	compromise_3Respiratory	ANRQ_score	0.6331
38	compromise_3Respiratory	EPPSD_score	0.0998
39	compromise_3Respiratory	Gestational_age_admission	0.0858
40	compromise_3Respiratory	Gravida	0.6049
41	compromise_3Respiratory	Parity	0.4934
42	compromise_3Respiratory	Gestational_age	0.0533
43	compromise_3Respiratory	Apgar_1min	0.4477
44	compromise_3Respiratory	Apgar_5min	0.0131
1	compromise_4Changing	Revised_Groups	0.8752
2	compromise_4Changing	Site	<.0001
3	compromise_4Changing	Highrisk1	0.5850
4	compromise_4Changing	Highrisk2	0.2848
5	compromise_4Changing	Highrisk3	0.0251
6	compromise_4Changing	Highrisk4	<.0001
7	compromise_4Changing	Highrisk6	0.8862
8	compromise_4Changing	Highrisk7	0.6465
9	compromise_4Changing	Highrisk5_sum	0.7750
10	compromise_4Changing	Highrisk5_1Anaesthet	0.3494
11	compromise_4Changing	Highrisk5_2Cardiolog	0.7194
12	compromise_4Changing	Highrisk5_3Paediatri	0.6880
13	compromise_4Changing	Highrisk9_1episiotom	0.0011
14	compromise_4Changing	Highrisk9_2forceps	0.4834

Model number	*Outcome variable*	*Predictor variable*	*Global P value*
15	compromise_4Changing	Highrisk9_4No	0.7104
16	compromise_4Changing	team_sum	0.5753
17	compromise_4Changing	team_1Obstetrician	0.4875
18	compromise_4Changing	team_2Midwife	.
19	compromise_4Changing	team_3Cardiologist	0.8979
20	compromise_4Changing	team_4Physician	0.9069
21	compromise_4Changing	team_5Anaesthetist	0.4937
22	compromise_4Changing	team_6Intensivist	0.8510
24	compromise_4Changing	team_8Other	.
25	compromise_4Changing	Cardiac_consultation	0.7432
26	compromise_4Changing	ANRQ_scoreRECORDED	0.7824
27	compromise_4Changing	EPPSD_scoreRECORDED	0.3312
28	compromise_4Changing	Age	0.3334
29	compromise_4Changing	Weight	0.4179
31	compromise_4Changing	BMI	0.9420
34	compromise_4Changing	Live_baby_weight	0.6195
35	compromise_4Changing	Babys_length_birth	0.6210
36	compromise_4Changing	Babys_Head_circ_birth	0.2864
37	compromise_4Changing	ANRQ_score	0.4206
38	compromise_4Changing	EPPSD_score	0.0289
39	compromise_4Changing	Gestational_age_admission	0.3465
40	compromise_4Changing	Gravida	0.4391
41	compromise_4Changing	Parity	0.8540
42	compromise_4Changing	Gestational_age	0.3385
43	compromise_4Changing	Apgar_1min	0.8849
44	compromise_4Changing	Apgar_5min	0.3269
1	thromboembolism_sum	Revised_Groups	0.0229
2	thromboembolism_sum	Site	0.0148
3	thromboembolism_sum	Highrisk1	0.0531
4	thromboembolism_sum	Highrisk2	0.0838
5	thromboembolism_sum	Highrisk3	0.5769
6	thromboembolism_sum	Highrisk4	<.0001
7	thromboembolism_sum	Highrisk6	0.1041
8	thromboembolism_sum	Highrisk7	0.0833
9	thromboembolism_sum	Highrisk5_sum	0.5007

Model number	*Outcome variable*	*Predictor variable*	*Global P value*
10	thromboembolism_sum	Highrisk5_1Anaesthet	.
11	thromboembolism_sum	Highrisk5_2Cardiolog	0.5964
12	thromboembolism_sum	Highrisk5_3Paediatri	0.3058
13	thromboembolism_sum	Highrisk9_1episiotom	.
14	thromboembolism_sum	Highrisk9_2forceps	0.4560
15	thromboembolism_sum	Highrisk9_4No	0.2840
16	thromboembolism_sum	team_sum	0.0105
17	thromboembolism_sum	team_1Obstetrician	0.0084
18	thromboembolism_sum	team_2Midwife	0.6977
19	thromboembolism_sum	team_3Cardiologist	0.5441
20	thromboembolism_sum	team_4Physician	0.7079
21	thromboembolism_sum	team_5Anaesthetist	0.0119
22	thromboembolism_sum	team_6Intensivist	0.0250
24	thromboembolism_sum	team_8Other	.
25	thromboembolism_sum	Cardiac_consultation	0.1039
26	thromboembolism_sum	ANRQ_scoreRECORDED	0.6525
27	thromboembolism_sum	EPPSD_scoreRECORDED	0.9157
28	thromboembolism_sum	Age	0.1472
29	thromboembolism_sum	Weight	0.1049
31	thromboembolism_sum	BMI	0.3093
34	thromboembolism_sum	Live_baby_weight	0.0419
35	thromboembolism_sum	Babys_length_birth	0.1773
36	thromboembolism_sum	Babys_Head_circ_birth	0.2620
37	thromboembolism_sum	ANRQ_score	0.8864
38	thromboembolism_sum	EPPSD_score	0.1926
39	thromboembolism_sum	Gestational_age_admission	0.4325
40	thromboembolism_sum	Gravida	0.0966
41	thromboembolism_sum	Parity	0.1144
42	thromboembolism_sum	Gestational_age	0.7165
43	thromboembolism_sum	Apgar_1min	0.2286
44	thromboembolism_sum	Apgar_5min	0.1340
1	thromboembolism_1stockings	Revised_Groups	0.1828
2	thromboembolism_1stockings	Site	0.0661
3	thromboembolism_1stockings	Highrisk1	0.1214
4	thromboembolism_1stockings	Highrisk2	0.4971

Model number	*Outcome variable*	*Predictor variable*	*Global P value*
5	thromboembolism_1stockings	Highrisk3	0.7703
6	thromboembolism_1stockings	Highrisk4	<.0001
6	thromboembolism_1stockings	Highrisk4	<.0001
6	thromboembolism_1stockings	Highrisk4	<.0001
7	thromboembolism_1stockings	Highrisk6	0.0943
8	thromboembolism_1stockings	Highrisk7	0.3623
9	thromboembolism_1stockings	Highrisk5_sum	0.5503
10	thromboembolism_1stockings	Highrisk5_1Anaesthet	.
11	thromboembolism_1stockings	Highrisk5_2Cardiolog	0.7500
12	thromboembolism_1stockings	Highrisk5_3Paediatri	0.1220
13	thromboembolism_1stockings	Highrisk9_1episiotom	.
14	thromboembolism_1stockings	Highrisk9_2forceps	0.2455
15	thromboembolism_1stockings	Highrisk9_4No	0.8404
16	thromboembolism_1stockings	team_sum	0.0461
17	thromboembolism_1stockings	team_1Obstetrician	0.1328
18	thromboembolism_1stockings	team_2Midwife	0.7169
19	thromboembolism_1stockings	team_3Cardiologist	0.3904
20	thromboembolism_1stockings	team_4Physician	0.8482
21	thromboembolism_1stockings	team_5Anaesthetist	0.0279
22	thromboembolism_1stockings	team_6Intensivist	0.0206
24	thromboembolism_1stockings	team_8Other	.
25	thromboembolism_1stockings	Cardiac_consultation	0.9186
26	thromboembolism_1stockings	ANRQ_scoreRECORDED	0.3958
27	thromboembolism_1stockings	EPPSD_scoreRECORDED	0.5448
28	thromboembolism_1stockings	Age	0.2137
29	thromboembolism_1stockings	Weight	0.0478
31	thromboembolism_1stockings	BMI	0.2059
34	thromboembolism_1stockings	Live_baby_weight	0.0132
35	thromboembolism_1stockings	Babys_length_birth	0.1577
36	thromboembolism_1stockings	Babys_Head_circ_birth	0.2523
37	thromboembolism_1stockings	ANRQ_score	0.3812
38	thromboembolism_1stockings	EPPSD_score	0.5188
39	thromboembolism_1stockings	Gestational_age_admission	0.2586
40	thromboembolism_1stockings	Gravida	0.6399
41	thromboembolism_1stockings	Parity	0.9268

Model number	*Outcome variable*	*Predictor variable*	*Global P value*
42	thromboembolism_1stockings	Gestational_age	0.4309
43	thromboembolism_1stockings	Apgar_1min	0.2211
44	thromboembolism_1stockings	Apgar_5min	0.1033
1	thromboembolism_2heparin	Revised_Groups	0.1462
2	thromboembolism_2heparin	Site	0.0802
3	thromboembolism_2heparin	Highrisk1	0.4845
4	thromboembolism_2heparin	Highrisk2	0.1100
5	thromboembolism_2heparin	Highrisk3	0.6811
6	thromboembolism_2heparin	Highrisk4	<.0001
7	thromboembolism_2heparin	Highrisk6	0.8558
8	thromboembolism_2heparin	Highrisk7	0.1246
9	thromboembolism_2heparin	Highrisk5_sum	0.9537
10	thromboembolism_2heparin	Highrisk5_1Anaesthet	.
11	thromboembolism_2heparin	Highrisk5_2Cardiolog	0.7373
12	thromboembolism_2heparin	Highrisk5_3Paediatri	0.5457
13	thromboembolism_2heparin	Highrisk9_1episiotom	.
14	thromboembolism_2heparin	Highrisk9_2forceps	0.5247
15	thromboembolism_2heparin	Highrisk9_4No	0.0699
16	thromboembolism_2heparin	team_sum	0.3191
17	thromboembolism_2heparin	team_1Obstetrician	0.0482
18	thromboembolism_2heparin	team_2Midwife	0.9539
19	thromboembolism_2heparin	team_3Cardiologist	0.7408
20	thromboembolism_2heparin	team_4Physician	0.4167
21	thromboembolism_2heparin	team_5Anaesthetist	0.3662
22	thromboembolism_2heparin	team_6Intensivist	0.5963
24	thromboembolism_2heparin	team_8Other	.
25	thromboembolism_2heparin	Cardiac_consultation	0.0114
26	thromboembolism_2heparin	ANRQ_scoreRECORDED	0.5920
27	thromboembolism_2heparin	EPPSD_scoreRECORDED	0.3178
28	thromboembolism_2heparin	Age	0.7045
29	thromboembolism_2heparin	Weight	0.6728
31	thromboembolism_2heparin	BMI	0.7646
34	thromboembolism_2heparin	Live_baby_weight	0.5877
35	thromboembolism_2heparin	Babys_length_birth	0.9831
36	thromboembolism_2heparin	Babys_Head_circ_birth	0.9697

Model number	*Outcome variable*	*Predictor variable*	*Global P value*
37	thromboembolism_2heparin	ANRQ_score	0.1380
38	thromboembolism_2heparin	EPPSD_score	0.2508
39	thromboembolism_2heparin	Gestational_age_admission	0.6548
40	thromboembolism_2heparin	Gravida	0.0792
41	thromboembolism_2heparin	Parity	0.0351
42	thromboembolism_2heparin	Gestational_age	0.5651
43	thromboembolism_2heparin	Apgar_1min	0.9549
44	thromboembolism_2heparin	Apgar_5min	0.9098
1	Pain_1Anaesthetic	Revised_Groups	0.0550
2	Pain_1Anaesthetic	Site	0.4731
3	Pain_1Anaesthetic	Highrisk1	0.0002
4	Pain_1Anaesthetic	Highrisk2	0.5434
5	Pain_1Anaesthetic	Highrisk3	0.3008
6	Pain_1Anaesthetic	Highrisk4	0.1538
6	Pain_1Anaesthetic	Highrisk4	0.1538
6	Pain_1Anaesthetic	Highrisk4	0.1538
7	Pain_1Anaesthetic	Highrisk6	0.0241
8	Pain_1Anaesthetic	Highrisk7	0.8940
9	Pain_1Anaesthetic	Highrisk5_sum	0.3133
10	Pain_1Anaesthetic	Highrisk5_1Anaesthet	1.0000
11	Pain_1Anaesthetic	Highrisk5_2Cardiolog	0.5887
12	Pain_1Anaesthetic	Highrisk5_3Paediatri	0.0417
13	Pain_1Anaesthetic	Highrisk9_1episiotom	0.6688
14	Pain_1Anaesthetic	Highrisk9_2forceps	0.0776
15	Pain_1Anaesthetic	Highrisk9_4No	0.1983
16	Pain_1Anaesthetic	team_sum	0.0105
17	Pain_1Anaesthetic	team_1Obstetrician	0.0704
18	Pain_1Anaesthetic	team_2Midwife	0.2045
19	Pain_1Anaesthetic	team_3Cardiologist	0.0553
20	Pain_1Anaesthetic	team_4Physician	0.3941
21	Pain_1Anaesthetic	team_5Anaesthetist	0.0242
22	Pain_1Anaesthetic	team_6Intensivist	0.0669
24	Pain_1Anaesthetic	team_8Other	0.5557
25	Pain_1Anaesthetic	Cardiac_consultation	0.3211
26	Pain_1Anaesthetic	ANRQ_scoreRECORDED	0.0767

Model number	*Outcome variable*	*Predictor variable*	*Global P value*
27	Pain_1Anaesthetic	EPPSD_scoreRECORDED	0.6117
28	Pain_1Anaesthetic	Age	0.6775
29	Pain_1Anaesthetic	Weight	0.1274
31	Pain_1Anaesthetic	BMI	0.1755
34	Pain_1Anaesthetic	Live_baby_weight	<.0001
35	Pain_1Anaesthetic	Babys_length_birth	0.0599
36	Pain_1Anaesthetic	Babys_Head_circ_birth	0.0053
37	Pain_1Anaesthetic	ANRQ_score	0.7158
38	Pain_1Anaesthetic	EPPSD_score	0.2038
39	Pain_1Anaesthetic	Gestational_age_admission	0.0220
40	Pain_1Anaesthetic	Gravida	0.0926
41	Pain_1Anaesthetic	Parity	0.0601
42	Pain_1Anaesthetic	Gestational_age	0.0049
43	Pain_1Anaesthetic	Apgar_1min	0.3058
44	Pain_1Anaesthetic	Apgar_5min	0.4685
1	Pain_2Epidural	Revised_Groups	0.0484
2	Pain_2Epidural	Site	0.7973
3	Pain_2Epidural	Highrisk1	0.1834
4	Pain_2Epidural	Highrisk2	0.4142
5	Pain_2Epidural	Highrisk3	0.0095
6	Pain_2Epidural	Highrisk4	<.0001
7	Pain_2Epidural	Highrisk6	0.1380
8	Pain_2Epidural	Highrisk7	0.3903
9	Pain_2Epidural	Highrisk5_sum	0.5175
10	Pain_2Epidural	Highrisk5_1Anaesthet	0.3975
11	Pain_2Epidural	Highrisk5_2Cardiolog	0.7745
12	Pain_2Epidural	Highrisk5_3Paediatri	0.9426
13	Pain_2Epidural	Highrisk9_1episiotom	0.0148
14	Pain_2Epidural	Highrisk9_2forceps	0.0403
15	Pain_2Epidural	Highrisk9_4No	0.4657
16	Pain_2Epidural	team_sum	0.0199
17	Pain_2Epidural	team_1Obstetrician	0.1893
18	Pain_2Epidural	team_2Midwife	0.5935
19	Pain_2Epidural	team_3Cardiologist	0.8569
20	Pain_2Epidural	team_4Physician	0.3768

Model number	*Outcome variable*	*Predictor variable*	*Global P value*
21	Pain_2Epidural	team_5Anaesthetist	0.0002
22	Pain_2Epidural	team_6Intensivist	0.9066
24	Pain_2Epidural	team_8Other	.
25	Pain_2Epidural	Cardiac_consultation	0.2784
26	Pain_2Epidural	ANRQ_scoreRECORDED	0.5580
27	Pain_2Epidural	EPPSD_scoreRECORDED	0.6287
28	Pain_2Epidural	Age	0.8677
29	Pain_2Epidural	Weight	0.4961
31	Pain_2Epidural	BMI	0.2991
34	Pain_2Epidural	Live_baby_weight	0.8189
35	Pain_2Epidural	Babys_length_birth	0.6755
36	Pain_2Epidural	Babys_Head_circ_birth	0.5328
37	Pain_2Epidural	ANRQ_score	0.6272
38	Pain_2Epidural	EPPSD_score	0.1705
39	Pain_2Epidural	Gestational_age_admission	0.0415
40	Pain_2Epidural	Gravida	0.1104
41	Pain_2Epidural	Parity	0.1361
42	Pain_2Epidural	Gestational_age	0.2341
43	Pain_2Epidural	Apgar_1min	0.0006
44	Pain_2Epidural	Apgar_5min	0.0012
1	Pain_3Combined	Revised_Groups	0.9854
2	Pain_3Combined	Site	0.0501
3	Pain_3Combined	Highrisk1	0.5614
4	Pain_3Combined	Highrisk2	0.9640
5	Pain_3Combined	Highrisk3	0.0183
6	Pain_3Combined	Highrisk4	<.0001
7	Pain_3Combined	Highrisk6	0.1420
8	Pain_3Combined	Highrisk7	0.4533
9	Pain_3Combined	Highrisk5_sum	0.0413
10	Pain_3Combined	Highrisk5_1Anaesthet	0.2690
11	Pain_3Combined	Highrisk5_2Cardiolog	0.1024
12	Pain_3Combined	Highrisk5_3Paediatri	0.0274
13	Pain_3Combined	Highrisk9_1episiotom	.
14	Pain_3Combined	Highrisk9_2forceps	0.4581
15	Pain_3Combined	Highrisk9_4No	0.0175

Model number	*Outcome variable*	*Predictor variable*	*Global P value*
16	Pain_3Combined	team_sum	<.0001
17	Pain_3Combined	team_1Obstetrician	<.0001
18	Pain_3Combined	team_2Midwife	0.6702
19	Pain_3Combined	team_3Cardiologist	0.8801
20	Pain_3Combined	team_4Physician	0.0013
21	Pain_3Combined	team_5Anaesthetist	0.0028
22	Pain_3Combined	team_6Intensivist	0.0770
24	Pain_3Combined	team_8Other	0.4806
25	Pain_3Combined	Cardiac_consultation	0.0365
26	Pain_3Combined	ANRQ_scoreRECORDED	0.2106
27	Pain_3Combined	EPPSD_scoreRECORDED	0.2882
28	Pain_3Combined	Age	0.3734
29	Pain_3Combined	Weight	0.3358
31	Pain_3Combined	BMI	0.3014
34	Pain_3Combined	Live_baby_weight	0.0940
35	Pain_3Combined	Babys_length_birth	0.0770
36	Pain_3Combined	Babys_Head_circ_birth	0.1409
37	Pain_3Combined	ANRQ_score	0.1839
38	Pain_3Combined	EPPSD_score	0.2854
39	Pain_3Combined	Gestational_age_admission	0.2992
40	Pain_3Combined	Gravida	0.6209
41	Pain_3Combined	Parity	0.9645
42	Pain_3Combined	Gestational_age	0.0123
43	Pain_3Combined	Apgar_1min	0.1659
44	Pain_3Combined	Apgar_5min	0.3378
1	Pain_4Other	Revised_Groups	0.0668
2	Pain_4Other	Site	0.9900
3	Pain_4Other	Highrisk1	0.0054
4	Pain_4Other	Highrisk2	0.8233
5	Pain_4Other	Highrisk3	0.9198
6	Pain_4Other	Highrisk4	<.0001
7	Pain_4Other	Highrisk6	0.8731
8	Pain_4Other	Highrisk7	0.8057
9	Pain_4Other	Highrisk5_sum	0.8733
10	Pain_4Other	Highrisk5_1Anaesthet	0.6690

Model number	*Outcome variable*	*Predictor variable*	*Global P value*
11	Pain_4Other	Highrisk5_2Cardiolog	0.8039
12	Pain_4Other	Highrisk5_3Paediatri	0.6067
13	Pain_4Other	Highrisk9_1episiotom	0.1021
14	Pain_4Other	Highrisk9_2forceps	0.1163
15	Pain_4Other	Highrisk9_4No	0.3724
16	Pain_4Other	team_sum	0.7442
17	Pain_4Other	team_1Obstetrician	0.8440
18	Pain_4Other	team_2Midwife	0.4548
19	Pain_4Other	team_3Cardiologist	0.0567
20	Pain_4Other	team_4Physician	0.7860
21	Pain_4Other	team_5Anaesthetist	0.0333
22	Pain_4Other	team_6Intensivist	0.0980
24	Pain_4Other	team_8Other	0.3259
25	Pain_4Other	Cardiac_consultation	0.7419
26	Pain_4Other	ANRQ_scoreRECORDED	0.7609
27	Pain_4Other	EPPSD_scoreRECORDED	0.9222
28	Pain_4Other	Age	0.9811
29	Pain_4Other	Weight	0.0400
31	Pain_4Other	BMI	0.0293
34	Pain_4Other	Live_baby_weight	0.0018
35	Pain_4Other	Babys_length_birth	0.4127
36	Pain_4Other	Babys_Head_circ_birth	0.0226
37	Pain_4Other	ANRQ_score	0.6304
38	Pain_4Other	EPPSD_score	0.3249
39	Pain_4Other	Gestational_age_admission	0.0066
40	Pain_4Other	Gravida	0.2123
41	Pain_4Other	Parity	0.2182
42	Pain_4Other	Gestational_age	0.0225
43	Pain_4Other	Apgar_1min	0.1293
44	Pain_4Other	Apgar_5min	0.1426
1	Cardiac_consultation	Revised_Groups	0.6959
2	Cardiac_consultation	Site	0.4421
3	Cardiac_consultation	Highrisk1	<.0001
4	Cardiac_consultation	Highrisk2	0.0990
5	Cardiac_consultation	Highrisk3	<.0001

Model number	*Outcome variable*	*Predictor variable*	*Global P value*
6	Cardiac_consultation	Highrisk4	<.0001
7	Cardiac_consultation	Highrisk6	0.0269
8	Cardiac_consultation	Highrisk7	<.0001
9	Cardiac_consultation	Highrisk5_sum	0.0007
10	Cardiac_consultation	Highrisk5_1Anaesthet	0.0063
11	Cardiac_consultation	Highrisk5_2Cardiolog	0.0015
12	Cardiac_consultation	Highrisk5_3Paediatri	0.0486
13	Cardiac_consultation	Highrisk9_1episiotom	0.8328
14	Cardiac_consultation	Highrisk9_2forceps	0.2877
15	Cardiac_consultation	Highrisk9_4No	0.8054
16	Cardiac_consultation	team_sum	0.0010
17	Cardiac_consultation	team_1Obstetrician	0.0043
18	Cardiac_consultation	team_2Midwife	.
19	Cardiac_consultation	team_3Cardiologist	.
20	Cardiac_consultation	team_4Physician	0.0166
21	Cardiac_consultation	team_5Anaesthetist	0.0010
22	Cardiac_consultation	team_6Intensivist	.
24	Cardiac_consultation	team_8Other	0.3405
25	Cardiac_consultation	Cardiac_consultation	1.0000
26	Cardiac_consultation	ANRQ_scoreRECORDED	0.4634
27	Cardiac_consultation	EPPSD_scoreRECORDED	0.0954
28	Cardiac_consultation	Age	0.1829
29	Cardiac_consultation	Weight	0.7380
31	Cardiac_consultation	BMI	0.7632
34	Cardiac_consultation	Live_baby_weight	0.0028
35	Cardiac_consultation	Babys_length_birth	0.0339
36	Cardiac_consultation	Babys_Head_circ_birth	0.1219
37	Cardiac_consultation	ANRQ_score	0.3482
38	Cardiac_consultation	EPPSD_score	0.5455
39	Cardiac_consultation	Gestational_age_admission	0.0027
40	Cardiac_consultation	Gravida	0.4077
41	Cardiac_consultation	Parity	0.2762
42	Cardiac_consultation	Gestational_age	0.0005
43	Cardiac_consultation	Apgar_1min	0.1541
44	Cardiac_consultation	Apgar_5min	0.0202

Model number	*Outcome variable*	*Predictor variable*	*Global P value*
1	Paediatric_neonatal_staff	Revised_Groups	0.8214
2	Paediatric_neonatal_staff	Site	0.2391
3	Paediatric_neonatal_staff	Highrisk1	<.0001
4	Paediatric_neonatal_staff	Highrisk2	0.7159
5	Paediatric_neonatal_staff	Highrisk3	0.0037
6	Paediatric_neonatal_staff	Highrisk4	<.0001
7	Paediatric_neonatal_staff	Highrisk6	<.0001
8	Paediatric_neonatal_staff	Highrisk7	0.0220
9	Paediatric_neonatal_staff	Highrisk5_sum	<.0001
10	Paediatric_neonatal_staff	Highrisk5_1Anaesthet	0.0016
11	Paediatric_neonatal_staff	Highrisk5_2Cardiolog	0.0184
12	Paediatric_neonatal_staff	Highrisk5_3Paediatri	<.0001
13	Paediatric_neonatal_staff	Highrisk9_1episiotom	0.7720
14	Paediatric_neonatal_staff	Highrisk9_2forceps	0.0073
15	Paediatric_neonatal_staff	Highrisk9_4No	0.3528
16	Paediatric_neonatal_staff	team_sum	<.0001
17	Paediatric_neonatal_staff	team_1Obstetrician	<.0001
18	Paediatric_neonatal_staff	team_2Midwife	0.4819
19	Paediatric_neonatal_staff	team_3Cardiologist	0.0363
20	Paediatric_neonatal_staff	team_4Physician	<.0001
21	Paediatric_neonatal_staff	team_5Anaesthetist	<.0001
22	Paediatric_neonatal_staff	team_6Intensivist	0.0095
24	Paediatric_neonatal_staff	team_8Other	0.5347
25	Paediatric_neonatal_staff	Cardiac_consultation	0.0015
26	Paediatric_neonatal_staff	ANRQ_scoreRECORDED	0.0441
27	Paediatric_neonatal_staff	EPPSD_scoreRECORDED	0.0977
28	Paediatric_neonatal_staff	Age	0.3521
29	Paediatric_neonatal_staff	Weight	0.3284
31	Paediatric_neonatal_staff	BMI	0.1220
34	Paediatric_neonatal_staff	Live_baby_weight	<.0001
35	Paediatric_neonatal_staff	Babys_length_birth	0.0021
36	Paediatric_neonatal_staff	Babys_Head_circ_birth	0.0007
37	Paediatric_neonatal_staff	ANRQ_score	0.4680
38	Paediatric_neonatal_staff	EPPSD_score	0.4744
39	Paediatric_neonatal_staff	Gestational_age_admission	0.0547

Model number	*Outcome variable*	*Predictor variable*	*Global P value*
40	Paediatric_neonatal_staff	Gravida	0.1933
41	Paediatric_neonatal_staff	Parity	0.0961
42	Paediatric_neonatal_staff	Gestational_age	0.0048
43	Paediatric_neonatal_staff	Apgar_1min	0.0016
44	Paediatric_neonatal_staff	Apgar_5min	0.0132

Table 3.Ordinal logistic regression results

Model number	*Outcome variable*	*Predictor variable*	*Global P value*
1	ANC1	Revised_Groups	0.0694
2	ANC1	Site	<.0001
3	ANC1	Highrisk1	0.9275
4	ANC1	Highrisk2	<.0001
5	ANC1	Highrisk3	0.0033
6	ANC1	Highrisk4	0.0132
7	ANC1	Highrisk6	0.7307
8	ANC1	Highrisk7	0.4124
9	ANC1	Highrisk5_sum	0.8503
10	ANC1	Highrisk5_1Anaesthet	0.9976
11	ANC1	Highrisk5_2Cardiolog	0.3295
12	ANC1	Highrisk5_3Paediatri	0.0708
13	ANC1	Highrisk9_1episiotom	0.2829
14	ANC1	Highrisk9_2forceps	0.9791
15	ANC1	Highrisk9_4No	0.4770
16	ANC1	team_sum	0.8828
17	ANC1	team_1Obstetrician	0.2424
18	ANC1	team_2Midwife	0.0520
19	ANC1	team_3Cardiologist	0.9372
20	ANC1	team_4Physician	0.1045
21	ANC1	team_5Anaesthetist	0.9205
22	ANC1	team_6Intensivist	0.6762
24	ANC1	team_8Other	0.8838
25	ANC1	Cardiac_consultation	0.0950
26	ANC1	ANRQ_scoreRECORDED	<.0001

Model number	*Outcome variable*	*Predictor variable*	*Global P value*
27	ANC1	EPPSD_scoreRECORDED	<.0001
28	ANC1	Age	0.6721
29	ANC1	Weight	0.4811
31	ANC1	BMI	0.6502
34	ANC1	Live_baby_weight	0.9540
35	ANC1	Babys_length_birth	0.6732
36	ANC1	Babys_Head_circ_birth	0.6426
37	ANC1	ANRQ_score	0.7858
38	ANC1	EPPSD_score	0.0389
39	ANC1	Gestational_age_admission	0.0316
40	ANC1	Gravida	0.8872
41	ANC1	Parity	0.6398
42	ANC1	Gestational_age	0.0457
43	ANC1	Apgar_1min	0.5391
44	ANC1	Apgar_5min	0.5334
1	ANC2	Revised_Groups	0.4112
2	ANC2	Site	0.0063
3	ANC2	Highrisk1	0.0021
4	ANC2	Highrisk2	0.0048
5	ANC2	Highrisk3	0.2270
6	ANC2	Highrisk4	0.2374
7	ANC2	Highrisk6	0.5490
8	ANC2	Highrisk7	0.5268
9	ANC2	Highrisk5_sum	0.0813
10	ANC2	Highrisk5_1Anaesthet	0.8809
11	ANC2	Highrisk5_2Cardiolog	0.1693
12	ANC2	Highrisk5_3Paediatri	0.4676
13	ANC2	Highrisk9_1episiotom	0.9552
14	ANC2	Highrisk9_2forceps	0.6666
15	ANC2	Highrisk9_4No	0.5635
16	ANC2	team_sum	0.2914
17	ANC2	team_1Obstetrician	0.9012
18	ANC2	team_2Midwife	0.2963
19	ANC2	team_3Cardiologist	0.9218
20	ANC2	team_4Physician	0.1329

Model number	*Outcome variable*	*Predictor variable*	*Global P value*
21	ANC2	team_5Anaesthetist	0.0731
22	ANC2	team_6Intensivist	0.5904
24	ANC2	team_8Other	0.5521
25	ANC2	Cardiac_consultation	0.0366
26	ANC2	ANRQ_scoreRECORDED	0.9492
27	ANC2	EPPSD_scoreRECORDED	0.4947
28	ANC2	Age	0.9075
29	ANC2	Weight	0.9490
31	ANC2	BMI	0.9280
34	ANC2	Live_baby_weight	0.2280
35	ANC2	Babys_length_birth	0.0159
36	ANC2	Babys_Head_circ_birth	0.9225
37	ANC2	ANRQ_score	0.0261
38	ANC2	EPPSD_score	0.2693
39	ANC2	Gestational_age_admission	0.1497
40	ANC2	Gravida	0.8123
41	ANC2	Parity	0.5409
42	ANC2	Gestational_age	0.1768
43	ANC2	Apgar_1min	0.8066
44	ANC2	Apgar_5min	0.6593
1	ANC3	Revised_Groups	0.4757
2	ANC3	Site	0.6850
3	ANC3	Highrisk1	0.0023
4	ANC3	Highrisk2	0.7838
5	ANC3	Highrisk3	0.0278
6	ANC3	Highrisk4	0.0654
7	ANC3	Highrisk6	0.0283
8	ANC3	Highrisk7	0.4304
9	ANC3	Highrisk5_sum	0.2212
10	ANC3	Highrisk5_1Anaesthet	0.0468
11	ANC3	Highrisk5_2Cardiolog	0.0926
12	ANC3	Highrisk5_3Paediatri	0.7238
13	ANC3	Highrisk9_1episiotom	0.4438
14	ANC3	Highrisk9_2forceps	0.7231
15	ANC3	Highrisk9_4No	0.9794

Model number	*Outcome variable*	*Predictor variable*	*Global P value*
16	ANC3	team_sum	0.4115
17	ANC3	team_1Obstetrician	0.8469
18	ANC3	team_2Midwife	0.4927
19	ANC3	team_3Cardiologist	0.3471
20	ANC3	team_4Physician	0.2058
21	ANC3	team_5Anaesthetist	0.0361
22	ANC3	team_6Intensivist	0.7128
24	ANC3	team_8Other	0.1267
25	ANC3	Cardiac_consultation	0.2401
26	ANC3	ANRQ_scoreRECORDED	0.6266
27	ANC3	EPPSD_scoreRECORDED	0.5476
28	ANC3	Age	0.7511
29	ANC3	Weight	0.6275
31	ANC3	BMI	0.4532
34	ANC3	Live_baby_weight	0.9453
35	ANC3	Babys_length_birth	0.3968
36	ANC3	Babys_Head_circ_birth	0.7454
37	ANC3	ANRQ_score	0.7666
38	ANC3	EPPSD_score	0.5026
39	ANC3	Gestational_age_admission	0.1605
40	ANC3	Gravida	0.6186
41	ANC3	Parity	0.2049
42	ANC3	Gestational_age	0.0653
43	ANC3	Apgar_1min	0.6651
44	ANC3	Apgar_5min	0.7050
1	FetalRisk1_sum	Revised_Groups	0.9762
2	FetalRisk1_sum	Site	0.2047
3	FetalRisk1_sum	Highrisk1	<.0001
4	FetalRisk1_sum	Highrisk2	0.0272
5	FetalRisk1_sum	Highrisk3	0.0001
6	FetalRisk1_sum	Highrisk4	<.0001
7	FetalRisk1_sum	Highrisk6	0.0038
8	FetalRisk1_sum	Highrisk7	<.0001
9	FetalRisk1_sum	Highrisk5_sum	0.0052
10	FetalRisk1_sum	Highrisk5_1Anaesthet	0.0046

Model number	*Outcome variable*	*Predictor variable*	*Global P value*
11	FetalRisk1_sum	Highrisk5_2Cardiolog	0.2213
12	FetalRisk1_sum	Highrisk5_3Paediatri	0.0080
13	FetalRisk1_sum	Highrisk9_1episiotom	0.3344
14	FetalRisk1_sum	Highrisk9_2forceps	0.6538
15	FetalRisk1_sum	Highrisk9_4No	0.6343
16	FetalRisk1_sum	team_sum	0.0010
17	FetalRisk1_sum	team_1Obstetrician	0.0227
18	FetalRisk1_sum	team_2Midwife	0.6411
19	FetalRisk1_sum	team_3Cardiologist	0.0255
20	FetalRisk1_sum	team_4Physician	0.1047
21	FetalRisk1_sum	team_5Anaesthetist	0.0148
22	FetalRisk1_sum	team_6Intensivist	0.0590
24	FetalRisk1_sum	team_8Other	0.9661
25	FetalRisk1_sum	Cardiac_consultation	0.0003
26	FetalRisk1_sum	ANRQ_scoreRECORDED	0.4655
27	FetalRisk1_sum	EPPSD_scoreRECORDED	0.7011
28	FetalRisk1_sum	Age	0.9986
29	FetalRisk1_sum	Weight	0.0363
31	FetalRisk1_sum	BMI	0.0118
34	FetalRisk1_sum	Live_baby_weight	0.0094
35	FetalRisk1_sum	Babys_length_birth	0.3799
36	FetalRisk1_sum	Babys_Head_circ_birth	0.2796
99937	FetalRisk1_sum	ANRQ_score	0.7940
38	FetalRisk1_sum	EPPSD_score	0.1216
39	FetalRisk1_sum	Gestational_age_admission	0.1119
40	FetalRisk1_sum	Gravida	0.5456
941	FetalRisk1_sum	Parity	0.9936
42	FetalRisk1_sum	Gestational_age	0.0132
43	FetalRisk1_sum	Apgar_1min	0.1853
44	FetalRisk1_sum	Apgar_5min	0.0592
1	compromise_sum	Revised_Groups	0.7505
2	compromise_sum	Site	<.0001
3	compromise_sum	Highrisk1	0.9332
4	compromise_sum	Highrisk2	0.2695
5	compromise_sum	Highrisk3	0.0380

Model number	*Outcome variable*	*Predictor variable*	*Global P value*
6	compromise_sum	Highrisk4	<.0001
7	compromise_sum	Highrisk6	0.7662
8	compromise_sum	Highrisk7	0.9547
9	compromise_sum	Highrisk5_sum	0.6450
10	compromise_sum	Highrisk5_1Anaesthet	0.4216
11	compromise_sum	Highrisk5_2Cardiolog	0.5342
12	compromise_sum	Highrisk5_3Paediatri	0.5314
13	compromise_sum	Highrisk9_1episiotom	0.0007
14	compromise_sum	Highrisk9_2forceps	0.9911
15	compromise_sum	Highrisk9_4No	0.3535
16	compromise_sum	team_sum	0.4647
17	compromise_sum	team_1Obstetrician	0.3437
18	compromise_sum	team_2Midwife	.
19	compromise_sum	team_3Cardiologist	0.7408
20	compromise_sum	team_4Physician	0.9869
21	compromise_sum	team_5Anaesthetist	0.6690
22	compromise_sum	team_6Intensivist	0.6964
24	compromise_sum	team_8Other	.
25	compromise_sum	Cardiac_consultation	0.8664
26	compromise_sum	ANRQ_scoreRECORDED	0.8393
27	compromise_sum	EPPSD_scoreRECORDED	0.1763
28	compromise_sum	Age	0.4599
29	compromise_sum	Weight	0.3818
31	compromise_sum	BMI	0.3277
34	compromise_sum	Live_baby_weight	0.6619
35	compromise_sum	Babys_length_birth	0.6864
36	compromise_sum	Babys_Head_circ_birth	0.2725
37	compromise_sum	ANRQ_score	0.4236
38	compromise_sum	EPPSD_score	0.0284
39	compromise_sum	Gestational_age_admission	0.2867
40	compromise_sum	Gravida	0.4873
41	compromise_sum	Parity	0.8665
42	compromise_sum	Gestational_age	0.2858
43	compromise_sum	Apgar_1min	0.7354
44	compromise_sum	Apgar_5min	0.1731

Model number	*Outcome variable*	*Predictor variable*	*Global P value*
1	Pain_sum	Revised_Groups	0.0054
2	Pain_sum	Site	0.4158
3	Pain_sum	Highrisk1	0.0018
4	Pain_sum	Highrisk2	0.6872
5	Pain_sum	Highrisk3	0.0020
6	Pain_sum	Highrisk4	0.0895
7	Pain_sum	Highrisk6	0.0113
8	Pain_sum	Highrisk7	0.7848
9	Pain_sum	Highrisk5_sum	0.0693
10	Pain_sum	Highrisk5_1Anaesthet	0.5523
11	Pain_sum	Highrisk5_2Cardiolog	0.2887
12	Pain_sum	Highrisk5_3Paediatri	0.0143
13	Pain_sum	Highrisk9_1episiotom	0.7469
14	Pain_sum	Highrisk9_2forceps	0.7488
15	Pain_sum	Highrisk9_4No	0.0740
16	Pain_sum	team_sum	<.0001
17	Pain_sum	team_1Obstetrician	<.0001
18	Pain_sum	team_2Midwife	0.1778
19	Pain_sum	team_3Cardiologist	0.0336
20	Pain_sum	team_4Physician	0.0318
21	Pain_sum	team_5Anaesthetist	<.0001
22	Pain_sum	team_6Intensivist	0.0128
24	Pain_sum	team_8Other	0.3237
25	Pain_sum	Cardiac_consultation	0.0586
26	Pain_sum	ANRQ_scoreRECORDED	0.0559
27	Pain_sum	EPPSD_scoreRECORDED	0.5899
28	Pain_sum	Age	0.4796
29	Pain_sum	Weight	0.3543
31	Pain_sum	BMI	0.5213
34	Pain_sum	Live_baby_weight	<.0001
35	Pain_sum	Babys_length_birth	0.0232
36	Pain_sum	Babys_Head_circ_birth	0.0046
37	Pain_sum	ANRQ_score	0.7901
38	Pain_sum	EPPSD_score	0.2746
39	Pain_sum	Gestational_age_admission	0.0328

Model number	*Outcome variable*	*Predictor variable*	*Global P value*
40	Pain_sum	Gravida	0.2303
41	Pain_sum	Parity	0.2780
42	Pain_sum	Gestational_age	0.0014
43	Pain_sum	Apgar_1min	0.5088
44	Pain_sum	Apgar_5min	0.5174
