# Supplementary material for: Adherence to clinical practice guidelines for South Australian pregnant women with cardiac conditions between 2003 and 2013
Source: PLoS One. 2020 Mar 17;15(3):e0230459. doi: 10.1371/journal.pone.0230459 (PMC7077829; doi:10.1371/journal.pone.0230459)
Supplement: S5 Table — (PDF) [file pone.0230459.s005.pdf]

All data: Frequency tables of cardiac variable

The FREQ Procedure

Previous_pregnancies_with_cardia	Frequency	Percent	Cumulative Frequency	Cumulative Percent
No	122	45.86	122	45.86
Yes	144	54.14	266	100.00
Frequency Missing = 5				

cardiac event in Pregnancy				
Cardiac	Frequency	Percent	Cumulative Frequency	Cumulative Percent
HF	40	17.62	40	17.62
Arrhyth	74	32.60	114	50.22
IHD	20	8.81	134	59.03
CArrest	11	4.85	145	63.88
V& CHD	55	24.23	200	88.11
SBE	2	0.88	202	88.99
CVA	1	0.44	203	89.43
RHD	22	9.69	225	99.12
Cardiac PE	1	0.44	226	99.56
Aortic Dissect	1	0.44	227	100.00
Frequency Missing = 44				

AC data: Frequency tables of cardiac variable

The FREQ Procedure

Previous_pregnancies_with_cardia	Frequency	Percent	Cumulative Frequency	Cumulative Percent
No	82	66.67	82	66.67
Yes	41	33.33	123	100.00
Frequency Missing = 5				

Cardiac event in Pregnancy				
Cardiac	Frequency	Percent	Cumulative Frequency	Cumulative Percent
HF	28	25.69	28	25.69
Arrhythmia	49	44.95	77	70.64
IHD	18	16.51	95	87.16
CArrest	9	8.26	104	95.41
V&/CHD	2	1.83	106	97.25
SBE	1	0.92	107	98.17
CVA	1	0.92	108	99.08
Cardiac PE	1	0.92	109	100.00
Frequency Missing = 19				

PEC data: Frequency tables of cardiac variable

The FREQ Procedure

Previous_pregnancies_with_cardiac				
Previous_pregnancies_with_cardia	*Frequency*	*Percent*	*Cumulative Frequency*	*Cumulative Percent*
No	40	27.97	40	27.97
Yes	103	72.03	143	100.00

cardiac	*Frequency*	*Percent*	*Cumulative Frequency*	*Cumulative Percent*
HF	12	10.17	12	10.17
ArrHyt hmia	25	21.19	37	31.36
IHD	2	1.69	39	33.05
Card Arrest	2	1.69	41	34.75
VHD/C HD	53	44.92	94	79.66
SBE	1	0.85	95	80.51
RHD	22	18.64	117	99.15
Aortic Dissect	1	0.85	118	100.00
Frequency Missing = 25				

Cardiac Characteristics	**Total -Frequency (%)**	**PEC - Frequency (%)**	**AC - Frequency (%)**
Cardiac event (prev preg)	144 (54)	103 (72)	*41 (33)
Cardiac event (current preg)			
1 Heart failure HF	40 (18)	12 (10)	28 (26)
2 Arrhythmias	74 (33)	25 (21)	49 (45)
3 IHD	20 (9)	2 (2)	18 (17)
4 Cardiac Arrest	11 (5)	2 (2)	9 (8)
5 VHD / CHD	55 (24)	53 (45)	2 (2)
6 SBE	2 (1)	1 (1)	1 (1)
7 CVA	1 (0)	0	1 (1)
8 RHD	22 (10)	22 (19)	0
9 Cardiac PE	1 (0)	0	1 (1)
10 Aortic Dissection	1 (0)	1 (1)	0

Arrhythmias included: SVT/ VT/ Sinus bradycardia and complete heart block (CHB).
IHD: Ischemic Heart Disease.
VHD/ CHD: included valvular heart disease and congenital heart disease.
SBE: Subacute Bacterial Endocarditis. CVA: Cerebrovascular accident.
RHD: rheumatic heart disease.
Cardiac PE: Cardiac pulmonary embolus. Type B Aortic dissection.
