# Supplementary material for: Adherence to clinical practice guidelines for South Australian pregnant women with cardiac conditions between 2003 and 2013
Source: PLoS One. 2020 Mar 17;15(3):e0230459. doi: 10.1371/journal.pone.0230459 (PMC7077829; doi:10.1371/journal.pone.0230459)
Supplement: S1 File — (PDF) [file pone.0230459.s006.pdf]

Perinatal Audit

Demographics

1. Study code
2. Site

1=Tertiary hospital, 2=Quaternary hospital, 3=Stand-alone Maternity Unit
Choose one answer

- 1
- 2
- 3

3. Age
4. Country of birth
5. Geographical postcode

Choose one answer

1. Rural
2. Remote
3. Metropolitan
4. Unknown

6. Maternal race classification

Choose one answer

1. Caucasian
2. Aboriginal/Torres Strait Islander
3. Asian
4. Other:

7. Previous pregnancies with cardiac history?

Choose one answer

1. Yes
2. No previous pregnancies
3. Has had previous pregnancies but not with cardiac history

Details of previous pregnancy with cardiac history insert text
Follow-up for response of 'Yes'...

8. Weight

9. Height in cm

10. Body mass index

11. Current Social Status

Choose one answer

1. Married
2. De facto/ living with partner
3. Divorced/Separated
4. Widowed
5. Never married/Single

Cardiac pathology identified

12. Entry point

Group A: Pre-existing cardiac condition, Group B: Newly acquired cardiac condition during course of pregnancy and so care may not be reflective of perinatal guidelines.
Choose one or more answers

1. Congenital (A)
2. Acquired A (acute event first trimester) (B)
3. Acquired B (at delivery) (B)

4. Acquired C (post-partum) (B)
5. Cyanotic (A)
6. Acyanotic (A)
7. Other:

13. Cause of cardiac disease

Choose one or more answers

1. Rheumatic heart disease (A)
2. Congenital heart disease (A)
3. Arrhythmias (B) (A)
4. Ischaemic Heart Disease (IHD) (B) (A)
5. Heart Failure (B) (A)
6. Cardiac arrest (B)
7. Conduction disorders (B)
8. PE (B)
9. Hypertension with severe cardiac condition (B)
10. Interventional coronary procedure (B)
11. Other (B) (A)

14. New York Heart Association (NYHA) Functional Classification on admission or Documented.

Applicable to category A conditions only Choose one or more answers

1. I
2. II
3. III
4. IV
5. Not applicable
6. Unknown

Pre-conception care

15. Documented discussion before conception, which is applicable to group A only. For all others leave as not applicable

Choose one answer

1. Yes
2. No
3. Not applicable
4. Unknown.

16. General physical assessment

Tick all which apply, Choose one or more answers

1. Weight
2. Height
3. BMI
4. Oral health
5. Respiratory examination (auscultation)
6. Breast Examination
7. Abdominal Examination
8. Vaginal examination (speculum/vaginal exam + swabs if clinically indicated)
9. Urinalysis
10. Psychosocial assessment
11. Not applicable
12. Unknown

17. Medication assessment:
Insert text

18. Cardiac assessment
Choose one or more answers

1. Blood pressure
2. Pulse
3. 12 lead ECG
4. Cardiac biomarkers
5. Cardiac auscultation
6. Echocardiogram
7. Not applicable
8. Unknown.

19. Cardiac assessment comments.

Insert text

20. Healthcare professionals who performed assessment
Choose one or more answers

1. Physician/medical officer
2. Midwife
3. Cardiac specialist nurse
4. Obstetrician
5. Not applicable
6. Unknown
7. Other:

21. Comments on healthcare professional who completed assessment
Insert text

22. Hypertension management
Choose one answer

1. Not identified
2. Identified and management optimised/reviewed
3. Identified and management NOT optimised/reviewed
4. Not applicable
5. Unknown

23. Systolic blood pressure value:

24. Diastolic blood pressure value:

25. Arrhythmia management
Choose one answer

1. Not identified
2. Identified and management optimised/reviewed

3. Identified and management NOT optimised/reviewed
4. Not applicable
5. Unknown

26. Lifestyle factors identified
Choose one or more answers
1. Smoking
2. Exercise tolerance
3. Documented discussion with the woman, family and cardiologist re lifestyle factors
4. Psychosocial evaluation (identify tool used)
5. Not applicable

ANRQ score
Follow-up for response of 'Psychosocial evaluation (identify tool used) '...insert value and text
EPPSD score
Follow-up for response of 'Psychosocial evaluation (identify tool used) '...insert value and text

Preconception cardiac education

27. Documentation of cardiac education
Choose one answer
1. Yes
2. No
3. Not applicable

28. Patient informed regarding the potential expected outcomes
Choose one or more answers
1. spontaneous miscarriage
2. live birth
3. death
4. antenatal care
5. anaesthesia
6. proposed care plan for pregnancy
7. medications in the Peripartum management
8. genetic counselling
9. Not applicable
10. Unknown

Antenatal management

29. Antenatal management offered
Choose one or more answers
1. Routine visit within 10-12 weeks
2. Early ultrasound to establish estimated date of confirmation (EDC)
3. Documented multidisciplinary team management that includes: obstetrician,
4. cardiologist, obstetric physician, anaesthetist, intensivist

5. If applicable -fortnightly antenatal visits in high risk medical clinic dependant on
6. severity of disease.
7. Not applicable high risk antenatal visits
8. Avoid anaemia+ regular Hb checks.
9. Iron supplements + prenatal vitamins and dietary counselling
10. Anaesthetic review before 28 weeks
11. Unknown

Clinical assessment

30. Maternal age

31. Gestational age

32. Gravida

33. Parity

34. NYHA functional classification

Choose one answer

1. Documented
2. Not documented
3. Unknown
4. Not applicable

35. Comorbid conditions

Choose one or more answers

1. Previous cardiac events
2. Previous cardiac surgery
3. Previous cardiac interventions
4. Past/current cardiac lesions
5. Past/current cyanosis (Sa)2 <90%)
6. Medication history
7. Use of cigarettes and/or alcohol
8. Not applicable
9. Unknown

36. Cardiac events additional information

Insert text

37. Cardiac surgery additional information

Insert text

38. Cardiac interventions additional information

Insert text

39. Past/current cardiac lesions additional information

Insert text

40. Past/current cyanosis additional information

Insert text

41. Medication history additional information

Insert text

42. Use of cigarettes and/or alcohol additional information

Insert text

Clinical assessment: Investigations

43. 12 lead ECG findings

44. Echocardiogram findings

45. Fetal wellbeing/risks

Choose one or more answers

1. Regular (fortnightly) ultrasound assessment of fetal wellbeing
2. Growth, umbilical artery Doppler, AFI
3. Cardiotocography (CTG) in third trimester
4. Unknown
5. Not applicable

46. Fetal risks

Choose one or more answers

1. Fetal echocardiogram at 20 weeks of gestation if woman on warfarin or identified cardiac fetal anomaly
2. Amniocentesis with antibiotic prophylaxis
3. Unknown

Planned care

47. Echocardiogram assessment

Choose one answer

1. Yes
2. No
3. Not applicable
4. Unknown

48. Ongoing NYHA functional classification to assist with hospital choice

Choose one answer

1. Yes
2. No
3. Not applicable
4. Unknown

49. Planned Care: Level of Hospital Care comments

50. Planned delivery mode

Choose one answer

1. Vaginal birth
2. Caesarean section for obstetric emergency only
3. Caesarean section for other
4. Not applicable
5. Unknown

51. Planned delivery mode comments

Insert

High Risk management in labour

52. Identified as high risk

Choose one answer

1. Yes
2. No
3. Not applicable
4. Unknown

53. Labour and birth occurred in a hospital with adult intensive care or cardiac unit

Choose one answer

1. Yes
2. No
3. Not applicable

54. Documented plan of care in case-notes

Choose one answer

1. Yes
2. No
3. Unknown
4. Not applicable

55. Timing and labour of birth

Choose one answer

1. Multidisciplinary team discussion
2. Cardiac state well stabilised
3. At short notice in event of cardiac function and reserve altered
4. Unknown
5. Not applicable

56. During labour, the following multidisciplinary team members informed of impending birth and documented

Choose one or more answers

1. Cardiologist or physician
2. Anaesthetist
3. Paediatrician
4. Not applicable
5. Unknown

57. Appropriate antibiotic prophylaxis considered

Choose one answer

1. Yes
2. No
3. Not applicable
4. Unknown

58. Planned cardiac drugs required in consultation with multidisciplinary team

Choose one answer

1. Yes
2. No
3. Not applicable
4. Unknown

59. High risk management in labour comments

Insert text.

60. Was the second stage of birth shortened to avoid excessive maternal expulsive effort?
Choose one or more answers

1. No
2. Yes with episiotomy
3. Yes with forceps delivery
4. Yes with no local anaesthetics with adrenaline being used
5. Not applicable
6. Unknown

61. Minimisation of cardiac compromise
Choose one or more answers
Consultation re avoiding the use of stirrups

- Assessment + prompt reporting of Tachycardia >130bpm
- Assessment + prompt reporting of Bradycardia < 40bpm
- Assessment + prompt reporting of respiratory rate >24 or < 5 bpm
- Assessment+ prompt reporting rapidly changing pulse or respiration rate (even if
- within threshold)
- Not applicable
- Unknown

62. Birth comments
Insert text

63. Management in labour: Adult resuscitation equipment available?
Choose one answer

1. Yes
2. No

64. Management in labour: Ability to perform perimortem Caesarean section within 5 minutes of cardiac arrest
Choose one answer

1. Yes
2. No

65. Multidisciplinary team included in management of labour
Choose one or more answers

1. Obstetrician
2. Midwife
3. Cardiologist
4. Physician/ medical officer
5. Anaesthetist
6. Intensivist
7. Other allied health professional
8. Other:

66. Management in labour comments
Insert text

67. Thromboembolism prophylaxis
Choose one or more answers

1. Use of anti- embolic stockings

2. Subcutaneous LMWH or IV Unfractionated heparin
3. Not applicable
4. Unknown

68. Pain Management and Anaesthesia
tick all which apply
Choose one or more answers

1. Anaesthetic consultation regarding appropriate pain management undertaken
2. Epidural considered
3. Combined spinal-epidural anaesthesia to decrease preload and afterload
4. Not applicable
5. Other:

69. Pain management and anaesthesia comments.

Insert text

Preterm labour imminent

70. Cardiac consultation with cardiologist and physician advice sought
Choose one answer

1. Yes
2. No
3. Not applicable
4. Unknown

71. Paediatric and neonatal staff available.
Choose one answer

1. Yes
2. No
3. Unknown

Perinatal outcome of baby

72. Outcome of birth

73. Gestational age

74. Live baby weight grams

75. Baby's length at birth

76. Baby's Head circumference at birth

77. Apgar score at 1 min Bounds: 0 – 10 [inclusive]

78. Apgar at 5 min Bounds: 0 – 10 [inclusive]

79. Cardiac follow-up included in obstetric follow-up care

Checklist or nursing or medical documentation

Text Comments
