# Supplementary material for: Adherence to clinical practice guidelines for South Australian pregnant women with cardiac conditions between 2003 and 2013
Source: PLoS One. 2020 Mar 17;15(3):e0230459. doi: 10.1371/journal.pone.0230459 (PMC7077829; doi:10.1371/journal.pone.0230459)
Supplement: Attachment — Submitted filename: Tabulated Response to Plos One Reviewer feedback 05022020.docx [file pone.0230459.s007.docx]

| Reviewer #1 feedback comments | Authors comment | Response | Page Number on Clean copy |
| --- | --- | --- | --- |
| 1. Is the manuscript technically sound, and do the data support the conclusions? Partly |  |  |  |
| 2. Have the authors made all data underlying the findings in their manuscript fully available? Reviewer #1 response: No | The authors acknowledge the reviewers' response regarding providing all data. However, all the relevant data was made available in supporting information. Ethical requirements preclude sharing identifiable individual data, particularly from a retrospective medical review. |  |  |
| Reviewer #1:  The manuscript evaluated and discussed a relevant theme; however, it is not clear. Introduction shows reference 9 and 10 as the guidelines providing recommendations, but reference 9 is a study about following the previous guidelines. | We acknowledge the reviewer’s comment relation to reference 9 and 10 that highlighted previous guidelines. It is important to note that cardiac disease in pregnancy is under-researched in Australia, as reported by the Australian Maternity Outcome Surveillance System (AMOSS).  <https://www.amoss.com.au/content/cardiac-disease-pregnancy>  The researcher resourced the most recent Australian literature for national guidelines and associated research. The perinatal guidelines used in this study were the only available perinatal guidelines pertinent to cardiac conditions in pregnancy on the National Health and Medical Research (NHMRC) website at the commencement of the PhD project.  More importantly, no evaluation of the statewide perinatal guidelines has occurred since the implementation in 2010, and therefore this current project is ground-breaking. While regular revisions of South Australian perinatal practice guidelines (SAPPG) occur, this is the first evaluation on the adherence to the guidelines across the public health sector. As pointed out in the manuscript ‘Measuring clinical practice benchmarked against recommendations in the guidelines, and best practice is a crucial step to address the evidence-practice gaps [18].’ The SAAPG guidelines pertinent to cardiac disease in pregnancy were the only available guidelines at the time and has been made more explicit in the manuscript. | The epidemiology of cardiac disease in pregnancy varies internationally with 0.2-4% estimates; however, it remains under-researched in Australia as reported by Australian Maternity Outcome Surveillance System (AMOSS) [1, 2].  Australian national guidelines for the care of healthy pregnant women published online in 2018 provide recommendations that support high quality, safe prenatal care in all settings; however, there were no specific guidelines for women with cardiac conditions during pregnancy [12, 13]. Recent research that examined women’s perception of prenatal care, provider adherence to antenatal care guidelines, the risk of pregnancy complications, and guidelines associated with cardiovascular risk factors reported suboptimal adherence [12, 14-16]. Given the increased risk of cardiovascular complications during pregnancy, with consequences for both mother and foetus, we need to identify the gap between existing evidence and clinical practice.  The South Australian Perinatal Practice Guidelines (SAPPGs) implemented in 2010, represented Australian practice guidelines at the time and included those specific to cardiac disease in pregnancy [17]. While regular revisions occur, this is the first evaluation on the uptake of the statewide guidelines across the public health sector. Measuring clinical practice against the best practice is a crucial step to address the evidence-practice gaps [18]. | Page 4,  Lines 75-77.  Page 5  Lines 101 -114. |
| Objective number 2 is described as "identify predictors of the quality of care for these women" but in fact, it evaluated guideline adherence. If it means quality of care must be justified. | We thank the reviewer for the comment regarding objective 2. The objectives have been corrected to reflect the objectives of the research study in both manuscript and abstract. | This study aimed to evaluate three public hospitals’ adherence to the guidelines, with the following three objectives:   1. Evaluate the adherence to the perinatal guidelines for women with pre-existent and newly acquired heart conditions during pregnancy. 2. Identify predictors of adherence for these women. 3. Make comparisons across the three hospitals examined. | Page 5  Lines 114-120. |
| The hospital codes are reported in inclusion criteria. It would be clearer if described cardiac conditions. Exclusion criteria that are shown are unnecessary. | We thank the reviewer for the comment regarding describing the cardiac conditions. The authors were constrained by author guidelines and compliance with Strobe checklist for observational studies. The inclusion criteria have been rephrased to describe better participants of the study and the hospital codes removed. The exclusion criteria remained to adhere to the Strobe checklist.  In additional the perinatal audit tool is available in the supporting information S5 which identifies the spectrum of cardiac conditions seen in both groups.  The focus of this paper was to report on adherence to guidelines given recent publications. The subsequent manuscript will provide a more comprehensive overview of the cardiac conditions, while reporting on maternal and foetal clinical outcomes. | The hospital codes removed and replaced with a description of entry points.  The participants in the study were women with ‘pre-existent’ or ‘newly acquired’ heart disease during pregnancy. The ‘pre-existing’ group included women with both structural and electrical cardiac conditions before conception. Structural cardiac conditions included cyanotic and acyanotic congenital heart disease, a previous history of hypertensive, valvular and cardiomyopathic heart disease as well as those women with ischemic heart disease. Electrical cardiac conditions included genetically inherited channelopathies as well as the full spectrum of acquired bradyarrhythmias and tachyarrhythmias. Women with symptomatic benign ectopic beats were excluded from this group. Similarly, women in whom undiagnosed congenital cardiac conditions were unmasked during pregnancy were included in the ‘newly acquired group’.  The ‘newly acquired’ group included women with pregnancy-associated cardiac events. This includes any new symptomatic sustained arrhythmia, the unmaking of underlying prior cardiac conditions previously unrecognised, and new-onset cardiac disease such as acute coronary syndrome, cardiomyopathy with or without decompensated heart failure, and infective endocarditis during pregnancy or up to 12 months postpartum. These inclusion criteria were similar to those used in the United Kingdom survey of obstetric deaths [19].  The International Statistical Classification of Diseases and Related Health Problems, Tenth Revision, Australian Modification (ICD-10-AM), identifies conditions related to or aggravated by pregnancy, childbirth or the puerperium (maternal or obstetric causes). The codes for this project were 099, 090.3, 089, 088, and 151, 142 and 125.5. | Page 6-7  Lines 138- 158 |
| Hospital 1, 2 and 3 are described out of order and reading becomes confused. | We thank the reviewer for the comment regarding the practice settings and therefore have repositioned below Study Design and Setting to improve the logical flow. | Setting  The three metropolitan hospitals in this study represent the complete SA health (Government funded) metropolitan public hospital inpatient obstetric service, as described in the guidelines [17]. Hospital One provided intermediate care (level five) that encompassed tertiary services, maternal intensive care and specialised neonatal care services excluding babies less than 32 weeks. The quaternary centre, hospital Two provided (level six) care that included maternal cardiac, cardiothoracic surgical and intensive care and specialised neonatal intensive care services. Hospital Three provided neonatal intensive care services but no maternal cardiac or intensive care services. | Page 6  Lines 127 -135. |
| Scoring system should be an attached file to improve comprehension of the method. | We thank the reviewer for the comment regarding the scoring system. Although additional information was in the supporting information however, this was not made explicit in the manuscript. A reference has been made to the Supplementary information S1 Revised. The table provides a comprehensive view of the Auditmaker Guideline Variables, SPSS/ SAS coding for statistical analysis, the scoring for adherence to the statewide perinatal guidelines and the data collected.  The perinatal Guideline audit tool (S5) has been provided as well in the supplementary audit tool | A scoring system was devised that measured adherence to available protocols and guidelines in this study [20,21]. The forty guidelines adherence variables used in this study [S1 Table) were equally weighted, giving a maximum score of forty to measure adherence. Positive documentation of the guidelines, regardless of the entry point, achieved a score.  . | Page 7,  Lines 167-171. |
| How did you choose the number 35 to classify as minimum acceptable score for 'pre-existent' cardiac condition and 17 for 'newly acquired'? | We thank the reviewer for the comment regarding the process of determining the minimum acceptable score for both cardiac groups. The text has been repositioned to the discussion section to reflect the research process and logical flow. See relevant discussion section with changes. | A minimum score of acceptable guideline adherence was determined after the comparison of the two groups mean, and median adherence scores and expert review of selected cases identifying minimum expected care [S1]. From this analysis, a score greater than 35 (for ‘pre-existent’) and 17 (for ‘newly acquired’) cardiac conditions were deemed acceptable guideline adherence.  Given the foreknowledge of the women’s cardiac state, the minimum guideline adherence variables for the ‘pre-existent group’ would include preconception education, comprehensive antenatal assessment including a prepregnancy cardiac functional assessment using New York Heart Association (NYHA) classification and multidisciplinary team collaboration [14].  Conversely, in the ‘newly acquired’ group with a pregnancy-induced cardiac event, adherence to the guidelines will be contingent upon the timing of the cardiac event during the peripartum period. Furthermore, it is reasonable not to expect 100% concordance with the guidelines. The expected minimum guideline adherence variables would include adult cardiologist or physician management soon after the cardiac event and the multidisciplinary team involvement for the rest of the peripartum care. | Page 22-23, Lines 367-382. |
| Sample was extracted from ALL medical records that met the inclusion criteria. | We thank the reviewer for the comment regarding the sample and have adjusted the text to reflect more accurately that all available medical records. | The sample was data abstracted from all the available medical records that met the inclusion criteria within the timeframe between 2003 and 2013. | Page 10 Line 219-220. |
| You observed significative differences between pre-existent and newly acquired group but all predictors are described for all subjects. | We thank the reviewer for comments regarding observed significative differences between pre-existent and newly acquired group, but all predictors are described for all subjects. Only the significant were included in table 2, as highlighted on the table footnote and discussed in the manuscript. A more comprehensive list of the predictors is available in the supporting information S3 –S4.  Table 3 addresses the research objectives 2 and 3 makes explicit the inclusion of priori predictors for the adherent guideline variable. | Those predictors identified as not significant (Global p >0.05) were maternal age, ethnicity, social status, country of birth, gravida, gestational age and specific cardiac causes such as RHD, arrhythmias, ischaemia, failure, and cardiac arrest, and therefore have been excluded. | Page 13 lines 291-294  Table 2 page 15. Supporting information S3-S4  Table 3 |
| Table 3 is the most important in the manuscript but is not understandable, too much data | We acknowledge the reviewer’s comment concerning Table 3. The presentation of results is formatted to improve understanding with the manuscript revised. The headings revised and included where tables overlap on pages to facilitate easier reading for reviewers. The table makes more explicit the stratification, by separating the factors and prior predictors in column two on the table. Factors are grouped with A. while the priori predictors of the three hospitals and both cardiac groups are labelled B and apply to all Guideline Adherence variables. The tables address the research objectives to identify predictors for adherence to the perinatal guidelines for women in both groups pre-existent (PEC) and newly acquired heart (AC) conditions and make comparisons across the three clinical practice settings. | The table has revised titles to help visualise the stratification of both factors and prior predictors that contribute to the uptake of components of the guidelines.  Table 3 and provides stratification of both the factors (A) and the priori predictors of cardiac groups and the three hospital sites (B) that contribute to the uptake of components (outcome variable) of the guidelines. The priori predictors are included in the table for each outcome variable. This has been stated in the table legend on table 3 | Page 16, lines 323- 341  Table 3 |
| Study design evaluating guideline adherence by reviewing medical records has some limitations, and they are commented but how to consider barriers to adherence in this model? The writing and tables (especially table 3) must be reviewed to improve the study comprehension. | The authors acknowledge the limitations of reviewing adherence to guidelines with a retrospective medical review. Given that cardiac disease in pregnancy is under-researched in Australia, this ground-breaking study draws attention to this issue and provides some insight into clinical practice benchmarked against guidelines already in place for women with cardiac conditions in pregnancy the public health system.  Although not explicitly measured in this study Figure 3 was provided to help the reader consider what is already known regarding the implementation of guidelines and resonated with this study. The limited medical record documentation provides some insight into the barriers and facilitators to adherence when documented such as equipment issues or patient non-compliance. Given that this was a retrospective review, over a ten-year time frame there were no feasible methods to measure barriers objectively, e.g. relevant health professional no longer is present in the health care system. | The table has been updated to facilitate easier reading for reviewers, as discussed above.  Although it was not feasible to objectively measure the barriers and facilitators identified in previous research in this retrospective study, the broad categories resonated in this project. The three broad categories of external barriers, patient and health care team factors were considered pertinent see Figure 2 [9, 23, 31]. | Table 3 Page 17  Page 26 Lines 454 – 457. |
| **Reviewer #2: Comments** | | | |
| Reviewer’s #2 comments | Authors comments | Response | Page and line number on Clean copy |
| General comments:  This well-designed study addresses a relevant topic using appropriate statistical analysis. The main limitation, as mentioned by the authors themselves, is the lack of comparison of findings with private institutions. | We acknowledge and thank the reviewer for comments regarding study design, topic relevance and appropriate statistical analysis’s |  |  |
| **Minor comments**  **Abstract** Line 26: - The background was lacking, which justifies the research; | We thank the reviewer for the comment regarding the background to justify the research. The background section has been added to the abstract. | Background:  For pregnant women with a known cardiac condition or those who develop cardiac disease during pregnancy, there is an increased risk of complications during pregnancy, to both mother and foetus. To reduce this risk, best practice guidelines have been developed and available in South Australia for several years. Measuring clinical practice against the guideline recommendations verifies real-life practice and an essential part of any clinical practice quality improvement project by identifying gaps. This study is the first report on adherence to statewide perinatal guidelines for these women in South Australia. | Page 2 Lines 29-35. |
| **Introduction** Line 75: It was not clear to the reviewer which conditions are considered "Cardiac Diseases"; | We acknowledge the comments regarding what conditions are considered cardiac diseases and expanded on the conditions in both introduction and inclusion criteria. | The epidemiology of cardiac disease in pregnancy varies internationally with 0.2-4% estimates; however, it remains under-researched in Australia s reported by Australian Maternity Outcome Surveillance System (AMOSS) [1, 2]. Cardiac disease in pregnancy encompasses a broad spectrum of conditions that include congenital heart disease (CHD), structural and aortic disease, cardiomyopathies, rhythm disorders and pregnancy acquired conditions such as ischemic heart disease (IHD) [3,4]. Cardiac disease is associated with increased rates of morbidity and hospitalisation during pregnancy, with one in four women requiring admission, and is now one of the leading causes of maternal mortality [4-6]. | Page 4, lines 75-82.  Page 6 -7  Lines 137-153. |
| Line 119: Why in the "Pre-existing" group did the authors consider only women with congenital pathologies, eventually disregarding those with hypertensive, ischemic, valvular and cardiomyopathic heart disease acquired before pregnancy? | We acknowledge the reviewer’s comment concerning the pre-existent cardiac groups.  The perinatal audit (S5) provided the two entry for cardiac conditions in pregnancy concerning the guidelines and the literature. The data abstracted from all women did include ischemic, valvular and cardiomyopathic heart disease acquired before pregnancy however the authors did not want to overshadow the focus of this paper, which was to report on adherence to guidelines and predictors within authorship guidelines.  Inclusion criteria have been revised to provide more insight into two cardiac groups. See the relevant section.    Further analysis will include a description of the cardiac conditions in this cohort of women within both cardiac groups, the clinical outcomes (Obstetric, cardiac and neonatal) and association with total adherence score to be submitted as a separate paper.  The Discursive paper by the authors titled Cardiac conditions in pregnancy and the role of midwives: A discussion paper. Nursing Open. 2019 0(23 July 2019). doi: 10.1002/nop2.269 included an overview of cardiac conditions seen in pregnancy.  The authors recognise that women may have acquired cardiac condition before pregnancy that influences their management. Included in the supporting information for this manuscript was the perinatal clinical audit tool (S5) with the question (7) to identify women with a cardiac history in previous pregnancies and the option for detailed comments as text data. The researcher coding notes also included additional cardiac history.  The Perinatal audit [S5] with question 13 for the researcher to broadly categorise the underlying pathological cause of cardiac disease for these women.  Hypertension that may include Essential, Pregnancy-induced hypertension (PIH) and preeclampsia may influence structural and functional changes in the woman’s myocardium that contribute to cardiac complications and so will be reported in the subsequent paper on clinical outcomes i.e. maternal and foetal outcomes.  Question 22 of the perinatal clinical audit identifies those women with hypertension during pregnancy and reports on whether the management approaches. Similarly, the authors acknowledge the reviewer’s comments regarding arrhythmias that encompass a broad spectrum of gravity will be highlighted in the subsequent manuscript and linked to clinical outcomes. | The ‘pre-existing’ group included women with both structural and electrical cardiac conditions before conception. Structural cardiac conditions included cyanotic and acyanotic congenital heart disease, a previous history of hypertensive, valvular and cardiomyopathic heart disease as well as those women with ischemic heart disease. Electrical cardiac conditions included genetically inherited channelopathies as well as the full spectrum of acquired bradyarrhythmias and tachyarrhythmias. Women with symptomatic benign ectopic beats were excluded from this group. | Page 6, lines 139 -145 |
| Lines 121-124:  The defining criteria for the "New Acquired" group are mixed: Acute Coronary Syndromes (ACS) include ST-segment elevation acute myocardial infarction, ST-segment elevation acute myocardial infarction, and unstable angina; therefore, ischemic heart disease (IHD) is a form of ACS; "Angina" refers to Chronic Coronary Syndromes ?; On the other hand, "Cardiomyopathies" acquired during pregnancy can be confused with pre-existing undiagnosed diseases. Do the authors refer to "Peripartum Cardiomyopathy"? And any "myocarditis"; the occurrence of "Pregnancy Specific Hypertensive Disease" was not considered; and finally "arrhythmia" has a broad meaning, encompassing a broad spectrum of gravity. | We acknowledge the reviewer’s comment concerning the newly acquired cardiac groups. As above with supporting information (S5) provided the two entry for cardiac conditions in pregnancy concerning the guidelines and the literature.  The data abstracted from all women did include other cardiomyopathies, i.e. idiopathic hypertrophic and peripartum ischemic, valvular and arrhythmias acquired during pregnancy however the authors did not want to overshadow the focus of this paper, which was to report on adherence to guidelines and predictors within authorship guidelines. The authors recognise that pregnancy may unmask pre-existent cardiac conditions which were evident in this study and will be addressed in the subsequent manuscript linking clinical outcomes, and total adherence score.  Included in the supporting information for this manuscript was the Perinatal clinical audit tool (S5) authors draw your attention to :   - Question 7 which identifies those women with a cardiac history in previous pregnancies with the option of further detailed comments. Also coding notes expanded on cardiac history. - Question 13 allowed coding of the pathological cause of cardiac disease at the entry point for these women. Hypertension that may be essential, pregnancy-induced hypertension and preeclampsia that may contribute to cardiac complications such as structural and functional changes in the woman’s myocardium will be included in clinical outcomes to be reported in a subsequent manuscript. - Question 22 identified those women with hypertension during pregnancy and reports on the management approaches. - Similarly, arrhythmias encompassing a broad gravity, as discussed in the previous publications will be addressed in the future manuscript. However table 1 has been revised to include points raised. | Similarly, women in whom undiagnosed congenital cardiac conditions were unmasked during pregnancy were included in the ‘newly acquired group’.  The ‘newly acquired’ group included women with pregnancy-associated cardiac events.  This includes any new symptomatic sustained arrhythmia, the unmaking of underlying prior cardiac conditions previously unrecognised, and new-onset cardiac disease such as acute coronary syndrome, cardiomyopathy with or without decompensated heart failure, and infective endocarditis during pregnancy or up to 12 months postpartum. These inclusion criteria were similar to those used in the United Kingdom survey of obstetric deaths [19]. | Pages 6 -7. Lines 145- 153. |
| Outcome Variables. Line 149: Were the scores used in the present investigation validated for the Australian population? | We thank the reviewer for the comment regarding the present investigations validated for the Australian population. This is the first study to evaluate the of adherence SA statewide guidelines pertinent to cardiac disease in pregnancy and so authors cannot compare with the Australian population. A review of the literature yielded no Australian studies. However, a prospective study that focuses on the states of NSW and ACT through Australasian Maternity Outcomes Surveillance System (AMOSS) from 2019 -2020 is currently in progress can be accessed via the link is below.  <https://www.amoss.com.au/content/cardiac-disease-pregnancy> |  |  |
| Predictor Variables. Line 169:  Why were not included in the "Maternal Characteristics", hemodynamic variables such "Systemic Blood Pressure" and "Heart Rate"? | We acknowledge the reviewer’s comment on the inclusion of haemodynamic variables in maternal characteristics. A retrospective medical review provides limited opportunity to obtain the patient’ consent and availability to the individual’s data. The researcher did collect the Systemic blood pressure on admission; however, this may not reflect the blood pressure at the time of diagnosis. |  |  |
| Results Line 285: What is the meaning of "Hypertension"? Do the authors refer to "Pulmonary Hypertension" or "Systemic Arterial Hypertension"? | We thank the reviewer for the comment regarding the meaning of hypertension. The manuscript text is revised to reflect the term systemic arterial hypertension with a severe cardiac condition. | IHD and systemic arterial hypertension with severe cardiac conditions (equally n=10, 4%) were less frequently reported | Page 12 line 269. |
| Discussion Line 400: I suggest including a comment of this type: We can speculate that Guidelines should contain realistic and clear recommendations, to be a room for individual judgement clinic and orientated for the patient as for efficacy versus safety | We acknowledge the reviewer’s comment and have included the following sentence in the relevant paragraph, with one change “propose’ instead of speculate. The authors propose that perinatal guidelines should contain realistic and clear recommendations, which allow individual clinical judgement orientated for the patient as for efficacy versus safety. The authors have expanded on this comment with preceding salient points related to preconception counselling and initial comprehensive antenatal assessment included in the relevant section. | The actions undertaken comprehensively before or early in the pregnancy resulted in ongoing adherence to the guidelines. The observed variance in the level of adherence across the three hospitals reflected the exposure to higher acuity cases, and that appropriate referral was intrinsically linked to better adherence. The early inclusion of the multidisciplinary team facilitated adherence to the guidelines, mainly with preconception care, and echocardiogram assessment of cardiac status.  The authors propose that perinatal guidelines should contain realistic and clear recommendations, which allow individual clinical judgement orientated for the patient as for efficacy versus safety. Two pivotal recommendations are preconception counselling and a comprehensive initial antenatal assessment. Therefore, it is vital to ensure that all health practitioners have the skills, training and sufficient time to complete the initial assessment [3]. | Page 28 Lines 495-500. |
